# Functional implications of the exon 9 splice insert in GluK1 kainate receptors

**DOI:** 10.7554/eLife.89755

**Published:** 2024-11-06

**Authors:** Surbhi Dhingra, Prachi M Chopade, Rajesh Vinnakota, Janesh Kumar

**Affiliations:** 1 Laboratory of Membrane Protein Biology, National Centre for Cell Science Pune India; 2 Laboratory of Membrane Protein Biology, CSIR-Centre for Cellular and Molecular Biology Hyderabad India; University of Kansas Medical Center United States; Stanford University United States

**Keywords:** kainate receptors, alternative splicing, electrophysiology, Cryo-EM, Rat

## Abstract

Kainate receptors are key modulators of synaptic transmission and plasticity in the central nervous system. Different kainate receptor isoforms with distinct spatiotemporal expressions have been identified in the brain. The GluK1-1 splice variant receptors, which are abundant in the adult brain, have an extra fifteen amino acids inserted in the amino-terminal domain (ATD) of the receptor resulting from alternative splicing of exon 9. However, the functional implications of this post-transcriptional modification are not yet clear. We employed a multi-pronged approach using cryogenic electron microscopy, electrophysiology, and other biophysical and biochemical tools to understand the structural and functional impact of this splice insert in the extracellular domain of GluK1 receptors. Our study reveals that the splice insert alters the key gating properties of GluK1 receptors and their modulation by the cognate auxiliary Neuropilin and tolloid-like (Neto) proteins 1 and 2. Mutational analysis identified the role of crucial splice residues that influence receptor properties and their modulation. Furthermore, the cryoEM structure of the variant shows that the presence of exon 9 in GluK1 does not affect the receptor architecture or domain arrangement in the desensitized state. Our study thus provides the first detailed structural and functional characterization of GluK1-1a receptors, highlighting the role of the splice insert in modulating receptor properties and their modulation.

## Introduction

Kainate receptors (KARs), a subfamily of ionotropic glutamate receptors (iGluRs), are required in the vertebrate brain for postsynaptic neurotransmission and presynaptic regulation of transmitter release (Erreger et al., 2004; Huettner, 2003; Lerma, 2003; Pinheiro and Mulle, 2006). They are known to mediate characteristic small-amplitude excitatory postsynaptic currents (EPSC) with slow kinetics in the hippocampal regions of the central nervous system compared to their counterparts, α-amino-3-hydroxy-5-methyl-4-isoxazole propionic acid (AMPA) and N-methyl-D-aspartate (NMDA) receptors (Castillo et al., 1997; Lerma et al., 2001; Traynelis et al., 2010). Furthermore, they play a vital role in the maturation of neuronal circuits during development by interacting with G proteins (Lerma, 2003; Lerma and Marques, 2013). Any functional defect predisposes the brain to various disorders, such as autism, epilepsy, schizophrenia, and neuropathic pain (Lerma and Marques, 2013; Bowie, 2008; Valbuena and Lerma, 2021).

Kainate receptors are composed of subunits from two gene families: low kainate affinity and high kainate affinity. The first gene family includes GluK1-GluK3, which can form both homomeric and heteromeric receptors with subunits from the same family or from the high-kainate affinity family. On the other hand, GluK4-GluK5 are high-affinity subunits that must assemble with low-affinity subunits to create functional receptors. The assembly of different subunits results in the formation of various receptor configurations, which contribute to the wide range of functional properties observed in kainate receptors.

The functional repertoire of kainate receptors is further enhanced by RNA editing (Barbon and Barlati, 2011; Seeburg, 2002) and alternative splicing (Jaskolski et al., 2004). In particular, GluK1-containing KARs mainly present in the hippocampus, cortical interneurons, Purkinje cells, and sensory neurons undergo alternative splicing and RNA editing (Bernard and Khrestchatisky, 1994). Alternative splicing of the C-terminal domain that produces four isoforms, GluK1-a (shortest C-terminal), GluK1-b, GluK1-c, and GluK1-d (Bettler et al., 1990; Gregor et al., 1993; Pinheiro and Mulle, 2006; Sommer et al., 1992) has been studied (Ren et al., 2003). However, the functional impact of the mRNA splicing of the N-terminal domain (exon 9) resulting in isoforms GluK1-1 and GluK1-2, with GluK1-1 containing an additional 15 amino acids in the amino-terminal domain (ATD) is not understood. Interestingly, the splice junctions for GluK1-1 were reported to be more frequent than those for GluK1-2, and both variants showed similar expression in different species (Herbrechter et al., 2021). This 15-amino acid insertion in the ATD is exclusive to the GluK1 subunit and may impart unique properties to KARs containing this variant. Therefore, investigations of functional differences between GluK1 receptors with and without the 15 amino acid splice insert are necessary but missing in the field. Furthermore, the impact of the N-terminal splice insert in GluK1-1 receptors on its modulation by cognate auxiliary subunits (Fisher, 2015; He et al., 2021; Sheng et al., 2015; Vinnakota et al., 2021), neuropilin and tolloid-like (Neto) proteins is also unknown.

Therefore, we conducted structure-function studies to understand the mechanisms of GluK1-1a receptor function and the effects of the ATD splice on receptor assembly, stability, and modulation by Neto proteins. Whole-cell and excised outside-out patch-clamp-based functional assays were performed to investigate the differences between the ATD splice variants GluK1-1a (exon 9) and GluK1-2a (lacking exon 9) and their modulation by Neto proteins. Furthermore, mutational analysis was performed to identify important splice residues that affect receptor functions and their modulation by Neto proteins. Additionally, we expressed and purified rat GluK1-1a from HEK293 GnTI- cells and determined the single-particle cryo-EM structures of the receptors trapped in the desensitized state to understand the effect of splice on receptor architecture.

## Results

### Spatiotemporal expression pattern of GluK1-1 in the brain

**Figure 1.**
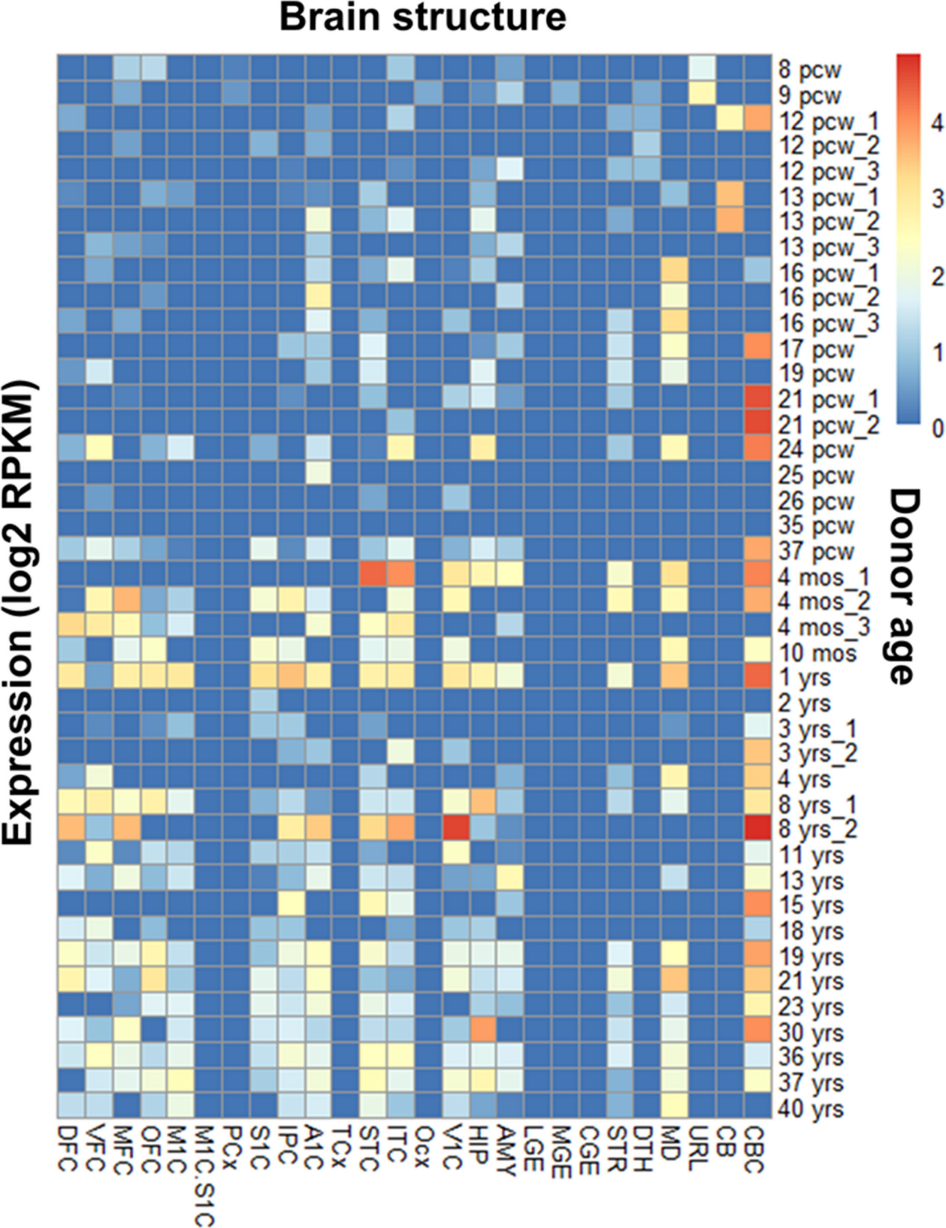
RNA-seq analysis (BrainSpan atlas) demonstrates an abundance of *GRIK1* exon 9 in the human brain. The heat map shows the presence of exon 9 (45 bp; ENSE00001313812) that codes for GluK1 amino-terminal domain (ATD) splice in different brain regions from the embryonic to adult stage. Exon 9 expression coincides with well-studied areas for the GRIK1 gene like the cerebellar cortex, visual cortex, etc. Various regions of the brain and the donor age are represented on the x-axis and y-axis, respectively. The donor age has been abbreviated as pcw (post-conception weeks), mos (months), and yrs (years). The regions of the human brain are abbreviated as: DFC (dorsolateral prefrontal cortex), VFC (ventrolateral prefrontal cortex), MFC (anterior [rostral] cingulate [medial prefrontal] cortex), OFC (orbital frontal cortex), M1C (primary motor cortex area M1, area 4), M1C.S1C (primary motor-sensory cortex [samples]), PCx (parietal neocortex), S1C primary somatosensory cortex (area S1, areas 3,1,2), IPC (posteroventral [inferior] parietal cortex), A1C (primary auditory cortex core), A1C (primary auditory cortex [core]), TCx (temporal neocortex), STC (posterior [caudal] superior temporal cortex, area 22 c), ITC (inferolateral temporal cortex) (area TEv, area 20), OCx (occipital neocortex), V1C primary visual cortex (striate cortex, area V1/17), HIP (hippocampus), AMY (amygdaloid complex), LGE (lateral ganglionic eminence), MGE (medial ganglionic eminence), CGE (caudal ganglionic eminence), STR (striatum), DTH (dorsal thalamus), MD (mediodorsal nucleus of thalamus), URL (upper [rostral] rhombic lip), CB (cerebellum), and CBC (cerebellar cortex). Blue and red color indicates zero and maximum expression, respectively. Figure 1—source data 1.RNA-seq analysis data for GRIK1 exon 9.

Kainate receptor subunits are differentially and spatially regulated in vertebrates. We analyzed publicly available human transcriptomics data to determine whether the exon encoding the GluK1 ATD splice (exon 9) was differentially expressed in the human brain. RNASeq data analysis of *GRIK1* (Ensembl ID: ENSG00000171189) collected from the BrainSpan ATLAS indicated that exon 9 is present in multiple brain areas that also show prominent GluK1 expression. GRIK1 exon 9 expression varies significantly across brain regions and developmental stages, with dynamic patterns indicating its crucial role in brain development. High expression is noted during critical periods such as early embryonic, postnatal, and childhood stages, particularly in regions like the cortical plate (CP), hippocampus (HIP), amygdala (AMY) and striatum (STR) (Figure 1; Figure 1—figure supplement 1). These patterns suggest GRIK1 exon 9’s importance in the functional maturation of these brain regions. For example, in the cerebellar cortex, GRIK1 exon 9 expression peaks during early development and childhood and stabilizes into adulthood, underscoring its role in cortical development and function. (Figure 1—figure supplement 1). The significant variability in exon 9 expression across different brain regions, along with its potential role in brain development and functional maturation, motivated us to investigate the underlying molecular mechanisms. This prompted us to perform a detailed structure-function analysis of the GluK1-1a splice variant.

**Figure 1—figure supplement 1.**
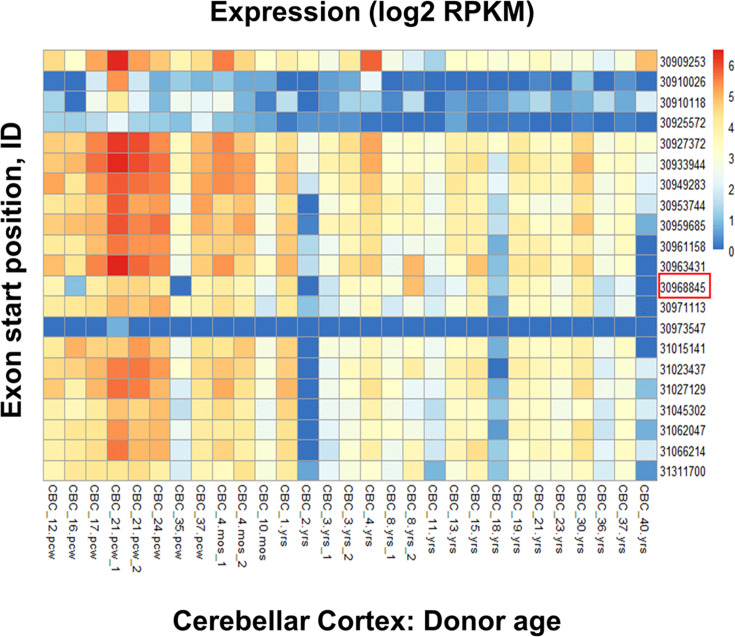
Representative RNA-seq analysis (BrainSpan atlas) of the cerebellar cortex (CBC) demonstrates the high expression of exon 9 in the human brain. To delve deeper into the high expression areas from Figure 1, the cerebellar cortex was chosen as an example to understand the expression pattern of Exon 9 with respect to other GluK1 exons. The heat map displays the abundance of the GluK1 exons at different stages of human life. Exon 9 (Exon start position: 30968845; marked by a red box) expression coincides with reported high expression of GluK1 kainate receptors in the early developmental stages of humans. Blue and red color indicates zero and maximum expression, respectively. Figure 1—figure supplement 1—source data 1.RNA-seq analysis data for GRIK1 exon 9 for cerebellar cortex (CBC).

### The ATD splice imparts functional diversity to GluK1 receptors

**Figure 2.**
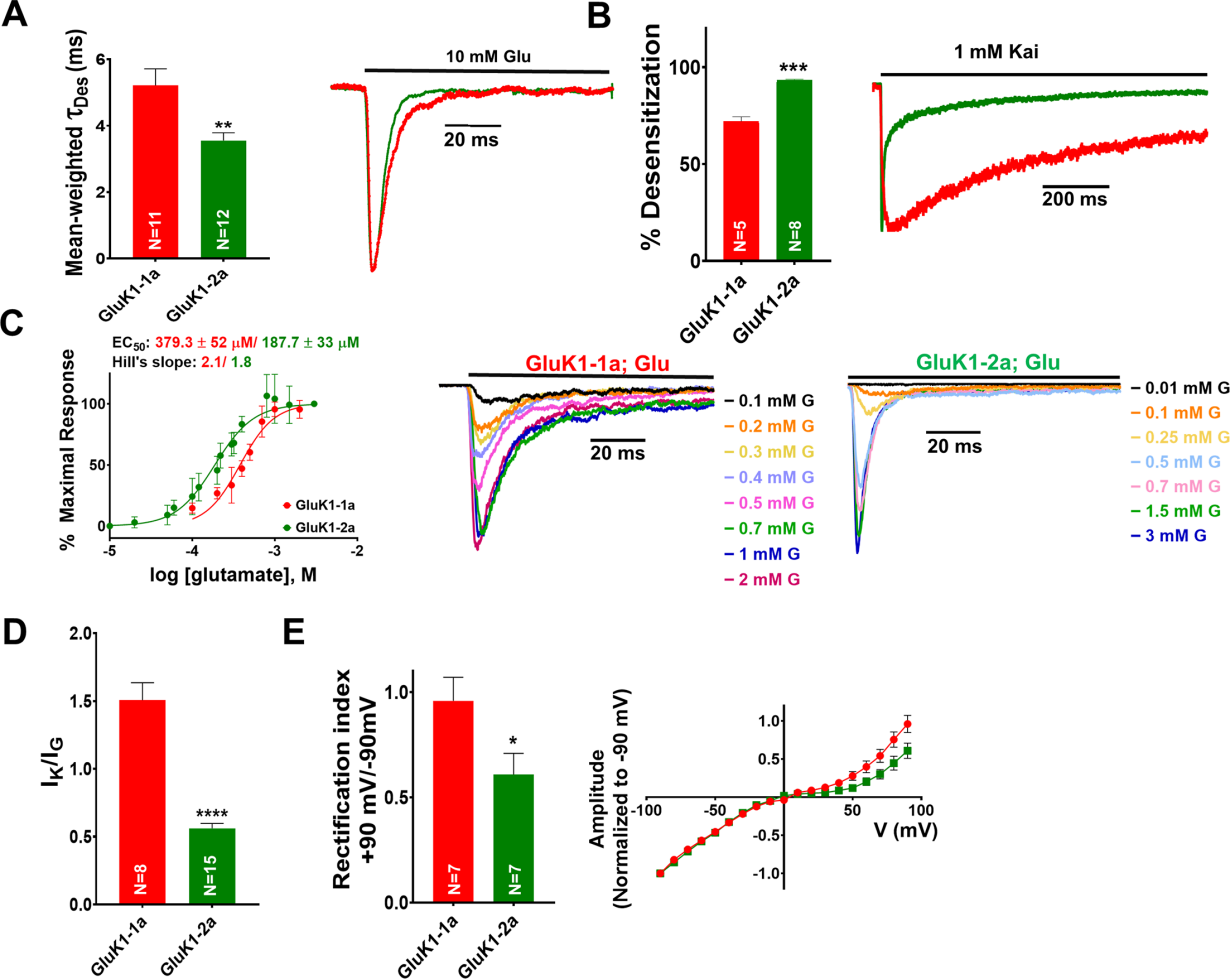
Amino-terminal domain (ATD) splice insert affects the gating properties of the GluK1-1a homomeric receptors. (**A**) Displays mean-weighted Tau (τ_Des_) values for GluK1-1a wild-type (red) and GluK1-2a wild-type (green) in the presence of glutamate. The inset shows representative normalized traces for GluK1-1a and GluK1-2a with 10 mM glutamate. (**B**) Displays the percent desensitization values calculated at 1 s for GluK1-1a wild-type (red) and GluK1-2a wild-type (green) in the presence of kainate. Representative normalized traces for GluK1-1a and GluK1-2a with 1 mM kainate are shown. (**C**) Demonstrates glutamate dose-response curves for GluK1-1a and GluK1-2a. Representative aligned traces for both receptors at various glutamate concentrations are shown. The kainate dose responses for the splice variants are shown in Figure 2—figure supplement 1. (**D**) The ratio of currents evoked by kainate and glutamate is plotted for GluK1-1a and GluK1-2a. (**E**) The ratio of currents evoked by the application of 10 mM glutamate at +90 mV and –90 mV for the GluK1-1a and GluK1-2a receptors is shown. Representative IV plots are depicted for GluK1-1a and GluK1-2a for the entire voltage ramp (–90 to +90 mV). Error bars indicate mean ± SEM, N in each bar represents the number of cells used for analysis, and * indicates the significance at a 95% confidence interval. Figure 2—source data 1.Data used for the electrophysiology plots.

Previous studies on functional analysis of GluK1 receptors have primarily focused on GluK1-2 isoform that lacks the ATD splice insert. Therefore, we conducted an extensive electrophysiological investigation to assay the functional differences between GluK1-1a and GluK1-2a. The only difference between these two variants is the presence of 15 amino acids (KASGEVSKHLYKVWK) insertion in the R2 subdomain (lower lobe) of the GluK1-1a ATD. We evaluated multiple gating properties of the two variants by whole-cell patch-clamp electrophysiology. Interestingly, we observed that GluK1-1a receptors desensitize significantly slowly when compared to GluK1-2a (Ʈ_Des_, GluK1-1a: 5.21±0.50 ms; GluK1-2a: 3.55±0.23 ms **p=0.0092) on prolonged treatment with 10 mM glutamate (Figure 2A; Table 1*)*. We also tested receptor desensitization by applying saturating concentrations of kainate (1 mM). Since the receptors displayed prolonged desensitizing kainate currents, instead of the rate (Ʈ_Des_), we calculated the % desensitization values at 1 s for comparison. We observed that the % desensitization with kainate was distinct for both ATD splice variants with significantly slower desensitization in the variant with the splice insert (GluK1-1a: 72.06±2.33%, GluK1-2a: 93.2±0.55 ***p=0.0006) consistent with the glutamate evoked currents (Figure 2B; Table 1*)*.

**Figure 2—figure supplement 1.**
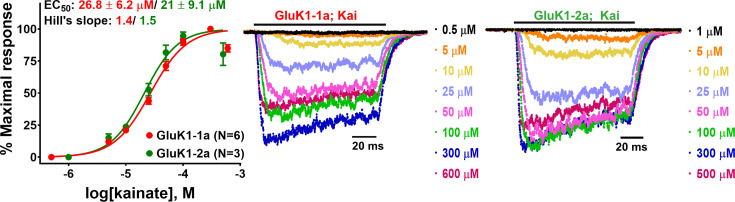
Kainate-evoked responses for GluK1 receptors. The figure demonstrates the normalized kainate dose-response curves for GluK1-1a and GluK1-2a along with their representative traces. Figure 2—figure supplement 1—source data 1.Data used for the electrophysiology plots.

**Table 1.** Whole-cell patch clamp recordings of GluK1-1a, GluK1-1a_EM_, GluK1-2a, and GluK1-1a mutants in the absence or presence of Neto1 (green) or Neto2 (peach). Errors are reported as SEM. Statistical significance is reported at 95 % CI. p<0.05 (*), p<0.01 (**), p<0.001 (***), p<0.0001 (****) for comparisons between wild-type GluK1-1a receptor with EM construct, GluK1-2a, or various mutants in presence or absence of Neto proteins. ‘a’ denotes the lack of rectification index value due to no conductance observed at positive potentials in the K_368_-E mutant alone.

S. No.	Name of the construct	Desensitization					Total No. of cells tested
		10 mM G (Ʈ_Des_ in ms)	1 mM K (%)	I_K_/I_G_	Recovery (Ʈ_Rec_ in s)	Rectification (+90 mV/–90 mV)	
1	GluK1-1a_EM_	1.94+0.13 N=8 (****p<0.0001)	-	-	2.63±0.04 N=3 (p=0.3501)	-	23
2	GluK1-1a	5.21±0.50 N=11	72.06±2.33 N=5	1.51±0.13 N=8	3.53±0.81 N=4	0.96±0.11 N=7	19
	+Neto1	3.56±0.22 N=9 (**p=0.0090)	49.61±5.23 N=7 (**p=0.0075)	1.25±0.04 N=8 (p=0.1500)	0.68±0.07 N=8 (*p=0.0390)	1.16±0.09 N=7 (p=0.1880)	11
	+Neto2	69.62±9.98 N=6 (**p=0.0024)	68.96±4.36 N=9 (p=0.7459)	1.00±0.07 N=13 (**p=0.0096)	8.32±0.81 N=5 (**p=0.0044)	0.80±0.05 N=4 (p=0.2401)	17
3	GluK1-2a	3.55±0.23 N=12 (**p=0.0092)	93.20±0.55 N=8 (***p=0.0006)	0.56±0.04 N=15 (****p<0.0001)	5.31±0.50 N=7 (p=0.1191)	0.61±0.10 N=7 (*p=0.0385)	21
	+Neto1	4.32±0.34 N=8 (p=0.1495)	76.99±3.41 N=6 (**p=0.0085)	1.37±0.12 N=10 (****p<0.0001)	1.15±0.12 N=5 (***p=0.0002)	1.14±0.14 N=3 (*p=0.0338)	14
	+Neto2	21.68±2.64 N=6 (**p=0.0017)	65.98±2.41 N=7 (****p<0.0001)	1.16±0.04 N=8 (****p<0.0001)	7.91±0.71 N=4 (*p=0.0430)	1.37±0.10 N=3 (**p=0.0022)	18
4	K_375_H_376_-A	3.80±0.33 N=5 (*p=0.0332)	71.07±5.27 N=5 (p=0.9998)	1.43±0.07 N=5 (p=0.6195)	1.10±0.22 N=4 (**p=0.0095)	0.78±0.05 N=4 (p>0.9999)	9
	+Neto1	3.38±0.14 N=8 (p=0.4947)	54.64±5.18 N=6 (p=0.5086)	1.76±0.14 N=8 (**p=0.0070)	1.15±0.11 N=9 (**p=0.0026)	0.64±0.04 N=4 (***p=0.0008)	10
	+Neto2	42.57±11.96 N=4 (p=0.1280)	61.03±5.12 N=5 (p=0.6416)	1.02±0.02 N=5 (*P*p0.7791)	9.32±0.15 N=3 (p=0.6286)	0.60±0.06 N=5 (p=0.1138)	6
5	K_375/379/382_ A	Low peak amplitudes (≤40 pA)					15
	+Neto1	2.82±0.18 N=6	64.13±5.26 N=5	1.42±0.08 N=6	0.74±0.08 N=6	1.28±0.10 N=6	8
	+Neto2	Low peak amplitudes (≤40 pA)					27
6	Y_378_V_380_W_381_-A	Low peak amplitudes (≤40 pA)					18
	+Neto1	3.31±0.16 N=9	71.48±4.66 N=5	1.76±0.21 N=7	1.02±0.07 N=7	0.77±0.14 N=5	10
	+Neto2	45.79±17.32 N=3	51.82±4.88 N=3	1.19±0.11 N=3	9.45±1.21 N=3	1.03±0.44 N=4	22
7	K_375_-K_382_-8A	Non-functional N=15	Non-functional N=5	-	-	Non-functional N=5	15
	+Neto1	Non-functional N=11	Non-functional N=4	-	-	Non-functional N=5	11
	+Neto2	Non-functional N=11	Non-functional N=5	-	-	Non-functional N=6	11
8	K_375_H_376_-E	Low peak amplitudes (≤40 pA)					13
	+Neto1	3.59±0.29 N=4	13.78±3.16 N=5	0.70±0.19 N=4	0.63±0.10 N=3	0.68±0.19 N=3	23
	+Neto2	Low peak amplitudes (≤40 pA)					36
9	H_376_-E	5.07±0.42 N=7 (p=0.8408)	29.80±13.21 N=4 (*p=0.0471)	0.88±0.05 N=3 (*P=0.0272)	1.48±0.18 N=8 (*p=0.0112)	0.44±0.07 N=6 (**p=0.0023)	19
	+Neto1	2.69±0.15 N=5 (**p=0.0065)	59.24±8.62 N=4 (p=0.3813)	2.36±0.30 N=4 (*P=0.0328)	0.66±0.05 N=5 (p=0.7939)	0.80±0.19 N=4 (p=0.3625)	6
	+Neto2	50.88±6.49 N=4	28.82±6.43 N=5 (**p=0.0034)	1.43±0.13 N=4 (*P=0.0347)	4.98±0.81 N=3 (*p=0.0237)	0.42±0.08 N=8 (**p=0.0056)	50
10	K_375/379/382_H_376_-E	9.62±1.47 N=6 (*p=0.0290)	54.25±8.28 N=3 (*p=0.0396)	1.17±0.28 N=3 (*P*=0.3733)	4.83±0.31 N=3 (p=0.2774)	0.62±0.14 N=7 (*p=0.0499)	36
	+Neto1	3.74±0.20 N=8 (p=0.5390)	57.72±12.65 N=3 (p=0.5996)	1.69±0.15 N=6 (*p=0.0326)	0.66±0.08 N=8 (p=0.8702)	1.23±0.07 N=6 (p=0.5351)	9
	+Neto2	65.57±17.04 N=8 (p=0.8412)	26.66±2.05 N=4 (****p<0.0001)	0.86±0.08 N=4 (p=0.2045)	6.57±0.61 N=3 (p=0.3588)	0.72±0.09 N=4 (p=0.6677)	48
11	K_368/375/379/382_H_376_-E	Low peak amplitudes (≤40 pA)					20
	+Neto1	4.86±0.67 N=5 (p=0.1242)	10.39±.3.53 N=5	1.15±0.20 N=4 (p=0.6495)	0.21±0.12 N=3	0.59±0.13 N=5 (**p=0.0081)	21
	+Neto2	Low peak amplitudes (≤40 pA)					22
12	K_368_-E	7.89±1.14 N=3 (*p=0.0334)	46.66±5.88 N=5 (**p=0.0093)	0.81±0.13 N=5 (**p=0.0037)	5.61±0.95 N=3 (*p=0.0417)	a N=7 (****p<0.0001)	32
	+Neto1	4.37±0.25 N=4 (*p=0.0393)	59.68±8.72 N=3 (p=0.3851)	6.99±0.47 N=5 (***p=0.0002)	0.43±0.05 N=6 (*p=0.0118)	2.84±0.16 N=3 (**p=0.0021)	8
	+Neto2	79.40±4.44 N=5 (p=0.4012)	33.47±8.98 N=5 (*p=0.0388)	2.13±0.13 N=4 (***p=0.0007)	7.66±0.83 N=3 (p=0.9473)	0.76±0.26 N=8 (p=0.9860)	43

Next, we evaluated glutamate sensitivity for the two variants. Dose-response experiments (glutamate, GluK1-1a: 0.1–2 mM, and GluK1-2a: 0.01–3 mM) showed a significantly lower potency of glutamate for GluK1-1a variant compared to the non-spliced form (EC_50_ glutamate), GluK1-1a: 379.3±52 µM, GluK1-2a: 187.7±33 µM *p=0.0129 (Figure 2C; Table 1). However, for high-affinity agonist kainate, the dose-response curves for both variants were similar, likely indicating differences in the stability of glutamate *versus* the kainate-bound states in GluK1-1a and GluK1-2a (Figure 2D; Figure 2—figure supplement 1). Thus, the potency of kainate (1 mM) *versus* glutamate (10 mM) (I_K_/I_G_ ratio) is significantly higher for GluK1-1a compared to GluK1-2a (GluK1-1a: 1.51±0.13, GluK1-2a: 0.56±0.4 ****p<0.0001) (Figure 2D; Table 1).

Furthermore, we investigated the voltage-dependent endogenous polyamine block and found significant differences between the two variants. The presence of splice residues seemed to enhance outward currents at positive potentials in GluK1-1a compared to GluK1-2a (rectification index,+90 mV/–90 mV; GluK1-1a=0.96 ± 0.11; GluK1-2a=0.61 ± 0.10 *p=0.0385) without affecting the reversal potential (Figure 2E; Table 1). How splice residues situated ~92 Å away from the TM domain (distance between atoms W381 CA in the ATD and L636 CA in the TM3) affect the pore properties is unclear. Earlier reports suggest that rectification in KARs is mainly affected by the TM2 region (Bowie and Mayer, 1995). However, a recent report in which the ATD of GluK2 was deleted also showed enhanced rectification (Li et al., 2019). Splice residues likely alter pore structure by allosteric mechanisms that have (are) yet to be identified, thereby affecting rectification (Perrais et al., 2009). We also performed outside-out patch recordings to examine GluK1-1a receptors. However, we observed extremely weak electrical currents with low amplitudes when GluK1-1a was expressed in isolation. Consequently, the data we obtained from these recordings did not provide reliable results for curve fitting or thorough analysis.

### ATD splice insert impacts GluK1 receptor modulation by Neto proteins

**Figure 3.**
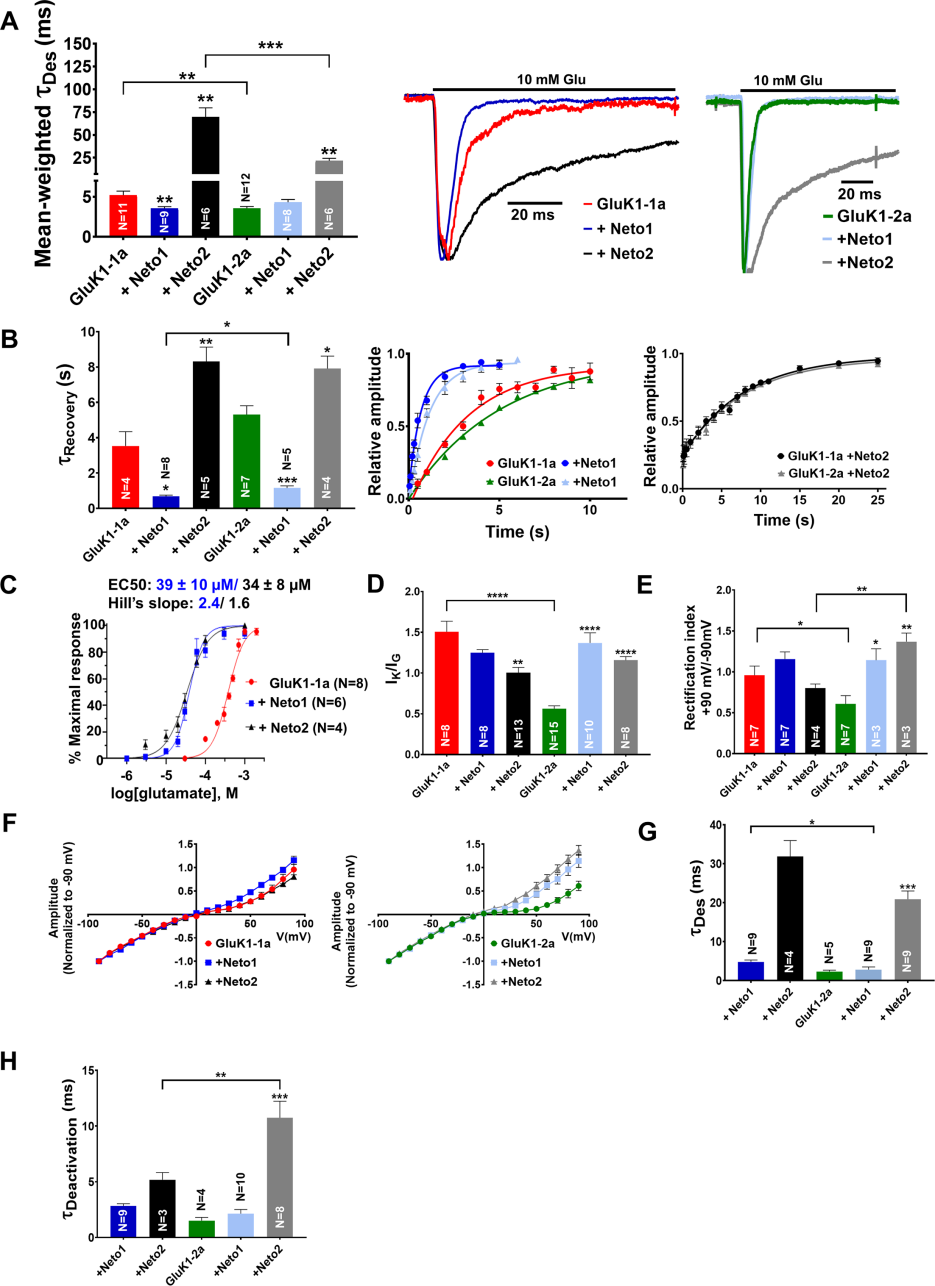
Amino-terminal domain (ATD) splice affects the functional modulation of GluK1 kainate receptors by Neto proteins. (**A**) Shows mean-weighted Tau (τ_Des_) values calculated at 100 ms for GluK1-1a (red) and GluK1-2a (green), respectively, with full-length Neto1 (blue/light blue) or Neto2 (black/gray), in the presence of glutamate. Representative normalized traces are shown for 100 ms application of 10 mM glutamate for HEK293 cells co-expressing GluK1-1a or GluK1-2a with Neto1 and Neto2. (**B**) Shows Tau (τ_Recovery_) values plotted for GluK1-1a and GluK-2a, respectively, with full-length Neto1 or Neto2. Relative amplitude graphs for each receptor in the absence or presence of Neto proteins are also depicted. (**C**) Demonstrates the glutamate dose-response curves for GluK1-1a with Neto proteins. (**D**) Indicates the ratio of peak amplitudes evoked in the presence of 1 mM kainate and 10 mM glutamate for GluK1-1a or GluK1-2a with or without Neto proteins. (**E**) The ratio of currents evoked by the application of 10 mM glutamate at +90 mV and –90 mV for the receptors in the absence or presence of Neto proteins is shown. (**F**) Shows representative IV plots for GluK1-1a and GluK1-2a for the receptor alone versus with Neto proteins, respectively. Panels (**G**) and (**H**) show data recorded from outside-out pulled patches. (**G**) Displays desensitization kinetics for GluK1-1a (red) and GluK1-2a (green) with or without Neto proteins, respectively. (**H**) Shows deactivation kinetics at 1 ms for GluK1-1a (red) and GluK1-2a (green) with or without Neto proteins. Error bars indicate mean ± SEM, N in each bar represents the number of cells used for analysis, and * indicates the significance at a 95% confidence interval. Figure 3—source data 1.Data used for the electrophysiology plots.

Neto 1 and Neto 2 proteins significantly influence the surface expression, synaptic localization, and functional properties of the GluK1-2a receptors (Copits et al., 2011; Palacios-Filardo et al., 2016; Sheng et al., 2015). It has been demonstrated that desensitization of GluK1-2a is accelerated by Neto1 but delayed by Neto2 (Sheng et al., 2015; Copits et al., 2011; Palacios-Filardo et al., 2016). Hence, to understand the influence of these KAR auxiliary proteins on GluK1-1a, we performed an electrophysiological analysis of GluK1-1a and GluK1-2a receptors co-expressed with either Neto1 or Neto2. Co-expression of Neto1 hastened desensitization of GluK1-1a significantly but not of GluK1-2a at saturating glutamate concentrations (Ʈ_Des_, GluK1-1a +Neto1: 3.56±0.22 ms **p=0.0090; GluK1-2a + Neto1: 4.32±0.34 p=0.1495). On the other hand, Neto2 led to a ~13.4 fold decrease in the desensitization rate of GluK1-1a (Ʈ_Des_, GluK1-1a: 5.21±0.50 ms, +Neto2: 69.62±9.98 ms **p=0.0024) while the desensitization rate of GluK1-2a was decreased only by ~6.1 fold (Ʈ_Des_, GluK1-2a: 3.55±0.23 ms, +Neto2: 21.68±2.64 ms **p=0.0017). Thus, while Neto1 accelerated the desensitization of GluK1-1a, Neto2 significantly slowed it at saturating glutamate concentrations. The rate of desensitization for GluK1-1a was approximately 3.2 times slower compared to GluK1-2a (***p=0.0009) when coexpressed with Neto2 (Figure 3A; Table 1). Thus, the presence of splice insert leads to differential modulation of GluK1 desensitization by Neto proteins.

**Figure 3—figure supplement 1.**
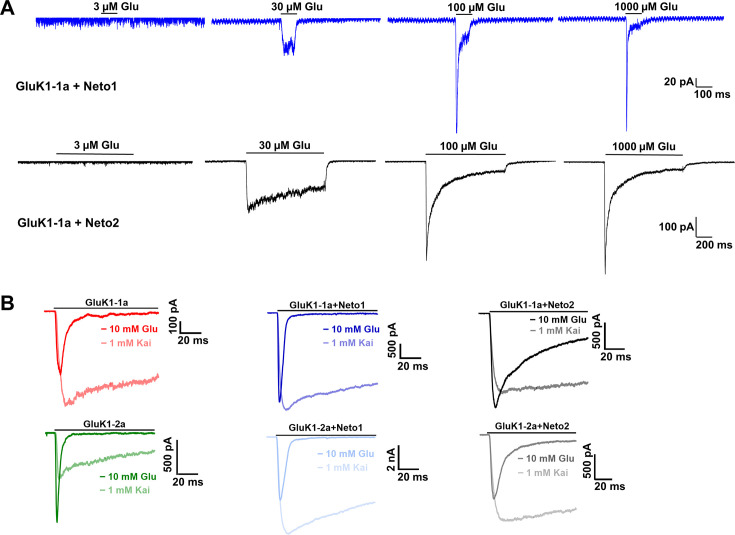
Effect of Neto proteins on agonist-evoked responses of GluK1 receptors. (**A**) Displays representative traces for GluK1-1a in the presence of Neto1 (blue) or Neto2 (black) with varying concentrations of glutamate (3 µM, 30 µM, 100 µM, and 1000 µM). Ligand application for receptors with Neto1 and Neto2 was 100 ms, and 1000 ms, respectively. (**B**) Representative 100ms traces for currents evoked in the presence of glutamate vs. kainate in the absence and presence of Neto proteins. Figure 3—figure supplement 1—source data 1.Data used for the electrophysiology plots.

Moreover, Neto1 also enhanced the recovery from desensitization for both the variants (Ʈ_Recovery_, GluK1-1a: 3.53±0.81 s, +Neto1: 0.68±0.07 s *p=0.0390; GluK1-2a: 5.31±0.50 s +Neto1: 1.15±0.12 s ***p=0.0002). GluK1-1a recovers ~1.7 times faster than GluK1-2a (*p=0.0125) when co-expressed with Neto1. Neto 2, on the other hand, slowed recovery for both variants to a similar extent (Ʈ_Recovery_, GluK1-1a: 3.53±0.81 s, +Neto2: 8.32±0.81 s **p=0.0044; GluK1-2a: 5.31±0.50 s, +Neto2: 7.91±0.71 s *p=0.0430) and did not show differential modulation (Figure 3B; Table 1). In addition, both Neto1 and Neto2 increased the potency of glutamate for GluK1-1a by 9.7-fold (39±10 µM) and 11.2-fold (34±8 µM), respectively (Figure 3C; Figure 3—figure supplement 1A). This is similar to the effect observed in GluK1-2a receptors whereby the glutamate EC_50_ was shown to increase by Neto proteins Neto1: 34-fold and Neto2: 7.5-fold (Palacios-Filardo et al., 2016) and Neto1/2: 30–10 X (Fisher, 2015).

Since we observed a significant increase in the I_K_/I_G_ ratios in GluK1 due to the presence of splice insert, we next aimed to determine the influence of co-expressing Neto proteins on this parameter. Interestingly, while Neto 1 and Neto2 reduced the I_K_/I_G_ ratios in GluK1-1a (GluK1-1a: 1.51±0.13, +Neto1: 1.25±0.04 p=0.1500, +Neto2: 1.0±0.07 **p=0.0096), they increased it for the non-spliced variant (GluK1-2a: 0.56±0.04, +Neto1: 1.37±0.12 ****p<0.0001, +Neto2: 1.16±0.04 ****p<0.0001) (Figure 3D; Figure 3—figure supplement 1B; Table 1, Zhang et al., 2009) highlighting the effect of splice residues. Interestingly, this differential modulation of the two variants by Neto proteins resulted in comparable I_K_/I_G_ ratios.

Next, we investigated the effects of Neto1 and Neto2 on the voltage-dependent endogenous polyamine block since the presence of splice insert had enhanced the outward rectification of GluK1. We observed that both Neto1 and Neto2 significantly enhanced the outward rectification of GluK1-2a (GluK1-2a: 0.61±0.10, +Neto1: 1.14±0.14 *p=0.0338, +Neto2: 1.37±0.10 **p=0.0022). However, they did not significantly increase it for GluK1-1a (GluK1-1a: 0.96±0.11, +Neto1: 1.16±0.09 p=0.1880, +Neto2: 0.80±0.05 p=0.2401) and did not show differential modulation (Figure 3E and F; Table 1*)*.

We also calculated desensitization and deactivation kinetics in excised outside-out patches. However, GluK1-1a receptors, when expressed alone, exhibited low peak amplitudes in outside-out recordings that prevented reliable calculation of gating kinetics. Hence, we only compared the properties of receptors co-expressed with Neto proteins, and the results were consistent with those obtained from whole-cell recordings (Figure 3G-H, Table 2). Neto 2 slowed down desensitization of GluK1-1a by ~1.5 times compared to GluK1-2a (Ʈ_Des_, GluK1-1a +Neto2: 31.89±4.08 ms; GluK1-2a +Neto2: 20.91±2.11; p=0.0665) (Figure 3G, Table 2). The desensitization rates for GluK1-1a and GluK1-2a receptors co-expressed with Neto1 was also significantly altered (Ʈ_Des_, GluK1-1a +Neto1: 4.83±0.46 ms; GluK1-2a +Neto2: 2.84±0.35; *p=0.0310) (Figure 3G, Table 2). Faster solution exchange times in excised patch recordings also allowed us to measure deactivation kinetics using a 1ms application of 10 mM glutamate. Surprisingly, unlike desensitization, for receptors coexpressed with Neto2, the deactivation rate of GluK1-1a is significantly faster compared to that of GluK1-2a (Ʈ_Dea_, GluK1-1a +Neto2: 5.18±0.65 ms; GluK1-2a +Neto2: 10.74±1.48; **p=0.0077). In contrast, Neto1 did not significantly alter deactivation kinetics of both GluK1 variant receptors (Ʈ_Dea_, GluK1-1a +Neto1: 2.83±0.20 ms; GluK1-2a +Neto1: 2.14±0.37; p=0.2086) (Figure 3H, Table 2). Thus, both whole-cell and excised patch recordings confirm the unique functional properties and differential modulation by Neto proteins due to the presence of fifteen amino acid inserts in GluK1-1a receptors.

**Table 2.** Excised patch outside-out electrophysiology of GluK1-1a and GluK1-2a in the absence or presence of Neto1 (green) or Neto2 (peach). Errors are reported as SEM. Statistical-significance is reported at 95% CI. p<0.05 (*), p<0.01 (**), p<0.001 (***), p<0.0001 (****) for comparisons between GluK1-1a and GluK1-2a receptors in presence of Neto proteins. Red p-values are statistical significance at 95% for comparison between GluK1-1a and GluK1-2a with either of the Neto proteins.

Name of the construct	Deactivation		Desensitization		Total No. of cells tested
	Ʈ_Deact_ (ms)	Rise Time (ms)	Ʈ_Des_ (ms)	Rise Time (ms)	
GluK1-1a	Low peak amplitude	0.68±0.19 (N=3)	Low peak amplitude	0.48±0.1 (N=3)	10
+Neto1	2.83±0.2 (N=9)	0.75±0.04 (N=9) (p=0.7615)	4.83±0.46 (N=9)	0.71±0.04 (N=9)	9
+Neto2	5.18±0.65 (N=3)	1.5±0.24 (N=3) (p=0.0627)	31.89±4.08 (N=4)	1.87±0.06 (N=4)	4
GluK1-2a	1.51±0.28 (N=4)	0.58±0.06 (N=4)	2.34±0.35 (N=5)	0.72±0.07 (N=5)	9
+Neto1	2.14±0.37 (N=10) (p=0.2086)	0.75±0.06 (N=10) (p=0.0731)	2.84±0.69 (N=9) (p=0.5312 / *p=0.0310)	0.69±0.07 (N=9)	12
+Neto2	10.74±1.48 (N=8) (***p=0.0004 / **p=0.0077)	1.47±0.23 (N=8) (p=0062)	20.91±2.11 (N=9) (***p=0.0003 / p=0.0665)	1.42±0.25 (N=9)	9

### Mutations in GluK1-1a splice insert alter channel properties

**Figure 4.**
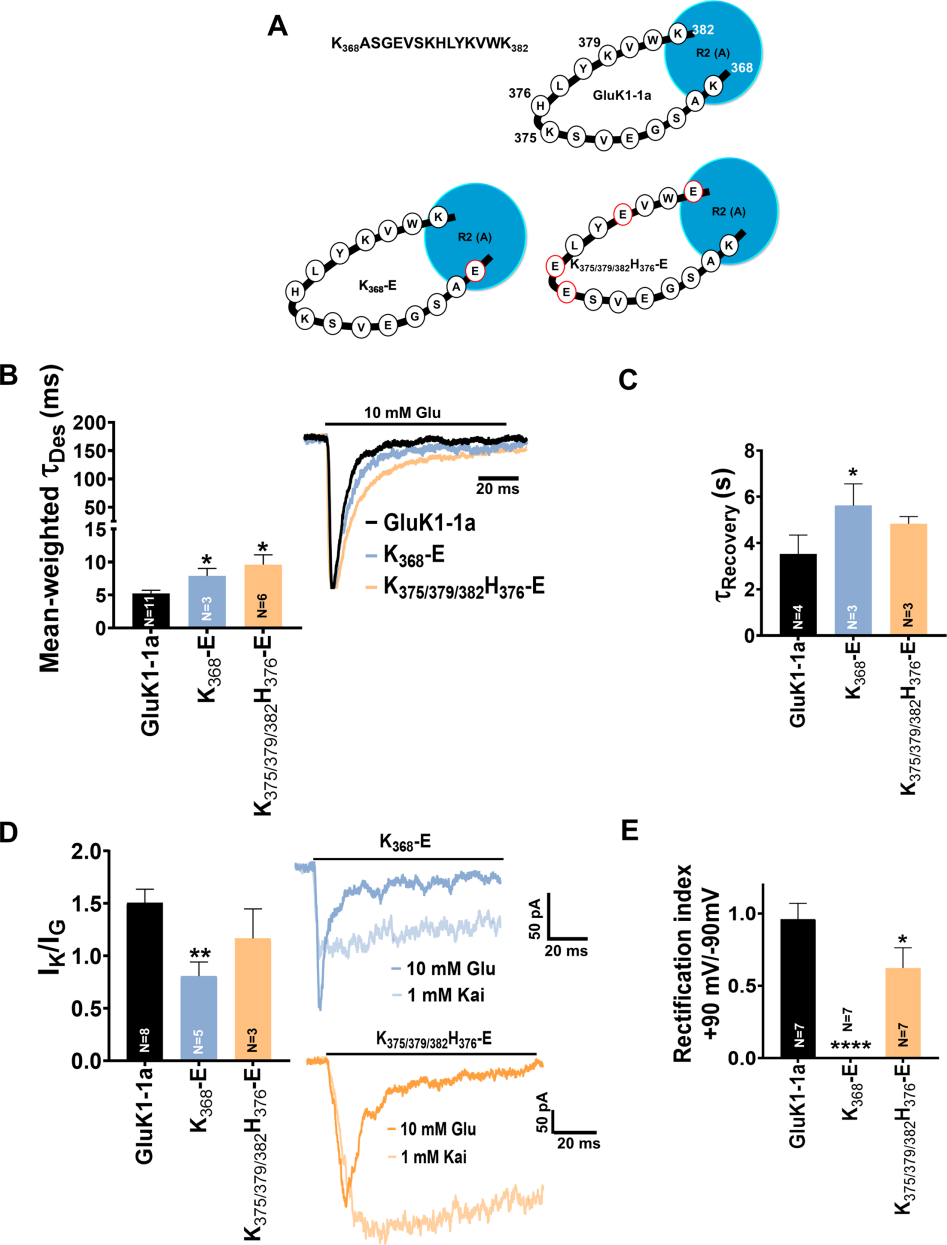
Mutation of GluK1-1a splice insert residues affects the desensitization and recovery kinetics of the receptor. Bar graphs (mean ± SEM) show a comparison between wild-type and mutant receptors for different kinetic properties. (**A**) Schematic representation of 15 residues amino-terminal domain (ATD) splice (K_368_ASGEVSKHLYKVWK_382_) in wild-type and mutant receptors under study (**B**) Mean-weighted Tau (τ_Des_) values for GluK1-1a wild-type and mutant receptors in the presence of 10 mM glutamate. (**C**) Tau (τ_Recovery_) recovery values for GluK1-1a and mutants. (**D**) The ratio of the peak amplitudes evoked in the presence of 1 mM kainate and 10 mM glutamate is shown for GluK1-1a mutants.(**E**) The rectification index represented by the ratio of currents evoked by 10 mM glutamate application at +90 mV and –90 mV for the wild-type and mutant receptors is shown. The wild-type GluK1 splice variant data is the same as from Figure 2A and is replotted here for comparison. Error bars indicate mean ± SEM, N in each bar represents the number of cells used for analysis, and * indicates the significance at a 95% confidence interval. Figure 4—source data 1.Data used for the electrophysiology plots. Figure 4—source data 2.GluK1-1a amino-terminal domain (ATD) splice mutants.The various mutants used in the study and the primer sequences to make charge-neutral and charge-reversal mutants in GluK1-1a are tabulated.

Since our electrophysiological analysis showed functional differences between the two splice variants and their modulation by Neto proteins, we created receptors with mutated splice residues and conducted functional assays to identify the key residues. The splice insert (KASGEVSKHLYKVWK) is dominated by positively charged residues and contains four lysines and one histidine. We hypothesized that these charged residues might affect the interactions at the ATD-LBD interface and influence receptor functions. To investigate this, we prepared charge-reversal (K/H to E) and charge-neutral (K/H to A) mutants and carried out functional assays (Figure 4—source data 2). Our cell surface biotinylation assay showed that all mutants (glutamate or alanine) reached the cell surface efficiently (data not shown). However, the charge-neutral mutants (K/H to A) gave either very low peak amplitudes (<40 pA) or were not functional and hence, were not included in the study. The charge reversal mutants K_368_-E and K_375/379/382_H_376_-E revealed fascinating insights into the role of splice residues in altering GluK1 receptor properties (Figure 4A). Interestingly, both mutants, K_368_-E and K_375/379/382_H_376_-E exhibited a significantly slower rate of glutamate-evoked desensitization compared to wild-type GluK1-1a (Ʈ_Des_, GluK1-1a: 5.21±0.50 ms; K_368_-E: 7.89±1.14 ms *p=0.0334; K_375/379/382_H_376_-E: 9.62±1.47 *p=0.0290) (Figure 4B; Table 1). We also observed a significant delay in the recovery from the desensitized state for K_368_-E mutant (K_368_-E: 5.61±0.95 s *p=0.0417) compared to wild-type GluK1-1a. In addition, the K_375/379/382_H_376_-E mutant also exhibited a slowdown in the recovery, though not significant. (K_375/379/382_H_376_-E: 4.83±0.31 s p=0.2774) (Figure 4C; Table 1). Our investigations of glutamate- and kainate-evoked responses for wild-type and mutant receptors considering their peak amplitudes (I_K_/I_G_) revealed a significant decrease for the K_368_-E mutant (GluK1-1a: 1.51±0.13; K_368_-E: 0.81±0.13 **p=0.0037) and a reduction was observed for K_375/379/382_H_376_-E receptors (1.17±0.28 p=0.3733) compared to wild-type although the differences do not reach statistical significance (Figure 4D; Table 1). These observations are reciprocal to the effect of splice insert compared to the non-spliced form, indicating the importance of these residues in influencing receptor desensitization and recovery. A similar trend reversal was also observed for the measurements of the rectification index for these mutants at positive and negative potentials (+90 mV and –90 mV). The rectification index was significantly reduced in the case of mutant K_375/379/382_H_376_-E (K_375/379/382_H_376_-E: 0.62±0.14 *p=0.0499). Surprisingly, no outward rectification was observed for the K_368_-E mutant, and further investigation is needed to fully understand the reasons for the same (Figure 4E; Figure 5—figure supplement 2C; Table 1).

### GluK1-1a splice residues K_368,_ K_375_, H_376_, K_379_, and K_382_ influence receptor modulation by Neto proteins

Next, we investigated the effects of splice mutants on receptor modulation by Neto proteins. To test whether mutations in splice residues could disrupt interactions with Neto proteins, we performed receptor pull-downs using an antibody against the His-tag of the receptor. Our results showed that the mutant receptors could efficiently pull down Neto1 (detected using the Neto1 antibody) and Neto2-EGFP (detected using the GFP antibody) (Figure 5—figure supplement 1), suggesting that mutants don’t altogether abolish GluK1-1a and Neto interactions.

**Figure 5.**
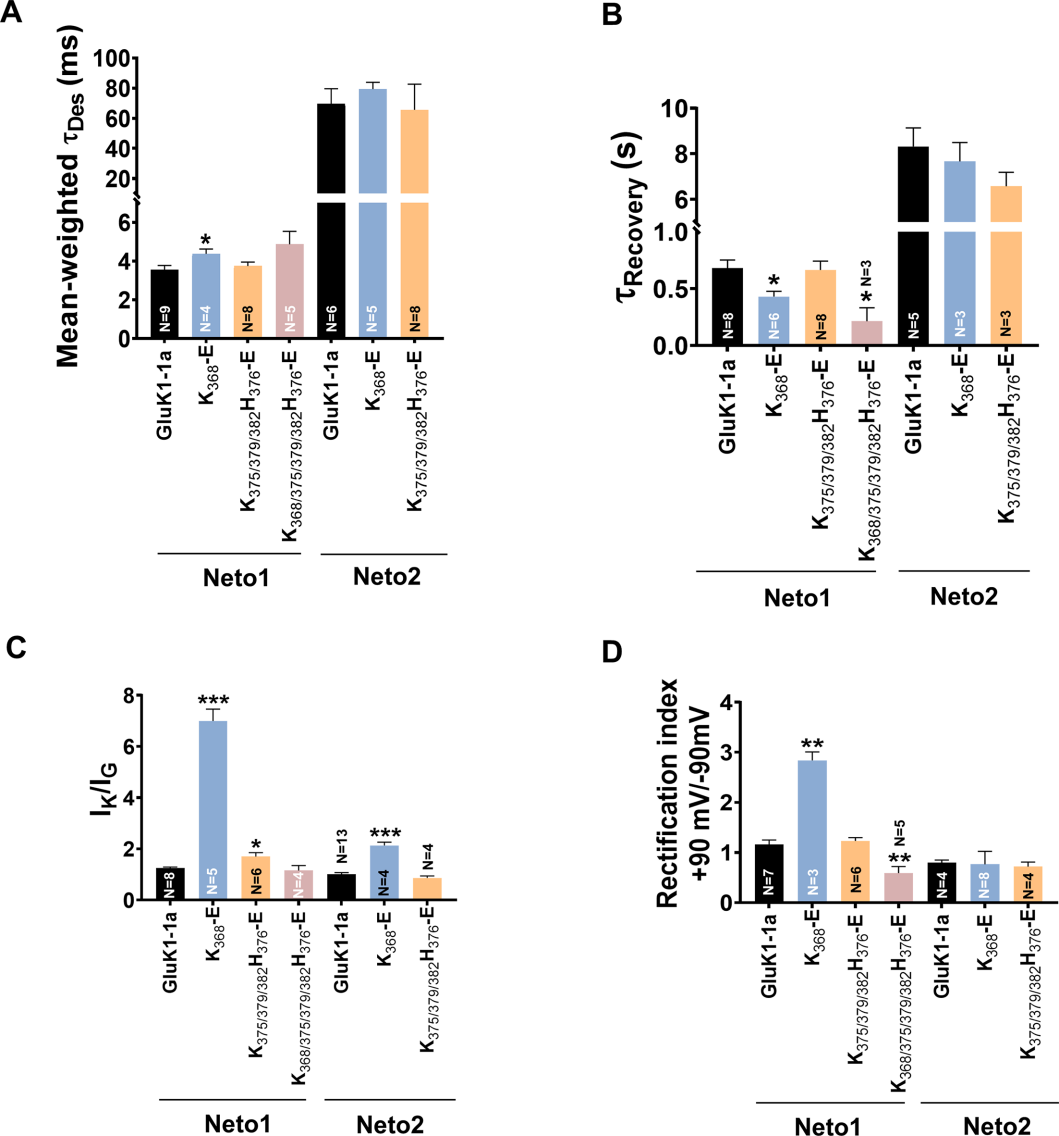
Mutation of GluK1-1a splice insert residues affects the receptor modulation by Neto proteins. Bar graphs (mean ± SEM) show a comparison between wild-type and mutant receptors with Neto proteins for different kinetic properties. (**A**) Mean-weighted Tau (τ_Des_) values for GluK1-1a wild-type and mutant receptors in the presence of 10 mM glutamate and expressed with Neto1/2. (**B**) Tau (τ_Recovery_) recovery values for GluK1-1a and mutants with Neto1/2. (**C**) The ratio of the peak amplitudes evoked in the presence of 1 mM kainate and 10 mM glutamate for GluK1-1a mutants co-expressed with Neto1/2 is shown. (**D**) The rectification index represented by the ratio of currents evoked by 10 mM glutamate application at +90 mV and –90 mV for the wild-type and mutant receptors with Neto proteins is shown. The wild-type GluK1 splice variants’ data is the same as in Figure 2 and is replotted here for comparison. Error bars indicate mean ± SEM, N in each bar represents the number of cells used for analysis, and * indicates the significance at a 95% confidence interval. Figure 5—source data 1.Data used for the electrophysiology plots.

Furthermore, we conducted electrophysiology experiments to investigate whether coexpression of Neto proteins can restore functionality and influence the functions of splice mutants. We observed that mutant K_368_-E desensitizes significantly slower in the presence of Neto1 while the mutants K_375/379/382_H_376_-E and K_368/375/379/382_H_376_-E do not exhibit any significant deviation (Ʈ_Des_, GluK1-1a +Neto1: 3.56±0.22 ms; K_368_-E +Neto1: 4.37±0.25 ms *p=0.0393; K_375/379/382_H_376_-E +Neto1: 3.74±0.20 ms p=0.5390; K_368/375/379/382_H_376_-E +Neto1: 4.86±0.67 ms p=0.1242) (Figure 5A; Figure 5—figure supplement 2A; Table 1). This observation suggests that some splice residues influence GluK1-1a modulation by Neto1, and more mutational, functional, and structural studies on this interaction are necessary. On the other hand, Neto2 does not significantly affect the desensitization of these mutant receptors compared to wild-type-Neto2 (Figure 5A; Figure 5—figure supplement 2A; Table 1*)*.

**Figure 5—figure supplement 1.**
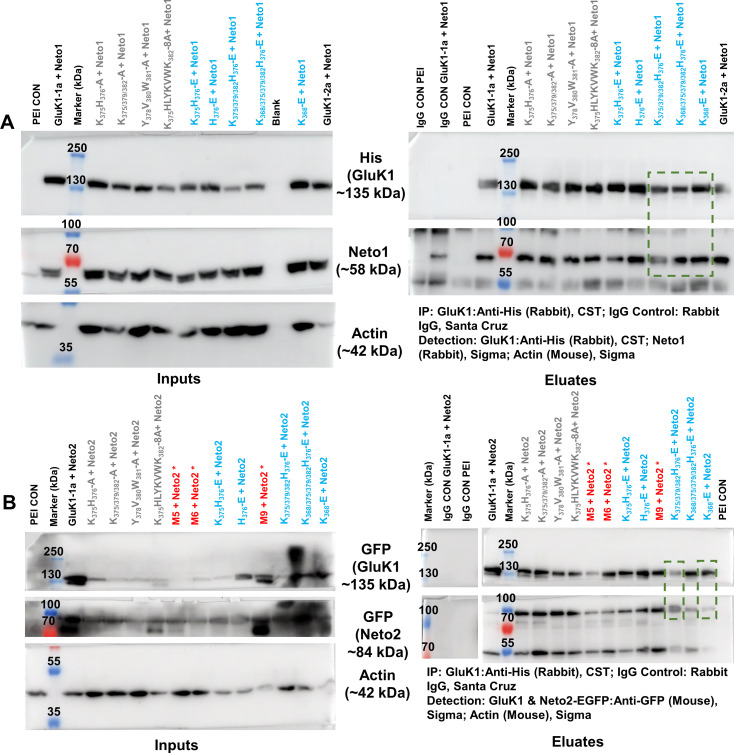
Co-immunoprecipitation analysis of GluK1-1a splice mutants and Neto proteins. (A, B) represent the raw western blots for receptor pull-down experiments using His-antibody (CST). The left and right panels show the inputs and eluates for the co-IP. Actin was used as an internal control. The antibodies used to detect receptor or Neto proteins have been indicated. Rabbit IgG controls were set up using WT receptor with Neto1 or Neto2 and negative control along with the test samples to check for non-specific interactions. All the experiments were done in triplicates. Charge-neutral (Ala) mutants are labeled in gray, charge reversal (Glu) mutants in cyan and marker, and controls in black. Red asterisks are mutants not used for further studies. The molecular weight of markers and the expected proteins have been indicated. Green boxes display that the mutant receptors shown in the functional analysis were able to interact with both Neto proteins efficiently when co-transfected with Neto1 or Neto2. The western blots are raw images without any editing, they are assembled as they were cut from the same blot for probing with respective primary and secondary antibodies as mentioned in the panels A and B. Figure 5—figure supplement 1—source data 1.PDF files containing labelled western blots for Figure 5—figure supplement 1A. Figure 5—figure supplement 1—source data 2.Original files for western blots displayed in Figure 5—figure supplement 1A. Figure 5—figure supplement 1—source data 3.PDF files containing labelled western blots for Figure 5—figure supplement 1B. Figure 5—figure supplement 1—source data 4.Original files for western blots displayed in Figure 5—figure supplement 1B.

**Figure 5—figure supplement 2.**
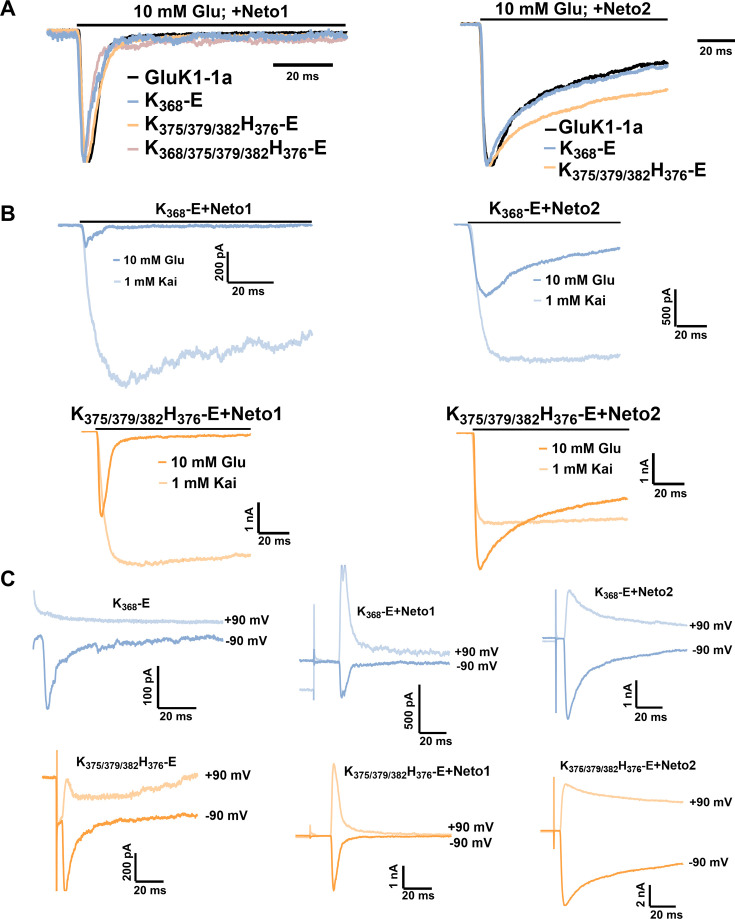
Representative traces for the GluK1 mutant receptors with Neto proteins. (**A**) Demonstrates the normalized traces for wild-type and mutant receptors with Neto1/2 for glutamate-evoked desensitization. (**B**) Displays representative traces for glutamate vs. kainate evoked responses in the presence of Neto1 or Neto2. (**C**) Representative 100 ms traces for currents evoked at positive (+90 mV) and negative (–90 mV) potentials in the absence and presence of Neto proteins. Figure 5—figure supplement 2—source data 1.Data used for the electrophysiology plots.

Similarly, K_368_-E and K_368/375/379/382_H_376_-E mutants recover significantly faster from the desensitized state when coexpressed with Neto1. On the other hand, the recovery from the desensitized state for mutant receptors is not significantly affected by Neto2. Thus, while Neto1 seems to affect the mutant receptor recovery from the desensitized state, Neto 2 doesn’t show significant differences compared to wild-type receptors, again highlighting the differential modulation of GluK1-1a receptors by Neto proteins (Figure 5B; Table 1*)*.

Furthermore, to determine whether the agonist efficacy of mutant receptors changed in the presence of Neto proteins, I_K_/I_G_ ratios were measured. Neto1 increased the agonist efficacy for the K_368_-E and K_375/379/382_H_376_-E mutants but not for K_368/375/379/382_H_376_-E receptors (K_368_-E +Neto1: 6.99±0.47 ***p=0.0002; K_375/379/382_H_376_-E +Neto1: 1.69±0.15 *p=0.0326; K_368/375/379/382_H_376_-E +Neto1: 1.15±0.20 p=0.6495) (Figure 5C; Figure 5—figure supplement 2B; Table 1). Similarly, Neto2 also considerably increased the I_K_/I_G_ values for mutant K_368_-E, rescuing the kainate efficacy (K_368_-E +Neto2: 2.13±0.13 ***p=0.0007), while it does not seem to have any significant effect on the K_375/379/382_H_376_-E mutant (K_375/379/382_H_376_-E +Neto2: 0.86±0.08 p=0.2045) (Figure 5C; Figure 5—figure supplement 2B; Table 1).

Consistent with the observation above, our examination of the rectification index revealed a significant increase in outward current for the K_368_-E mutant (K_368_-E +Neto1: 2.84±0.16 **p=0.0021). However, K_375/379/382_H_376_-E did not show any difference compared to wild-type receptors, while K_368/375/379/382_H_376_-E displayed a significant decrease in the outward current (K_375/379/382_H_376_-E +Neto1: 1.23±0.07 p=0.5351; K_368/375/379/382_H_376_-E+Neto1: 0.59±0.13 **p=0.0081) (Figure 5D; Figure 5—figure supplement 2C; Table 1). Interestingly, however, the splice residue mutants did not show any significant variation in the rectification index when coexpressed with Neto2 (Figure 5D; Figure 5—figure supplement 2C; Table 1).

Thus, our analysis of the gating properties of GluK1-1a mutants co-expressed with Neto proteins suggests that positively charged residues at positions 368, 375, 376, 379, and 382 in the splice insert influence receptor modulation by Neto proteins. Neto1 appears to have more pronounced effects on the mutant receptors compared to Neto2. Specifically, Neto1 significantly slowed desensitization for the K_368_-E mutant, accelerated recovery from desensitization for K_368_-E and K_368/375/379/382_H_376_-E mutants, increased agonist efficacy for K_368_-E and K_375/379/382_H_376_-E mutants, and altered rectification properties for K_368_-E and K_368/375/379/382_H_376_-E mutants. In contrast, Neto2 had fewer significant effects on the mutant receptors, with the main impact being an increase in agonist efficacy for the K_368_-E mutant. Notably, Neto2 did not significantly affect desensitization, recovery from desensitization, or rectification properties of the mutant receptors when compared with wild-type GluK1-1a coexpressed with Neto2. These findings suggest that the splice residues in GluK1-1a differentially influence receptor modulation by Neto1 and Neto2, with Neto1 showing more extensive modulation of the mutant receptors' functional properties.

### The structure of GluK1-1a_EM_ shows an overall conserved architecture of the desensitized state in kainate receptors

**Figure 6.**
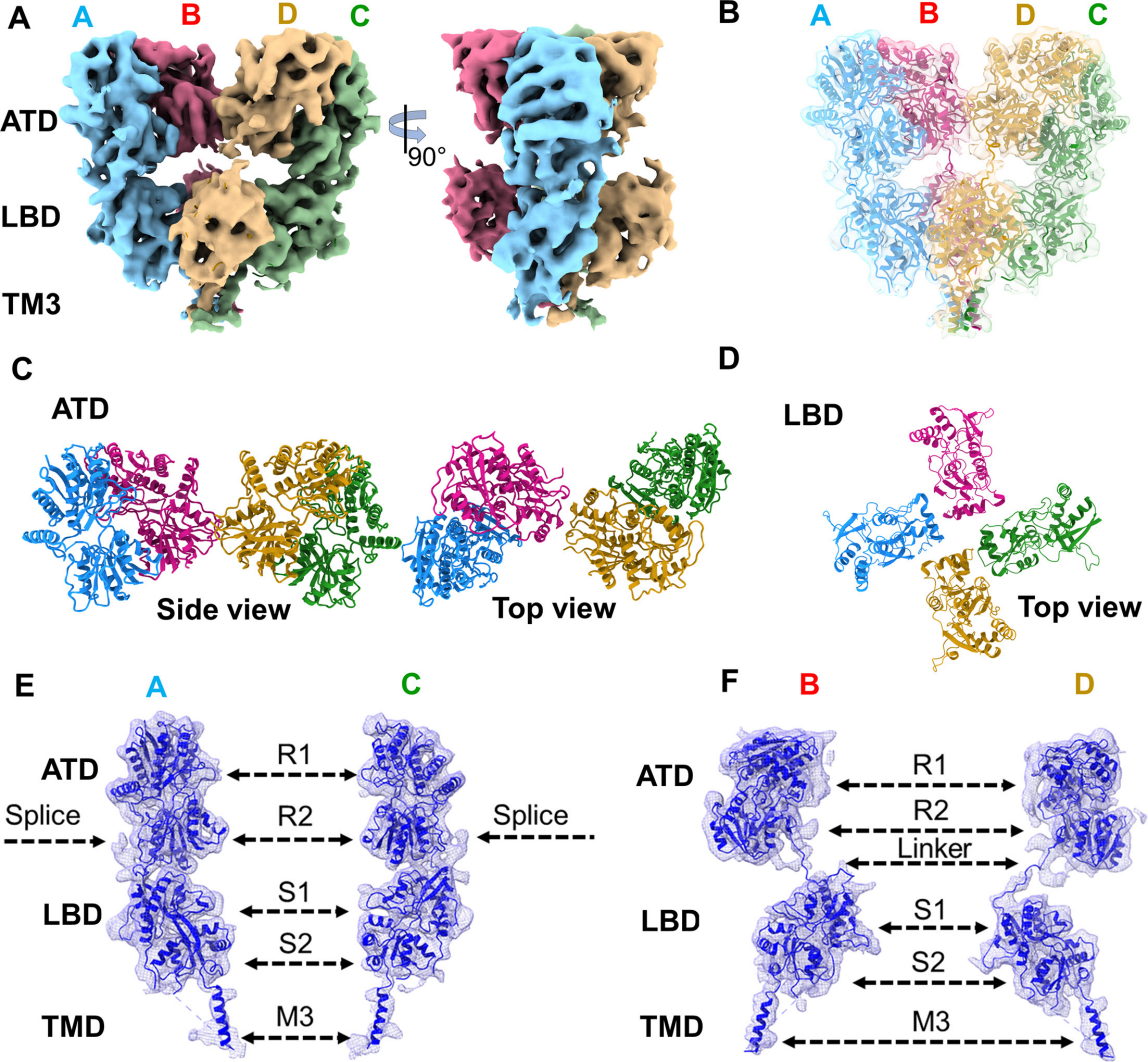
Architecture of GluK1-1a_EM_ reconstituted in nanodisc for SYM-bound desensitized state. (**A**) Shows the segmented density map colored according to unique chains of the receptor tetramer (A- blue, B-pink, C-green, and D-gold) at 5.23 Å in side view and 90° rotated orientations. (**B**) Shows the final model fitted in the EM map. (**C** and **D**) Top views of amino-terminal domain (ATD) and ligand binding domain (LBD) layers. (**E** & **F**) Display the segmented map fitted with the corresponding distal (A & C) and proximal (B & D) chains. Receptor sub-domains, the position of splice insertion, and linkers are indicated.

To evaluate the effects of splice residues on domain organization and structure of GluK1-1a receptors, we pursued its structure determination *via* single particle cryo-EM. Construct optimization was carried out to improve the expression and stability of purified protein. Briefly, the free cysteines in the TM1 region were mutated (C_552_Y, C_557_V) based on the sequence analysis with kainate and AMPA receptors, and this construct was named GluK1-1a_EM_(Figure 6, Figure 6—figure supplements 1 and 2). The whole-cell patch clamp showed that GluK1-1a_EM_ was functional (Figure 6—figure supplement 3).

**Figure 6—figure supplement 1.**
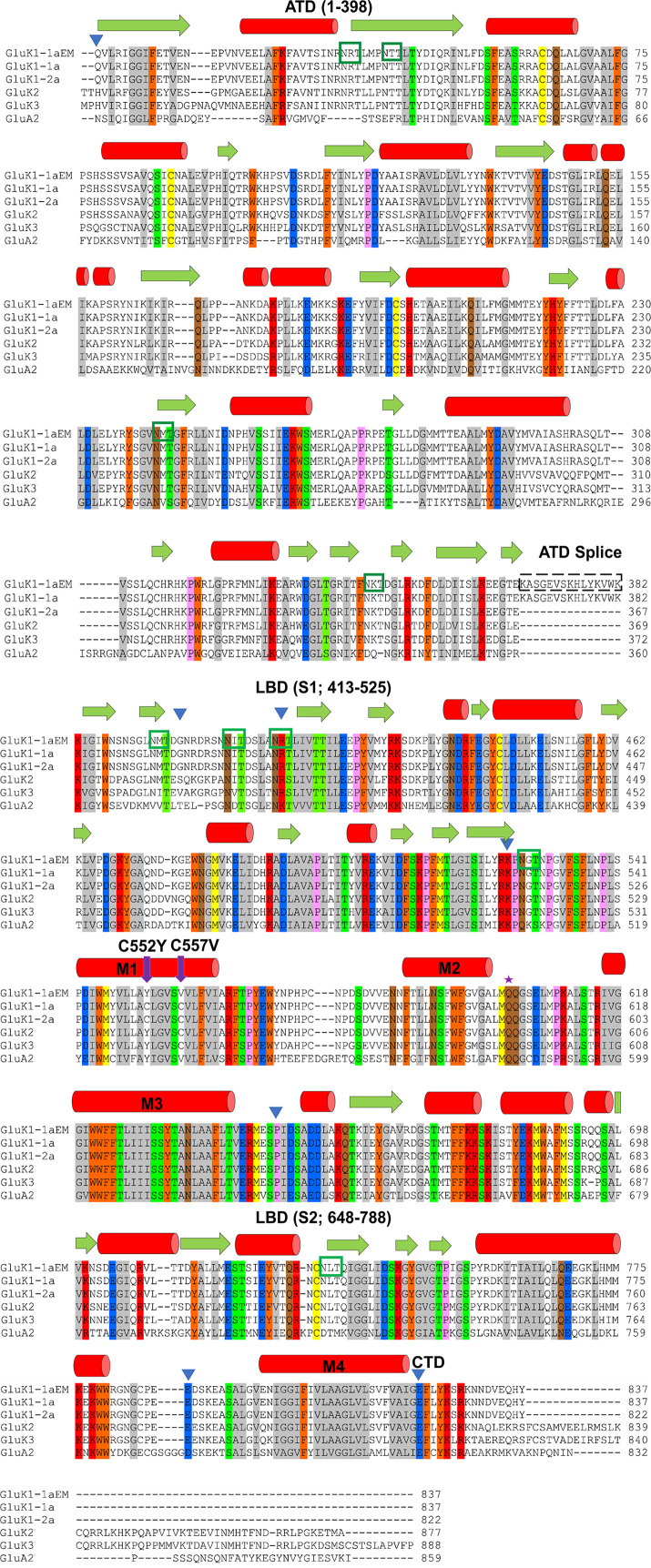
Sequence alignment and construct design of GluK1-1a_EM_. The EM construct was aligned with mature polypeptide sequences of wild-type (WT) rat GluK1-1a, GluK1-2a, GluK2, GluK3, and GluA2. The color scheme grouping was based on the similarity of residues, with gray (**G, A, V, L, I**), orange (**F, Y, W**), yellow (**C, M**), green (**S, T**), red (**K, R, H**), blue (**D, E**), brown (**N, Q**), and pink (**P**). The approximate domain boundaries and the residue numbers have been marked with blue inverted triangles and written in parenthesis. Green boxes mark predicted N-linked glycosylation sites (NXT). Purple arrows show cysteine mutations in the EM construct. The predicted secondary structure is shown above the sequence, with red cylinders for the α-helix and green arrows for the β-strand.

**Figure 6—figure supplement 2.**
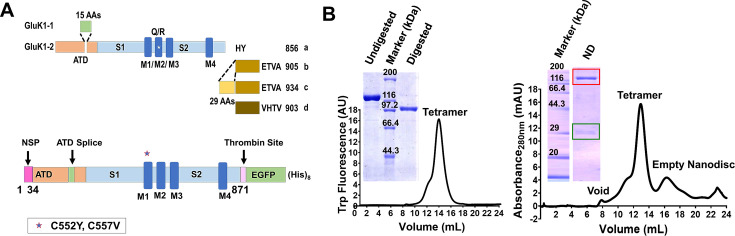
GluK1-1a_EM_ construct design and purification. (**A**) Schematic representation of GluK1 splice variants present in the human brain along with a schematic for the GluK1-1a_EM_ construct is shown. The N-terminal Signal Peptide (NSP; 1–34 residues), 15 amino acid splice insert in the amino-terminal domain (ATD), S1/S2 (ligand binding domain, LBD), M1-M4 (trans-membrane domain, TMD) and C-terminal thrombin site followed by EGFP and (His)_8_ tags. The star in the M1 region denotes the point mutation of free cysteines at positions 552 and 557; the numbering of residues is based on the mature polypeptide. (**B**) Superose 6 size exclusion chromatography profiles for the purified protein in detergent micelles and nanodisc, respectively, are shown, and the position corresponding to void, receptor tetramer, and empty nanodisc are indicated. SDS-PAGE gel images inset shows the undigested and thrombin-digested protein for GluK1-1a_EM_ in detergent and MSP1E3D1 (green box) co-eluting with GluK1-1a_EM_ (red box), revealing a stable protein-nanodisc complex, respectively. ND indicates receptors in lipid nanodiscs. Figure 6—figure supplement 2—source data 1.Size exclusion chromatography data used for the plots. Figure 6—figure supplement 2—source data 2.PDF files containing original SDS-PAGE gels for Figure 6—figure supplement 2B -inset 1 with rectangle indicating the cropping margin. Figure 6—figure supplement 2—source data 3.Original uncropped SDS-PAGE gels for Figure 6—figure supplement 2B -inset 1. Figure 6—figure supplement 2—source data 4.PDF files containing original SDS-PAGE gels for Figure 6—figure supplement 2B -inset 2 with rectangle indicating the cropping margin. Figure 6—figure supplement 2—source data 5.Original uncropped SDS-PAGE gels for Figure 6—figure supplement 2B -inset 2.

**Figure 6—figure supplement 3.**
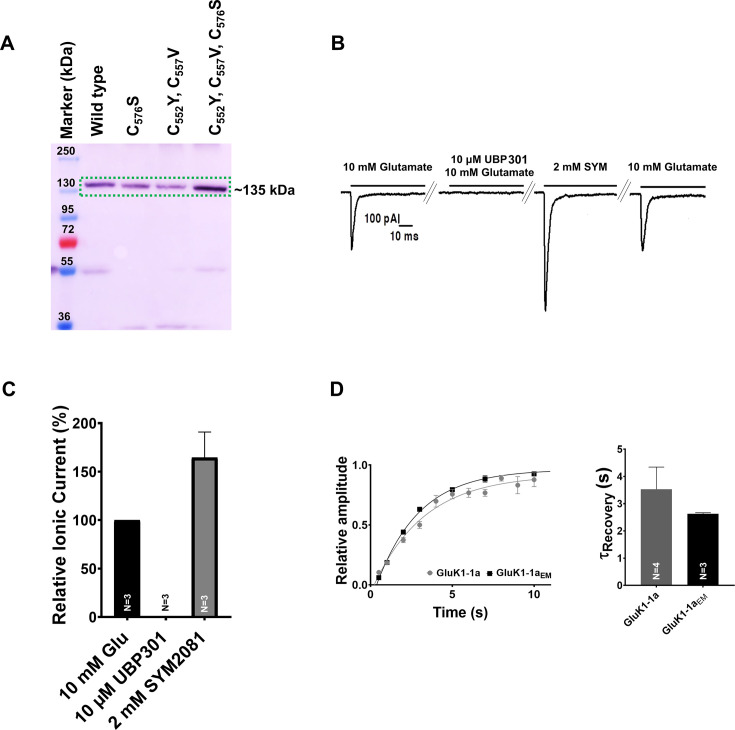
GluK1-1a construct optimization for structural studies and its gating properties. The optimized constructs were verified for expression. Here, three free cysteines mutations in the TM1 are represented as C_576_S (1 x Cys), C_552_Y, C_557_V (2 x Cys), and C_552_Y, C_557_V, C_576_S (3 x Cys). 2 x Cys (C_552_Y, C_557_V) mutant was used as GluK1-1a_EM_ for structural studies. (**A**) Western blot probed with anti-His antibody (Cell Signaling Technology, USA) shows the expression of all the mutants with respect to the wild-type (WT) construct; the Expected size of the polypeptide is indicated (MW:~135 kDa). (**B**) Representative traces for whole-cell patch clamp recordings (36–48 hr post-infection) of HEK293 cells infected with GluK1-1a_EM_ baculovirus is shown. The receptors showed activation by 10 mM Glutamate, blocked in the presence of the inhibitor 10 µM UBP301. Post-washing the reversible inhibitor, the receptor could undergo a similar activation and desensitization cycle in the presence of 2 mM SYM and 10 mM Glutamate. (**C**) Shows the graphical representation of the percentage of relative current in the presence of 10 µM UBP301, 2 mM SYM, and 10 mM glutamate. (**D**) Electrophysiology profiles confirm that the GluK1-1a_EM_ construct behaves similarly to the wild-type GluK1-1a in terms of recovery from desensitization. Error bars indicate mean ± SEM, and N in each bar represents the number of cells used for analysis. Figure 6—figure supplement 3—source data 1.Uncropped western blot with rectangle indicating the cropping margin. Figure 6—figure supplement 3—source data 2.Original file for western blot displayed in Figure 6—figure supplement 3A. Figure 6—figure supplement 3—source data 3.Data used for the electrophysiology plots in panels B-D.

**Figure 6—figure supplement 4.**
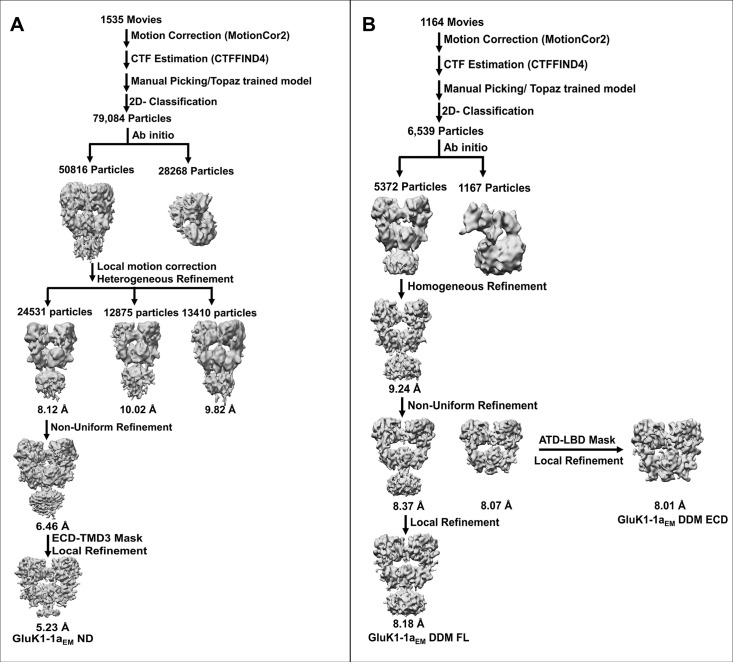
Single-particle cryo-EM data processing flow chart for GluK1-1a_EM_ in nanodisc (ND) and detergent (DDM). (**A**) For the GluK1-1a_EM_ ND dataset, 79,084 particles from the final 2D classification were used for initial 3D reconstruction into two classes to remove junk particles. Furthermore, the good 50,816 particles were polished using local motion correction, and the initial 3D map was heterogeneously refined into three classes. The best map (24531 particles, highlighted in red box) was subsequently refined using non-uniform refinement followed by local refinement using the ECD-TMD3 mask to attain the final density map (GluK1-1a_EM_ ND) resolution of 5.23 Å at 0.143 FSC. (**B**) 6539 particles from the final 2D classification were used to determine *ab initio* 3D reconstruction of GluK1-1a_EM_ DDM in 2 classes to remove broken particles. The 3D map was refined using good particles (5372, highlighted in red box) with homogenous refinement to obtain a resolution of 9.2 Å. Further, non-uniform refinement followed by local refinement was performed using full-length and extracellular domain (ECD = ATD + LBD) masks to get final resolutions of 8.2 Å (GluK1-1a_EM_ DDM FL) and 8 Å (GluK1-1a_EM_ DDM ECD), respectively.

**Figure 6—figure supplement 5.**
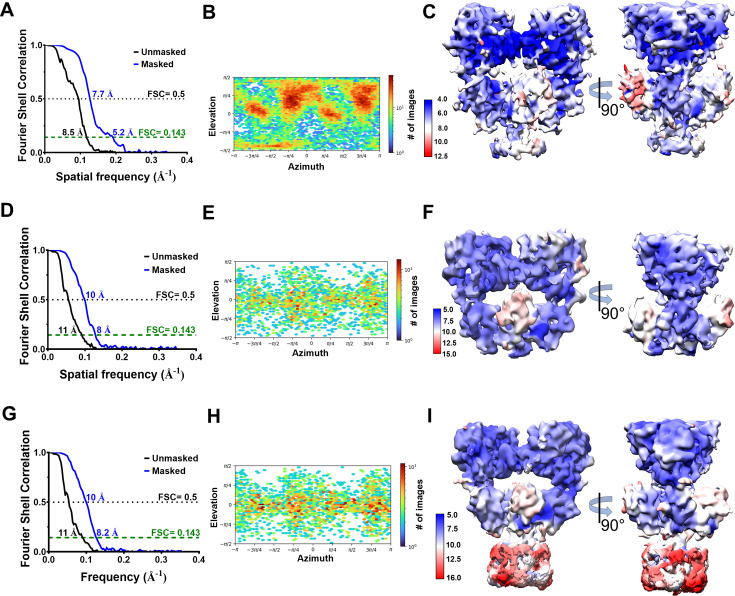
Estimation of resolution and particle distribution for GluK1-1a_EM_ structures. (**A, D, and G**) Show Fourier Shell Correlation curves at 0.143 and 0.5 cut-offs for GluK1-1a_EM_ ND, GluK1-1a_EM_ DDM ECD, and GluK1-1a_EM_ DDM FL reconstructions, respectively, for unmasked (black) and masked (blue) maps estimated in cryoSPARCv3.1. (**B, E, and H**) Show the angular distribution of particles for GluK1-1a_EM_ ND, GluK1-1a_EM_ DDM ECD, and GluK1-1a_EM_ DDM FL maps, respectively, as produced by cryoSPARCv3.1. (**C, F, and I**) Show the local resolution estimates for GluK1-1a_EM_ ND (4–12.5 Å), GluK1-1a_EM_ DDM ECD (5–15 Å), and GluK1-1a_EM_ DDM FL (5–16 Å) maps, respectively. Figure 6—figure supplement 5—source data 1.Data for the FSC plots.

**Figure 6—figure supplement 6.**
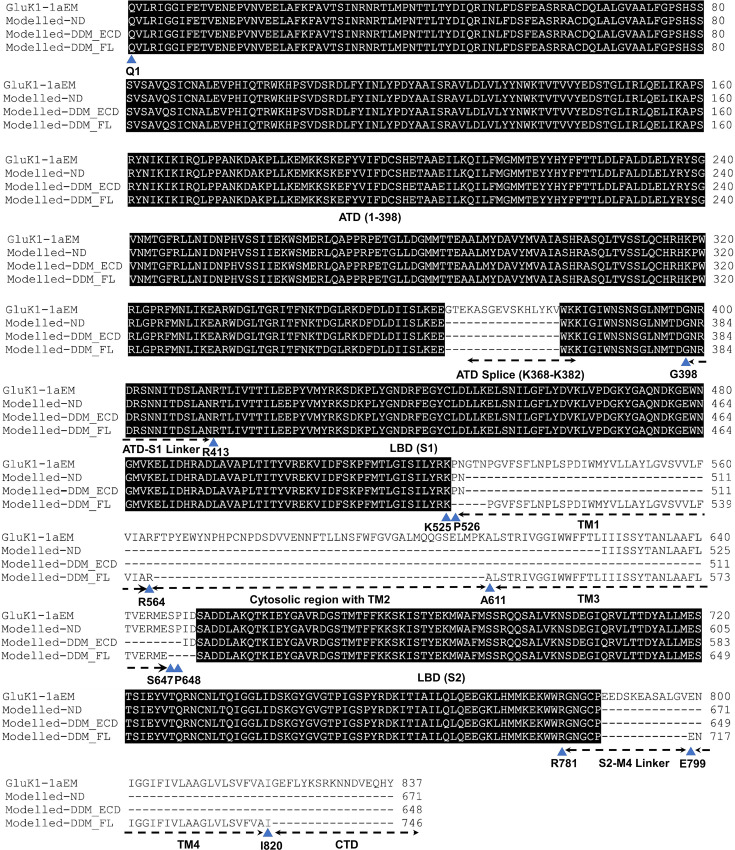
Sequence alignment for the three models presented in the study. Polypeptide chains modeled in the EM maps vs. the full GluK1-1a_EM_ construct are shown. The beginning and ending residues of each modeled domain are indicated by blue triangles for GluK1-1a_EM_ ND, GluK1-1a_EM_ DDM ECD, and FL models. Missing residues are shown as dashed lines that could not be built due to resolution limitations.

**Figure 6—figure supplement 7.**
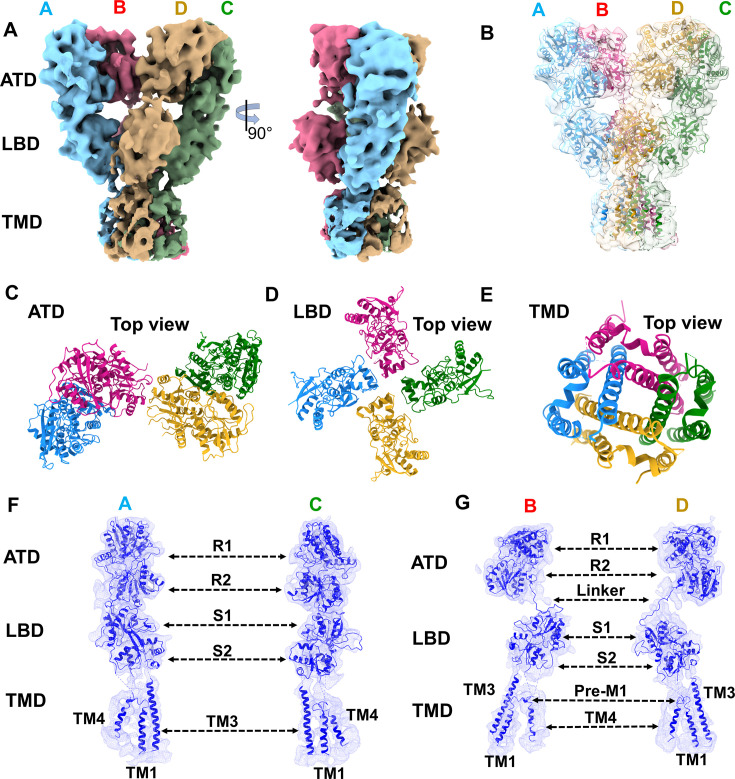
Cryo-EM map and model for GluK1-1a_EM_ dodecyl-β-maltoside (DDM) FL-SYM complex. (**A**) Shows the front and side views of the segmented density map for the DDM solubilized GluK1-1a colored uniquely according to different chains. (**B**) Shows the atomic model fitted in the EM map. (**C, D, and E**) Show the top views of amino-terminal domain (ATD), ligand binding domain (LBD), and TMD layers. (**F, G**) Show the segmented map with fitted chains for each subunit, respectively. Sub-domains and helices of the TMD region are labelled.

**Figure 6—figure supplement 8.**
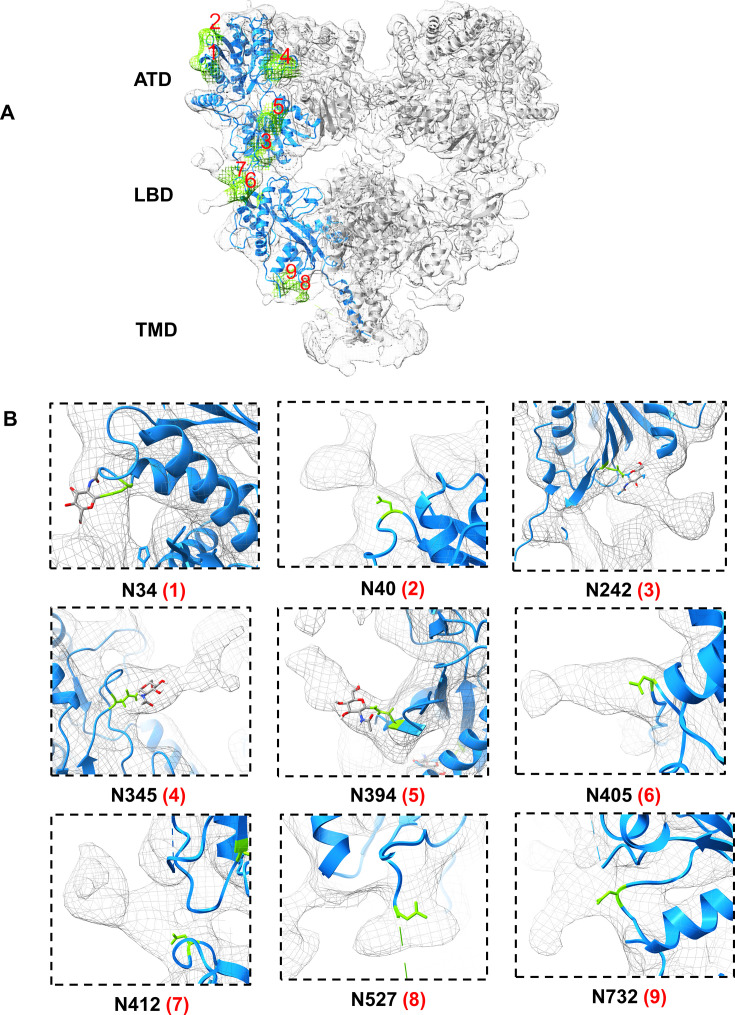
EM density map labeled to show predicted N-linked glycosylation sites (NXT) for GluK1-1a_EM_. (**A**) Shows GluK1-1a_EM_ ND with a fitted model in EM density. Chain A is emphasized in blue, and the residual N-linked glycan densities are shown in green color with respective Asn residues labeled from 1 to 9. (**B**) Shows zoomed view of individual Asn residues with side chain shown as a stick model and the corresponding glycan density in mesh form. For N_34_, N_242_, N_345_, and N_394_ residues, glycan density (NAG) observed in the GluK1-1a ATD crystal structure has been depicted with a conventional color scheme (C- gray, O- red, N- blue, H- white).

**Figure 6—figure supplement 9.**
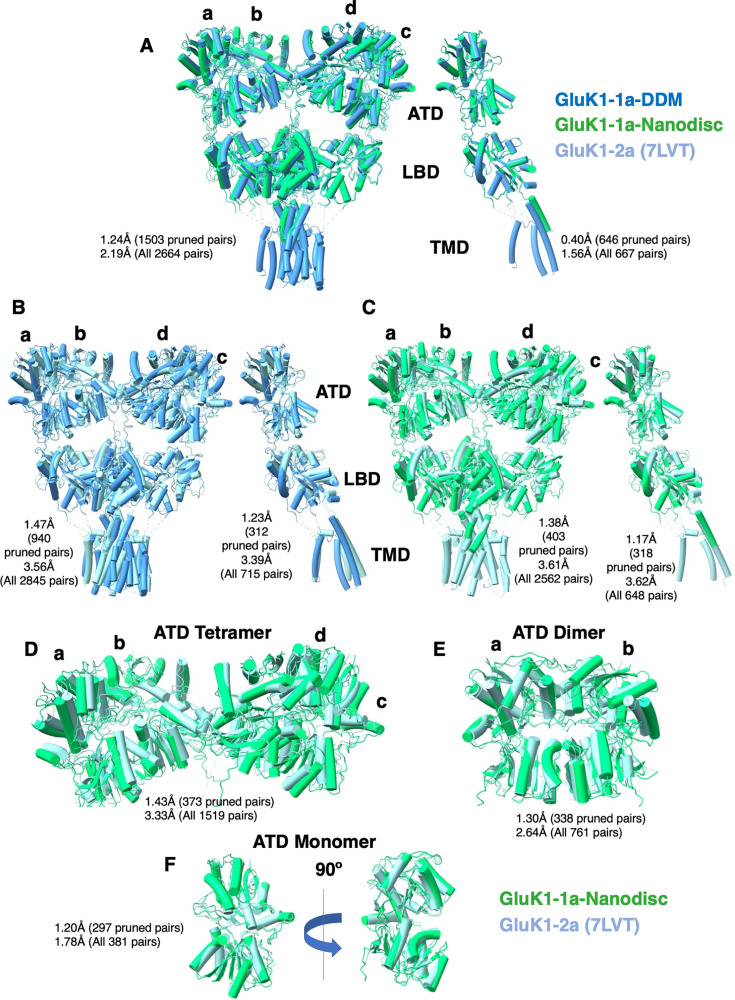
Comparison between GluK1-1a_EM_ (detergent-solubilized or reconstituted in nanodiscs) and GluK1-2a (PDB-7LVT) in the desensitized state. Each panel (**A–F**) illustrates a pairwise comparison with superimposed structures, where the root-mean-square deviation (RMSD) values, measured in Å, are indicated adjacent to each comparison. The structural comparison was carried out in ChimeraX and indicates significant structural similarities between all the protein models. The superimposition does not show significant differences in the arrangement at both amino-terminal domain (ATD) and ligand binding domain (LBD) layers of GluK1-1a with respect to GluK1-2a.

The structures of GluK1-1a_EM_ were determined using single-particle cryo-EM. The receptors were either DDM solubilized or reconstituted in lipid nanodiscs and trapped in a desensitized state using 2 mM of high-affinity agonist 2 S, 4R-4-methyl glutamate (SYM2081) (Figure 6—figure supplement 4). A resolution of ~5.2 Å was achieved for the receptors in lipid nanodiscs, but the transmembrane region was not resolved due to an orientation bias (Figure 6; Figure 6—figure supplement 5; Table 3*)*. The full-length receptor in detergent micelles had a resolution of 8.2 Å, including the transmembrane region, which was ~8 Å for the extracellular domain (Figure 6—figure supplement 5; Table 3*)*.

**Table 3.** Cryo-EM data collection, refinement, and validation for GluK1-1a_EM_.

	GluK1-1a- 2 S,4R-4-methyl glutamate		
	GluK1-1a_EM_ ND	GluK1-1a_EM_ DDM ECD	GluK1-1a_EM_ DDM FL
Data Collection and Processing			
Microscope	Titan Krios	Titan Krios	
Voltage (keV)	300	300	
Number of micrographs	1535	1100	
Camera	K2	Falcon3	
Mode of recording	Super resolution with energy filter (20 eV slit)	Counting	
Exposure time (s)	12	60	
Total dose (e^-^/Å^2^)	40.8	19.5	
Defocus range (µm)	1.8–3.2	2.0–3.2	
Pixel size (Å)	1.41	1.38	
Symmetry	C1	C1	
Initial particle number	1,97,908	13,750	
Final particle number	24531	5372	
Map resolution (Å)	5.23	8.01	8.2
FSC threshold	0.143	0.143	0.143
Refinement (Phenix)			
Initial model used (PDB code)	(ATD), 3 C32 (LBD), 5KUF(TM3)	(ATD), 3 C32 (LBD)	(ATD), 3 C32 (LBD), 5KUF (TMD)
Model resolution (Å)	5.1/7.3	7.2/9.0	7.8/9.1
FSC threshold	0.143/0.5	0.143/0.5	0.143/0.5
Map-to model fit, CC_mask	0.71	0.69	0.73
Model composition			
Non-hydrogen atoms	21556	20880	23616
Protein residues	2684	2596	2948
R.m.s. deviations			
Bond lengths (Å)	0.004	0.003	0.004
Bond angles (°)	0.861	0.796	0.805
Validation			
MolProbity score	1.92	2.03	2.12
Clashscore	15.03	19.06	20.96
Ramachandran plot			
Favored (%)	96.39	96.27	95.59
Allowed (%)	3.53	3.65	4.38
Disallowed (%)	0.08	0.08	0.03
Rotamer outliers (%)	0.3	0.17	0.12
Cß outliers (%)	0.04	0	0.04

A tetrameric receptor model was built based on the crystal structures of GluK1-1a ATD, GluK1 LBD (kainate-bound state; PDB:3C32), and the TM domain based on a highly identical GluK2_EM_ (PDB:5KUF). Our cryo-EM map represented ATD residues-1–398, but the density corresponding to the ATD splice (368-382) was poorly resolved. The ATD-LBD linkers were resolved for all subunits (A to D) in both structures, the S1 and S2 domains were built entirely, and the TMD (TM1, TM3, and TM4) was built only for detergent-solubilized receptors (Figure 6; Figure 6—figure supplements 6 and 7). For receptors reconstituted in lipid nanodiscs, we observed only the TM3 bundle. TM2 was not resolved in either dataset (Figure 6; Figure 6—figure supplement 7). Extra densities were observed in the ECD layer of the GluK1-1a_EM_ ND map, which coincided with potential N-linked glycosylation sites (Figure 6—figure supplement 8). GluK1-1a_EM_ maps showed general conservation of the architecture of kainate receptors captured in the desensitized state (Khanra et al., 2021; Kumari et al., 2019; Meyerson et al., 2016; Selvakumar et al., 2021). Consistent with earlier studies on homomeric and heteromeric KARs in the desensitized state, GluK1-1a exhibited a modular organization with three layers, namely, the twofold symmetric ATD, quasi-fourfold symmetric LBD and fourfold symmetric TMD. The presence of the ATD splice insert in GluK1-1a did not affect the arrangement of the receptor domains in the desensitized state (Figure 6; Figure 6—figure supplement 7). A superimposition of GluK1-1a_EM_ (detergent-solubilized or reconstituted in nanodiscs) and GluK1-2a (PDB:7LVT) showed an overall conservation of the structures in the desensitized state. No significant movements were observed at both the ATD and LBD layers of GluK1-1a with respect to GluK1-2a (Figure 6; Figure 6—figure supplement 9).

## Discussion

Alternative splicing is a well-known mode of protein function modulation in various ion channels, such as transient receptor potential (TRP) (Gracheva et al., 2011; Zhou et al., 2013), big potassium (BK) (Chen et al., 2005), acid-sensing sodium (ASICs) (Bässler et al., 2001), and Shaker K^+^ channels (Hoshi et al., 1991). In the case of iGluRs, the 38 amino acid residue preceding TM4, known as flip and flop isoforms, is a well-characterized module of AMPARs that affects receptor expression and gating kinetics and is involved in various pathophysiological conditions (Park et al., 2016; Sommer et al., 1990; Stine et al., 2001). For kainate receptors, the combination of different subunits (GluK1-GluK5) and post-transcriptional modifications, such as RNA editing and splicing, provides a broader range of pharmacological and gating properties, which can affect synaptic physiology (Kumar et al., 2011; Kumar and Mayer, 2010; Lerma, 2006; Straub et al., 2016). GluK1 is the most diversified subunit of KARs due to multiple post-transcriptional events and has been widely studied as a potential drug target. A recent report highlighted the equivalent presence of GRIK1-1 (exon 9) with respect to GRIK1-2 (lacking exon 9) (Herbrechter et al., 2021). The dominant presence of the GRIK1-1 gene was also reported in retinal Off bipolar cells of ground squirrels (Lindstrom et al., 2014). Interestingly, an N-terminal splice (exon 5) in a similar position has been observed in the GluN1 subunit of NMDA receptors, which is known to influence the interactions at the ATD-LBD interface and, therefore, proton sensitivity and channel kinetics (Regan et al., 2018). However, this is the first report to decipher the role of the N-terminal splice insert in the kainate receptor family.

Our study compared the role of ATD splice in the kinetics of the GluK1-1a to a previously extensively characterized equivalent receptor without the splice region, GluK1-2a (Copits et al., 2011; Fisher, 2015; Fisher and Fisher, 2014; Sommer et al., 1992; Swanson and Heinemann, 1998). We found that the splice insert affects desensitization, recovery from desensitization, glutamate sensitivity, and channel rectification of the GluK1 receptor. Moreover, the splice also had a profound impact on desensitization with kainate, suggesting that the receptor follows different functional pathways for the two agonists. Our results showed a significant deviation from GluK1-2a in kainate- versus glutamate-evoked currents (I_K_/I_G_), implying a role for splice residues in imparting higher efficacy for kainate. This likely explains the enhanced stability of the kainate-bound state in GluK1-1a, leading to slower desensitization compared to GluK1-2a. Our functional assays of GluK1-1a receptors co-expressed with Neto proteins showed that Neto1 facilitates faster recovery from the desensitized state, consistent with previous reports (Copits et al., 2011; Fisher, 2015; Palacios-Filardo et al., 2016). However, we did not observe the fast onset of desensitization previously reported for GluK-2a (Copits et al., 2011; Fisher, 2015). The differences in observations could be due to variations in experimental conditions, such as the constructs and recording conditions used.

Additionally, our results show that Neto2 retards recovery from desensitization for both GluK1 variants, contradicting earlier reports of conflicting recovery rates with Neto2 for GluK1-2a. This indicates that Neto1 and Neto2 may interact with both GluK1 variants in a mutually exclusive manner or that the splice position may regulate the modulation behavior of Neto2 and Neto1. Our comparison of kainate and glutamate efficacies for the two variants also showed differential modulation by Neto proteins. Both Neto1 and Neto2 reduced the I_K_/I_G_ ratios in GluK1-1a, while they significantly increased the I_K_/I_G_ ratios in GluK1-2a, highlighting the impact of the splice insert on receptor response to the agonists (Figure 3D; Figure 3—figure supplement 1B; Table 1).

Our mutational analysis showed that K_368_, a splice insert residue, affects channel kinetics. The charge reversal mutant (K_368_-E) showed significant differences from the wild-type GluK1-1a for all tested properties, indicating a role for K_368_ in protein-protein and/or protein-glycan interactions at the ATD-LBD interface.

Our electrophysiological assays reveal a complex interplay between GluK1 receptor splice variants and Neto auxiliary proteins. While Neto2 demonstrates a more profound impact on wild-type GluK1-1a function compared to GluK1-2a, particularly in slowing desensitization, Neto1’s modulation appears more sensitive to specific mutations in the splice insert. This suggests that Neto2 may have a stronger overall effect on receptor function through potentially allosteric mechanisms, whereas Neto1 might interact more directly with specific splice insert residues. The differential sensitivity to mutations highlights distinct roles for these auxiliary proteins: Neto2 as a powerful modulator of overall receptor function, and Neto1 as having more specific interactions with the splice insert region. This complexity underscores the nuanced nature of these protein interactions and the need for further structural and functional studies to fully elucidate the mechanisms underlying the functional diversity of GluK1 receptors.

Although our cryo-EM map poorly resolved the splice region, its position can be ascertained close to the ATD-LBD interface. Based on our functional assays, the splice may possibly affect the interaction between the receptor and auxiliary proteins. The modulatory effects of Neto1 and Neto2 on GluK1 splice variants might be mediated by multiple conserved positively charged patches (Li et al., 2019; Vinnakota et al., 2021). The complex between GluK2-Neto2 (He et al., 2021) provides a model that suggests K_183_ and K_187_ of GluK1 can potentially interact with a negative patch on Neto1 (D_140_-E_144_) and Neto2 (D_144_-E_148_). However, the splice insert appears to be positioned away from this interacting surface. Thus, it is possible that the splice residues may interact with Neto proteins when the receptor adopts a different conformation during the gating cycle. Additionally, splice residues could have an indirect or allosteric influence on the modulation of receptor functions and regulation by Neto proteins.

Our research, which encompasses structural, biochemical, biophysical, and functional investigations, highlights the significance of the unique N-terminal splice insert in the functions of GluK1 kainate receptors and opens avenues for further studies to understand its physiological effects. We also elucidated the important residues within the splice insert that could impact the modulatory behavior of auxiliary proteins. Our study emphasizes the need to investigate all possible combinations of KAR splice variants to better appreciate their contributions at different developmental stages. This comprehensive understanding of the distribution and functional diversity is essential for a rational therapeutic approach involving kainate receptors.

## Materials and methods

**Key resources table:** 

Reagent type (species) or resource	Designation	Source or reference	Identifiers	Additional information
Gene (*Rattus norvegicus*)	GRIK1-1a, GRIK1-2a, Neto1, Neto2	This paper		GRIK1 was used with mutations in the TM1 region to improve protein expression and stability
Strain, strain background (*Escherichia coli*)	BL21(DE3)	Sigma-Aldrich	CMC0016	competent cells
Cell line (*Homo sapiens*)	HEK293 GnTI- suspension-adapted cells	ATCC	CRL-3022	Used for expression of GluK1-1aEM for large-scale purification
Cell line (*Homo sapiens*)	HEK293 WT cells	ATCC	CRL-1573	Used for whole-cell patch-clamp electrophysiology
Cell line (*Homo sapiens*)	HEK293-T/17 cells	ATCC	CRL-11268	Used for outside-out patch-clamp electrophysiology
Antibody (Rabbit monoclonal)	Anti-His monoclonal antibody	Cell Signaling Technology	Cat. No. 12698	Used for co-immunoprecipitation/Western Blotting
Antibody (Rabbit polyclonal)	Anti-Neto1 polyclonal antibody	Sigma-Aldrich	SAB3500679	Used for co-immunoprecipitation/Western Blotting
Antibody (Mouse Monoclonal)	Anti-GFP	Sigma-Aldrich	G1546	Used for co-immunoprecipitation/Western Blotting
Antibody (Mouse Monoclonal)	Anti-Actin	Sigma-Aldrich	A3853	Used for co-immunoprecipitation/Western Blotting
Chemical compound	SYM2081 (2 S, 4R-4-methyl glutamate)	Tocris Bioscience	31137-74-3	Used to stabilize the receptor and electrophysiology experiments
Chemical compound	UBP301	Tocris Bioscience	569371-10-4	Used to stabilize the receptor and electrophysiology experiments
Chemical compound	Kainic acid	Tocris Bioscience	487-79-6	Used to stabilize the receptor and electrophysiology experiments
Chemical compound	L-Glutamic acid	Sigma-Aldrich	49449	Used to stabilize the receptor and electrophysiology experiments
Chemical compound	Sodium Butyrate	Sigma-Aldrich	8.17500	Added to boost protein production
Commercial assay kit	Bio-Beads SM-2	Bio-Rad	1523920	Used for detergent removal during nanodisc reconstitution
Recombinant DNA reagent	pEGBacMam vector	Eric Gouaux's lab (shared)		Used for protein expression
Recombinant DNA reagent	GluK1-1a and GluK1-2a in pRK7 vector	Mark Mayer’s lab (shared)		Used for electrophysiology experiments
Software algorithm	cryoSPARCv3	Nature Methods Punjani et al., 2017	DOI:10.1038/nmeth.4169	Used for single-particle data processing
Software algorithm	UCSF Motioncor2	Nature Methods Zheng et al., 2017	DOI 10.1038/nmeth.4193	Used for motion correction of cryo-EM data
Software, algorithm	UCSF ChimeraX	Goddard et al., 2018	RRID:SCR_015872	Molecular
Software, algorithm	UCSF Chimera	Pettersen et al., 2004	RRID:SCR_004097	Molecular
Software, algorithm	Coot	Emsley and Cowtan, 2004	RRID:SCR_014222	Protein Model
Software, algorithm	Phenix	Adams et al., 2010	RRID:SCR_014224	Protein Model
Software, algorithm	Clampfit	Molecular Devices	11.2	Electrophysiology data analysis
Software, algorithm	Fitmaster	HEKA Elektronik	v2x90.4	Electrophysiology data analysis
Software, algorithm	Dotmatics	GraphPad Prism	version 8.0.1	Used for statistical analysis and graphs/plots
Sequence-based reagent	Primers	This paper	PCR primers	Sequences given in Figure 4—source data 2
Other	Soybean polar lipids	Avanti Polar Lipids	541602 P	Used for the reconstitution of GluK1-1aEM in nanodiscs
Other	Dodecyl-β-maltoside (DDM)	Anatrace	D310LA	Used for solubilization of membrane fractions
Other	TALON Cobalt Resin	Clontech Takara	635653	Used for IMAC purification
Other	Protein A Agarose Beads	Thermo Scientific	20334	Used for pull-down assays
Other	cOmplete Protease Inhibitor Cocktail	Roche (Merck)	11697498001	Added to buffers used for protein purification

HEK293 WT, HEK293 GnTI^-^S, and HEK293-T/17 cells were procured from the American Type Culture Collection (ATCC, USA), accompanied by an authentication certificate and a mycoplasma-free certificate to ensure their quality and integrity.

### Whole-cell patch-clamp electrophysiology

A whole-cell patch-clamp analysis was performed to understand the functional differences between GluK1-1a and GluK1-2a, as well as their modulation by Neto proteins. HEK293 WT mammalian cells were seeded on siliconized glass coverslips in 35 mm dishes using Dulbecco’s Modification of Eagle’s Medium (DMEM) containing 10% fetal bovine serum (FBS), 2 mM glutamine, and 10 units/mL Penicillin-Streptomycin 24 hr before transection. Cells were transfected with rat (*Rattus norvegicus*) *GRIK1-1a* or *GRIK1-2a* cloned in pRK7 (in the presence or absence of r*Neto1*/r*Neto2* cloned in IE-pRK8 as indicated) with EGFP expressing plasmid using Xfect (Clontech) according to manufacturer’s instructions (receptor: Neto in 1: 3 and Receptor: EGFP in 1:1 DNA concentration). Similar protocols were followed to test the functionality of the *GRIK1-1a_EM_* and *GRIK1-1a* splice mutant constructs. The whole-cell patch-clamp recording was performed with an EPC 10 USB amplifier (HEKA) 24–48 hr post-transfection. Cells were lifted using 1.5 mm diameter thin wall glass capillary tubes (30–0066 Harvard Apparatus), pulled to a fine tip with a Sutter P-1000 micropipette puller (Sutter Instruments, Novato, CA) containing internal solution (10 mM HEPES pH 7.4, 100 mM CsF, 30 mM CsCl, 4 mM NaCl, 0.5 mM CaCl_2_, 5 mM EGTA and 300 mOsm). Cells were continuously perfused with external/bath solution (10 mM HEPES pH 7.4, 150 mM NaCl, 2.8 mM KCl, 1.8 mM CaCl_2_, 1 mM MgCl_2_, and 300 mOsm) through a peristaltic perfusion system (Multichannel systems). Current was measured by holding the membrane at –60 mV, using a 2 kHz low-pass filter. Ligands (10 mM glutamate, 1 mM kainate, 2 mM SYM2081, or 10 µM UBP301) were applied through a double-barrelled theta pipette connected with an ultra-fast piezo-based perfusion system (Multichannel systems) *via* v8 Perfusion Fast-Step System (VC-77SP). Recordings were controlled and measured using Patchmaster-v2x90.2 (Heka Elektronik). The raw files were analyzed using Clampfit 11.2 (Molecular Devices) and Fitmaster-v2x90.4 (HEKA Elektronik). The data was fitted to logistic curves in GraphPad Prism (GraphPad Software Inc). All traces were normalized before being used for calculations. The single exponential two-term fitting (Levenberg-Marquardt) was used to estimate the rate of desensitization (Ʈ_Des_) for 100ms glutamate application, measured as the decline of the current from 80% of its peak amplitude. Mean-weighted Tau (τ_Des_) values were determined using the formula [(τ1×amplitude1) + (τ2×amplitude2)]/[amplitude1 +amplitude2] where the tau values are the time constants from the exponential fit and the amplitudes are the assessed contributions of each component to the total peak current amplitude. To compute the recovery from desensitization, a series of paired-pulse experiments were performed with varying time pulses, where the amplitudes of the test pulse were normalized to that of the desensitizing pulse (calculated as relative amplitude) and plotted in comparison to the time (in seconds) between these two pulses. The recovery rate (Ʈ_Recovery_) was obtained by fitting the one‐phase association with an exponential function. Dose-response experiments were performed for GluK1-1a (co-expressed with or without Neto proteins) and GluK1-2a with different concentrations of glutamate or kainate in the range of 1 µM to 3 mM or 0.5 µM to 600 µM, respectively. Dose-response values were calculated as the percentage of maximum response against log[agonist] concentrations and fitted using variable slope (Hill’s equation) in GraphPad Prism 8.0.1. For calculating kainate efficacy (I_K_/I_G_), the ratio of peak amplitudes obtained from the same cell evoked first by glutamate followed by kainate were employed. A ratio of peak amplitudes obtained at +90/–90 mV was utilized to calculate the rectification index. We investigated the voltage-dependent endogenous polyamine block by measuring current-voltage relationships for the wild-type GluK1 receptors in the absence or presence of Neto proteins. Current-voltage (IV) plots were prepared using the voltage ramp from –90 to +90 mV with a 10 mV increment step only after complete recovery of the receptor, and the current amplitude was normalized to that obtained at –90 mV. For the mutant receptors, current values were obtained only for 3 voltage steps, –90, 0, and +90 mV. For kainate-evoked currents, the percentage desensitization was calculated. The steady-state current measured at the end of 1 s kainate application was divided by peak current. This ratio was then subtracted from 1 and multiplied by 100 to give percentage (%) desensitization (Fisher and Fisher, 2014).

### Outside-out patch electrophysiology

HEK 293 T/17 cells were used for outside-out patch experiments 48–96 hr post transfection. Cells were transfected with *GRIK1-1a* or *GRIK1-2a* cloned in pRK7 (in the presence or absence of rat (*Rattus norvegicus*) r*Neto1*/r*Neto2* cloned in IE-pRK8 as indicated) with EGFP expressing plasmid at DNA ratios of 3:0.5 for receptor alone and 2:8:0.5 with Neto proteins, respectively, using TransIT-LT1 Transfection Reagent according to manufacturer’s instructions.

The extracellular solution used for the experiment contained 10 mM HEPES pH 7.3, 150 mM NaCl, 2.8 mM KCl, 2 mM CaCl_2_, 1 mM MgCl_2_, and 10 mM glucose. The intracellular solution contained 10 mM HEPES pH 7.3, 110 mM CsF, 30 mM CsCl, 5 mM EGTA, 4 mM NaCl, and 0.5 mM CaCl_2_.

Patch micropipettes were pulled using a P-97 Sutter puller, and the resistance was maintained at 2.3–2.6 megaohms. After forming the whole-cell configuration, the patch was pulled away from the cell to facilitate an outside-out patch. The cells were voltage-clamped at –70 mV, and a saturated concentration of glutamate (10 mM) was rapidly applied using a three-barrelled theta glass attached to a Siskiyou MXPZT-300 solution switcher. Glutamate applications of 1ms were used to determine deactivation, while 100 or 1000 ms applications were used to assess desensitization.

### Statistical analysis

Comparisons between wild-type receptors, EM, or mutant constructs were obtained using an unpaired *t-*test (two-tailed, with or without the Welch test) or Brown-Forsythe and Welch ANOVA, followed by Dunnett’s multiple comparisons. Statistical analysis was carried out in GraphPad Prism, version 8.0.1. p-values <0.05 were considered statistically significant and are reported (*p<0.05, **p<0.01, ***p<0.001, ****p<0.0001).

### Site-directed mutagenesis (SDM)

Based on our electrophysiology analysis of the wild-type GluK1-1a/GluK1-2a receptors, structural analysis of GluK1-1a_EM_, and recent reports that suggest that the presence of the positive patches in GluK2 ATD affects the interaction with Neto proteins (Li et al., 2019; He et al., 2021; Vinnakota et al., 2021), we performed SDM to understand the role of splice residues in the receptor kinetics. All splice mutations were introduced in the wild-type (species)*GRIK1-1a* pRK7 construct for electrophysiology, as well as the *GRIK1-1a_EM_*-EGFP-His_8_-pEGBacMam construct for surface expression and pull-downs using the (overlap PCR) ligation-free cloning approach (Zhang et al., 2017). In brief, we performed the two sets of PCR using standard cloning primers (~300 bp upstream and downstream of the mutation) and the mutant primers as listed in Figure 4—source data 2 to obtain the fragment containing our mutation of interest and flanking regions corresponding to the wild-type GluK1-1a. Next, the obtained fragment was used as a megaprimer to amplify the complete GluK1-1a vector backbone that would now contain the mutation. This PCR product was DpnI digested for 1 hr at 37 °C to remove parental DNA and was transformed into DH5α strain of *E. coli*. Clones were confirmed by sequencing. Initially, the positively charged and other residues of the splice were substituted with alanine. Later, charge reversal mutants (K and/or H to E) were also prepared to understand their role in the receptor kinetics as well as interaction with Neto proteins. The mutants prepared are summarized in Figure 4—source data 2.

### Co-immunoprecipitation (in vitro)

To understand the effect of mutations on GluK1-1a and Neto1/2 interaction, co-immunoprecipitation was carried out using an anti-His monoclonal antibody against the receptor. Similar constructs and transfection protocols were followed for surface expression analysis. Cells were pelleted 65–70 h post-transfection and washed with TBS (20 mM Tris pH 8, 150 mM NaCl). These cells were sonicated and solubilized in 500 uL lysis buffer (20 mM Tris pH 8, 150 mM NaCl, 1% glycerol, protease inhibitor cocktail, 30 mM DDM). Post solubilization, debris was removed by centrifuging at 17,000 × g for 45 min at 4 °C. The supernatant was incubated for pre-clearing with 20 µL of pre-equilibrated Protein A agarose beads (Thermo Scientific) for 1 hr on a rotator at 4 °C. Post-pre-clearing, ~10% sample was saved as input, and the rest was used for pull-downs. Simultaneously, 2 ug of anti-His antibody (host: rabbit, Cat. No.-12698, Cell Signaling Technology) was added to 40 µL pre-equilibrated Protein A agarose (Thermo Scientific) and incubated for 1 hr at 4 °C, followed by the addition of pre-cleared lysate. It was further incubated at 4 °C overnight (14–16 hr). The unbound fraction was removed and washed four times with 500 µL wash buffer (20 mM Tris pH 8, 150 mM NaCl, 1% glycerol, 0.75 mM DDM) to remove non-specific interactions. Protein was eluted in 30 µL elution buffer (100 mM Tris pH 6.8, 12% glycerol, 4% SDS, 10 mM DTT, 2% β- mercaptoethanol) by heating at 95 °C for 10 min. Rabbit IgG controls were set up to confirm the validity of the experiment. To analyze the pull-down, 8% SDS-PAGE was used, followed by a western transfer. To detect the internal control (actin), the receptor, and the co-immunoprecipitated Neto proteins, the immunoblots were probed using anti-actin (mouse, A3853, Sigma), anti-His (rabbit, 12698, Cell Signaling Technology), and anti-Neto1 (rabbit, SAB3500679, Sigma) or anti-GFP (mouse, G1546, Sigma) antibodies.

### Construct design for expression and purification of rat GluK1-1a_EM_

To obtain functional GluK1 receptors, rat (*Rattus norvegicus*) *GRIK1* with ATD splice insert and the shortest C-terminal domain, *GRIK1-1a* (1–871 amino acid residues), was cloned in the pEGBacMam vector. The receptor was cloned in-frame with a thrombin recognition site (LVPRGSAAAA), EGFP (A_207_K; non-dimerizing mutant), and His_8_ at the C-terminus. The wild-type protein generated very low amounts of the tetramer, as observed in fluorescence-assisted size-exclusion chromatography (FSEC). Therefore, based on the alignments of the sequence with GluK2_EM_, GluK3_EM_ and GluA2, we mutated free cysteines in the TM1 region to residues corresponding to those of GluK2_EM_ or GluA2 [1 x Cys (C_576_S), 2 x Cys (C_552_Y, C_557_V) and 3 x Cys (C_552_Y, C_557_V, C_576_S)] to obtain good yields of the tetrameric receptor. All clones were confirmed by restriction digestion and sequencing. The expression of all the mutants was confirmed by immunoblotting against His-tag and FSEC. GluK1-1a with 2 x Cys mutations (C_552_Y, C_557_V) gave us the best receptor quality as observed in FSEC and, therefore, was used as GluK1-1a_EM_ for large-scale purification for structural studies.

### GluK1-1a_EM_ expression and purification

Three liters of Human embryonic kidney (HEK) 293 GnTI^-^ suspension-adapted cultures (~1.5–2.0 × 10^6^ cells/mL) were transfected (or infected with virus) with rat *GRIK1-1a_EM_* plasmid at 0.5 µg/mL or baculovirus, prepared in DH10Bac as per established protocol (Goehring et al., 2014) using polyethyleneimine (PEI-MAX, Polysciences; 1 DNA: 3 PEI w/w) as the transfection agent (or, infected at a multiplicity of infection of ~2). To boost the protein production, 10 mM sodium butyrate (Sigma) was added 16 hr post-transfection/infection, and cultures were incubated at 30 °C for protein expression. Cells were harvested 65–70 h after transfection/infection, washed with buffer containing 20 mM Tris pH 8 and 150 mM NaCl, and stored at –80 °C until further processing. The cell pellet was resuspended (20 mL/L culture volume) in lysis buffer (20 mM Tris pH 8, 150 mM NaCl, and protease inhibitor cocktail, Roche). These resuspended cells were disrupted using sonication (QSonica sonicator, three cycles of 90 s; 10 s ON/20 s OFF; temperature cut-off: 15 °C). The lysate was clarified using low-speed spin (4307 × g for 20 min at 4 °C). The membranes were pelleted using ultracentrifugation (118,991 × g for 1 hr at 4 °C). Solubilization of membrane fraction was done in buffer containing 20 mM Tris pH 8, 150 mM NaCl, 30 mM DDM (Anatrace, D310LA), 5% glycerol, 10 mM imidazole, and protease inhibitor cocktail for 1 hr on rotator at 4 °C. The non-solubilized fraction was separated by centrifugation at 47,850 × g for 1 hr at 4 °C and 4 mL of cobalt-charged TALON resin (Clontech, Takara) was added to the solubilized fraction for batch binding (3 hr at 4 °C). Beads were harvested. The column was packed and washed with wash buffer (20 mM Tris pH 8, 150 mM NaCl, 1 mM DDM, 1% glycerol, 40 mM imidazole) until OD_280_ reached zero. The bound receptor was eluted with elution buffer (20 mM Tris pH 8, 150 mM NaCl, 1 mM DDM, 1% glycerol, 250 mM imidazole). The peak fractions were pooled, concentrated at 1.3 mg/mL, and digested overnight at 4 °C with thrombin (1:100 w/w). Simultaneously, purified GluK1-1a_EM_ was also reconstituted in MSP1E3D1 nanodiscs with soybean polar lipids (Avanti Polar Lipids, 541602 P) in a 1:2:140 ratio (GluK1-1a:MSP:lipids) following already established protocols (Chen et al., 2017; Gao et al., 2016; Ritchie et al., 2009). In brief, the purified GluK1-1a_EM_ in DDM was incubated with lipids, MSP1E3D1, 14 mM sodium cholate, and 1 mM PMSF for 30 min at 4 °C. The reconstitution of protein in nanodisc was initiated by removing detergent using equilibrated Bio-Beads SM-2 (biorad, 150 mg/mL) for 4–6 hr at 4 °C on an end-to-end rotator. The thrombin-digested GluK1-1a_EM_ receptors in DDM, or nanodisc-reconstituted receptors, were further purified via gel filtration (Superose 6 10/300, GE) in buffer containing 20 mM Tris pH 8, 150 mM NaCl, 0.5% glycerol, and 0.75 mM DDM (no detergent for nanodisc protein). The peak fractions were pooled and concentrated to ~0.6 mg/mL (final DDM concentration,~7.5 mM) or ~0.9 mg/mL GluK1-1a_EM_ in nanodisc. All the affinity elution fractions and final purified protein were confirmed for purity and homogeneity using SDS-PAGE and FSEC, respectively. In the subsequent sections, purified GluK1-1a_EM_ in detergent and nanodisc will be called GluK1-1a_EM_ DDM and GluK1-1a_EM_ ND, respectively.

### Cryo-EM sample preparation and data collection

Before grid preparation, the purified protein was incubated with 2 mM 2 S, 4R-4-methyl glutamate (SYM2081) to capture the receptor in the desensitized state. The concentration of SYM2081 was confirmed by electrophysiology, and the stability of the receptor-SYM2081 complex was tested using FSEC.

GluK1-1a_EM_ DDM: Double application of 3 μL protein (~0.6 mg/mL) SYM2081 complex was carried out on glow-discharged gold grids (R 0.6/1, 300 mesh, Quantifoil). The grids were blotted for 4.5 s and 3.5 s, respectively (0 blot force). Vitrobot temperature was maintained at 12 °C with 100% humidity, and the sample was vitrified in liquid ethane. The clipped grids were loaded into a 300 keV Titan Krios microscope equipped with a Falcon III direct detector camera (4k × 4k). Movies were recorded in counting mode with a nominal magnification of 59,000 X (pixel size: 1.38 Å), and the defocus range of −2.0 to −3.2 μm increased in steps of 0.3. Each movie comprises 25 frames with a total exposure of 60 s. A dose rate of 0.78 e^-^/frame was applied, with the total dose being 19.5 e^-^/Å^2^.

GluK1-1a_EM_ ND: Double application of 2 μL protein (~0.9 mg/mL) SYM2081 complex was carried out on glow-discharged gold grids (1.2/1.3, 200 mesh, Quantifoil). Grids were blotted for 3 s and 10 s, respectively, followed by vitrification. The clipped grids were loaded into a 300 keV Titan Krios microscope equipped with a K2 direct-detector camera (Gatan). The movies were recorded in super-resolution mode with an energy filter (20 eV slit) and a pixel size of 1.41 Å. Each movie was composed of 30 frames with a total exposure of 12 s. A dose rate of 1.36 e^-^/frame was applied, with a total dose of 40.8 e^-^/Å^2^.

### Single-particle analysis

All movies (GluK1-1a_EM_ DDM-SYM: 1100, GluK1-1a_EM_ ND-SYM: 1535) were motion-corrected using UCSF Motioncor2 (Zheng et al., 2017). Bad micrographs were removed manually post-contrast transfer function (CTF) estimation using CTFFIND4 (Rohou and Grigorieff, 2015). Manually picked particles (~1000) were 2D classified and used as templates for auto picking in RELION or cryoSPARCv 3. For GluK1-1a_EM_ DDM-SYM and GluK1-1a_EM_ ND-SYM, the auto picked particles in cryoSPARCv3 were cleaned up with multiple rounds of 2D classification. Particles in the best classes were used for training TOPAZ for automated particle picking. For GluK1-1a_EM_ DDM-SYM, initially, 13,750 particles were picked; post iterative rounds of 2D and 3D classification, 5372 particles were used for final 3D reconstruction. In the case of GluK1-1a_EM_ ND-SYM, initially, 1,97,908 particles were picked and subjected to multiple rounds of clean-up using 2D and 3D classification. Finally, 24,531 particles were used for the final 3D reconstruction.

The final particles were corrected for local motion in cryoSPARCv3, followed by refinement using C1 symmetry for both GluK1-1a_EM_ DDM-SYM and GluK1-1a_EM_ ND-SYM. Since we had less information on TMD in both the detergent and nanodisc forms, as observed in 2D classes, we performed local refinement for ATD and LBD using an ECD mask for GluK1-1a_EM_ DDM-SYM and GluK1-1a_EM_ ND-SYM, which improved map resolution to 8.01 Å and 5.2 Å (0.143 FSC), respectively. For GluK1-1a_EM_ DDM-SYM, local refinement using a mask for full-length structure yielded a resolution of 8.2 Å. The 3D maps were sharpened via cryoSPARCv3 (Punjani et al., 2017) or DeepEMhancer (Sanchez-Garcia et al., 2021) using a mask from the cryoSPARCv3 refinement output. Local Resolution was estimated using BlocRes (Cardone et al., 2013) in cryoSPARCv3 or Phenix1.19.2 (Afonine et al., 2018a).

### Model building

Tetrameric assembly for the GluK1-1a_EM_ ND-SYM model was built using crystal structures of individual domains (ATD, not yet published, and LBD, PDB: 3C32, GluK1 LBD crystal structure with kainate) fitted into the EM map (5.2 Å) in UCSF Chimera (Pettersen et al., 2004). Furthermore, for building GluK1-1a_EM_ ND transmembrane domain 3 and GluK1-1a_EM_ DDM trans-membrane domain, GluK2 TMD (PDB:5KUF) was used due to high identity (~95%) for fitting into EM density. Phenix1.19.2 (Afonine et al., 2018b) and Namdinator (Kidmose et al., 2019) were used to improve the model via the rigid body and Molecular Dynamics based flexible fitting, respectively. The final models fit well into the EM map of GluK1-1a_EM_ ND-SYM, GluK1-1a_EM_ DDM-SYM ECD, and FL, respectively, with a definite density for ATD, LBD, and TMD (pre-M1, M1, M3, and M4). Coot 0.9.4 (Emsley and Cowtan, 2004) was used to analyze the final models, and chimera/chimeraX (Pettersen et al., 2021; Pettersen et al., 2004) was used for figure preparation.

### Supplementary methods

#### Spatiotemporal distribution of exon 9 of GluK1 using transcriptomics data analysis

To understand the abundance of *GRIK1-1* splice in the human brain, we resorted to RNA-seq data from the BrainSpan atlas that constitutes various databases to study transcriptional mechanisms in human brain development. The RNA-seq data were downloaded for *GRIK1* (https://www.brainspan.org/rnaseq/gene/1099967), plotted in Excel (x-axis: regions of the brain; y-axes: log_2_ transformed normalized expression intensity values in Reads Per Kilobase of exon model per Million mapped reads, RPKM and age of donor). The heat maps were generated using the Excel file (CSV format) and RStudio (https://www.rstudio.com/) to determine the presence of GluK1 in various regions of the brain at different developmental stages, as shown in Figure 1. Furthermore, we narrowed it down to the *GRIK1-1* splice (exon 9; start position 30968845, 45 nucleotides in length) and tried to understand how it overlaps with the entire *GRIK1* gene expression. These data explored the presence of the ATD splice irrespective of which C-terminal splice variant is present.

## Data Availability

The cryo-EM density reconstructions and final models were deposited in the Electron Microscopy Data Base (accession codes EMD-34076, EMD-34083, and EMD-34197) and the Protein Data Bank (accession codes 7YSJ, 7YSV, and 8GPR).All data generated or analysed during this study are included in the manuscript and supporting files; source data files have been provided. The following datasets were generated: DhingraS KumarJ 2023GluK1-1a in nanodisc captured in SYM2081 bound desensitized stateEMDataBankEMD-34076 DhingraS KumarJ 2023GluK1-1a extracellular domain captured in SYM2081 bound desensitized stateEMDataBankEMD-34083 DhingraS KumarJ 2023GluK1-1a receptor captured in the desensitized stateEMDataBankEMD-34197 DhingraS KumarJ 2023GluK1-1a in nanodisc captured in SYM2081 bound desensitized stateRCSB Protein Data Bank7YSJ DhingraS KumarJ 2023GluK1-1a extracellular domain captured in SYM2081 bound desensitized stateRCSB Protein Data Bank7YSV DhingraS KumarJ 2023GluK1-1a receptor captured in the desensitized stateRCSB Protein Data Bank8GPR The following previously published datasets were used: LeinES 2007GRIK1Brainspan1099967 MayerML 2008Crystal structure of GluR5 ligand-binding core in complex with sodium at 1.72 Angstrom resolutionRCSB Protein Data Bank3C32 MeyersonJR ChittoriS MerkA RaoP HanTH SerpeM MayerML SubramaniamS 2016GluK2EM with 2S,4R-4-methylglutamateRCSB Protein Data Bank5KUF MeyersonJR SelvakumarP 2021Structure of full-length GluK1 with L-GluRCSB Protein Data Bank7LVT

## Additional files

MDAR checklist
