# Supplementary material for: Functional implications of the exon 9 splice insert in GluK1 kainate receptors
Source: eLife. 2024 Nov 6;12:RP89755. doi: 10.7554/eLife.89755 (PMC11540303; doi:10.7554/eLife.89755)
Supplement: Figure 4—source data 2. — The various mutants used in the study and the primer sequences to make charge-neutral and charge-reversal mutants in GluK1-1a are tabulated. [file elife-89755-fig4-data2.docx]

| **K_368_ASGEVSKHLYKVWK_382_** | | **Forward primer**  **(5’-3’)** | | **Reverse primer**  **(5’-3’)** | |
| --- | --- | --- | --- | --- | --- |
| **Common cloning primers (AfeI/BsiWI: RE sites)** | | CTGAAGCAGCGCTGATGTACG | | CTTTCTCCCGTACGTATGTGATGG | |
| **Ala mutations (Charge neutral mutations)** | | | | | |
| K_375_H_376_-A | KASGEVSAALYKVWK | | GCCTCTGGTGAAGTGTCTGCCGCTTTGTATAAAGTGTGGAAG | | CTTCCACACTTTATACAAAGCGGCAGACACTTCACCAGAGGC |
| K_375/379/382_-A | KASGEVSAHLYAVWA | | GCCTCTGGTGAAGTGTCTGCCCACTTGTATGCCGTGTGGGCCAAGATTGGGATTTGGAAC | | GTTCCAAATCCCAATCTTGGCCCACACGGCATACAAGTGGGCAGACACTTCACCAGAGGC |
| Y_378_V_380_W_381_-A | KASGEVSKHLAKAAK | | GAAGTGTCTAAACACTTGGCTAAAGCGGCCAAGAAGATTGGGATTTGG | | CCAAATCCCAATCTTCTTGGCCGCTTTAGCCAAGTGTTTAGACACTTC |
| K_375_HLYKVWK_382_-8A | KASGEVSAAAAAAAA | | GCCTCTGGTGAAGTGTCTGCCGCTGCAGCTGCAGCCGCAGCTAAGATTGGGATTTGGAAC | | GTTCCAAATCCCAATCTTAGCTGCGGCTGCAGCTGCAGCGGCAGACACTTCACCAGAGGC |
| **Glu mutations (Charge reversal mutations)** | | | | | |
| K_375_H_376_-E | KASGEVSEELYKVWK | | GCCTCTGGTGAAGTGTCTGAGGAATTGTATAAAGTGTGGAAG | | CTTCCACACTTTATACAATTCCTCAGACACTTCACCAGAGGC |
| H_376_-E | KASGEVSKELYKVWK | | GCCTCTGGTGAAGTGTCTAAAGAATTGTATAAAGTGTGGAAG | | CTTCCACACTTTATACAATTCTTTAGACACTTCACCAGAGGC |
| K_375/379/382_H_376_-E | KASGEVSEELYEVWE | | GCCTCTGGTGAAGTGTCTGAGGAATTGTATGAGGTGTGGGAGAAGATTGGGATTTGGAAC | | GTTCCAAATCCCAATCTTCTCCCACACCTCATACAATTCCTCAGACACTTCACCAGAGGC |
| K_368/375/379/382_H_376_-E | EASGEVSEELYEVWE | | AAAGAGGAAGGAACTGAAGAGGCCTCTGGTGAAGTGTCT | | AGACACTTCACCAGAGGCCTCTTCAGTTCCTTCCTCTTT |
| K_368_-E | EASGEVSKKLYKVWK | | AAAGAGGAAGGAACTGAAGAGGCCTCTGGTGAAGTGTCT | | AGACACTTCACCAGAGGCCTCTTCAGTTCCTTCCTCTTT |

**Figure 4-table supplement 1.** GluK1-1a ATD splice mutants. The various mutants used in the study and the primer sequences to make charge-neutral and charge-reversal mutants in GluK1-1a are tabulated.
