# Supplementary figures and images for: Functional implications of the exon 9 splice insert in GluK1 kainate receptors
Source: eLife. 2024 Nov 6;12:RP89755. doi: 10.7554/eLife.89755 (PMC11540303; doi:10.7554/eLife.89755)

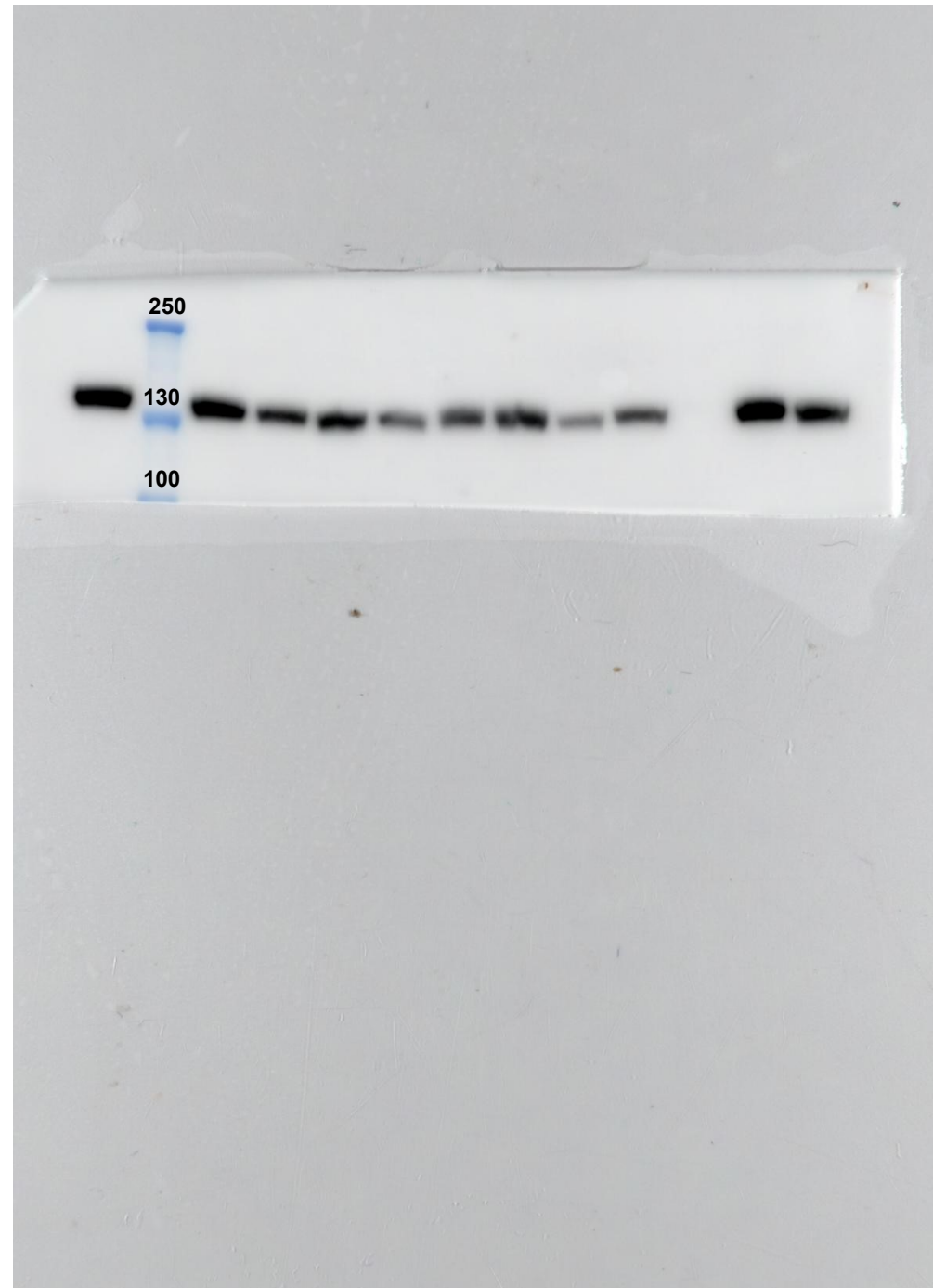

**His**  
**(GluK1 ~135 kDa)**

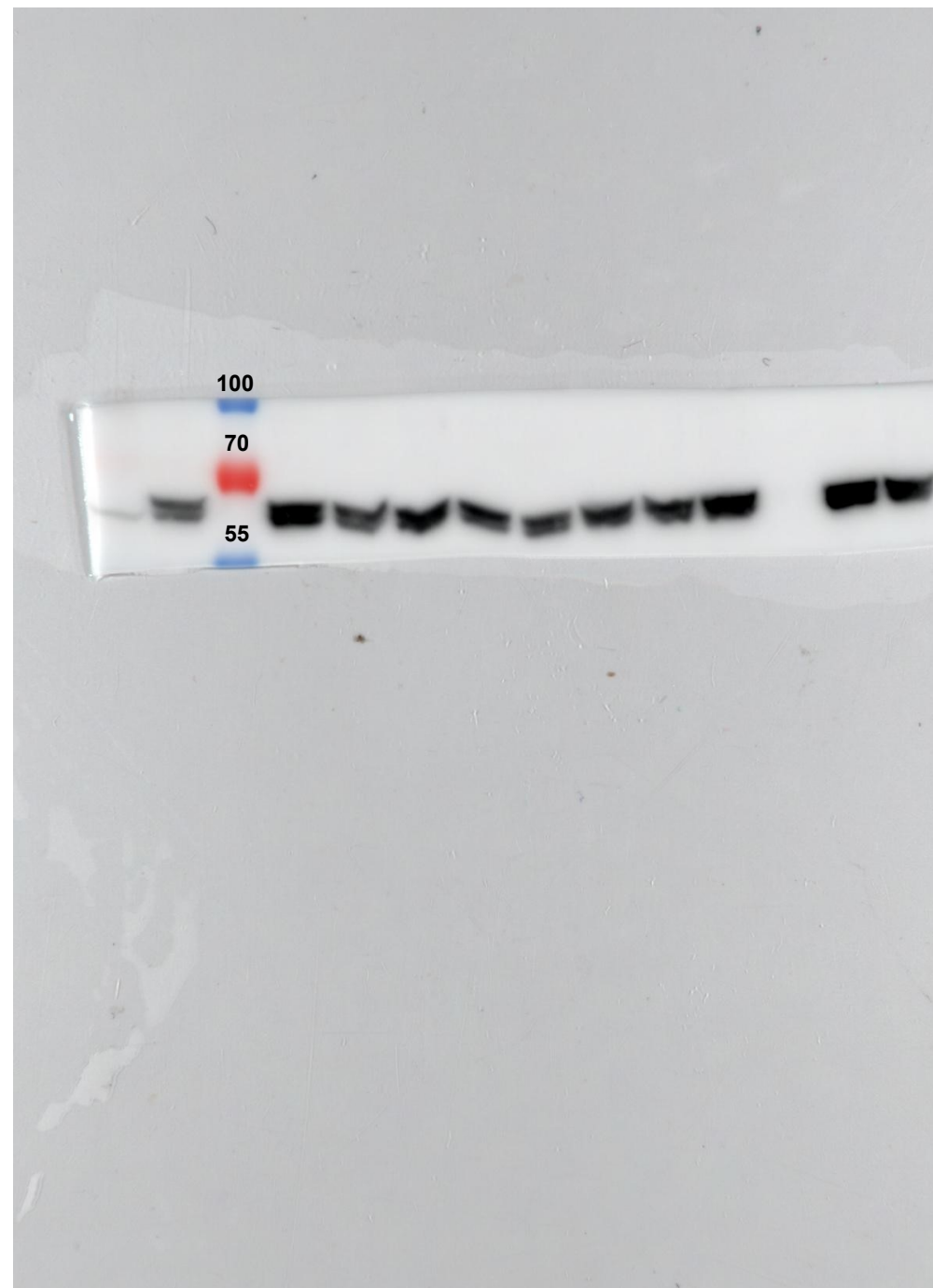

**Neto1**  
**(~58 kDa)**

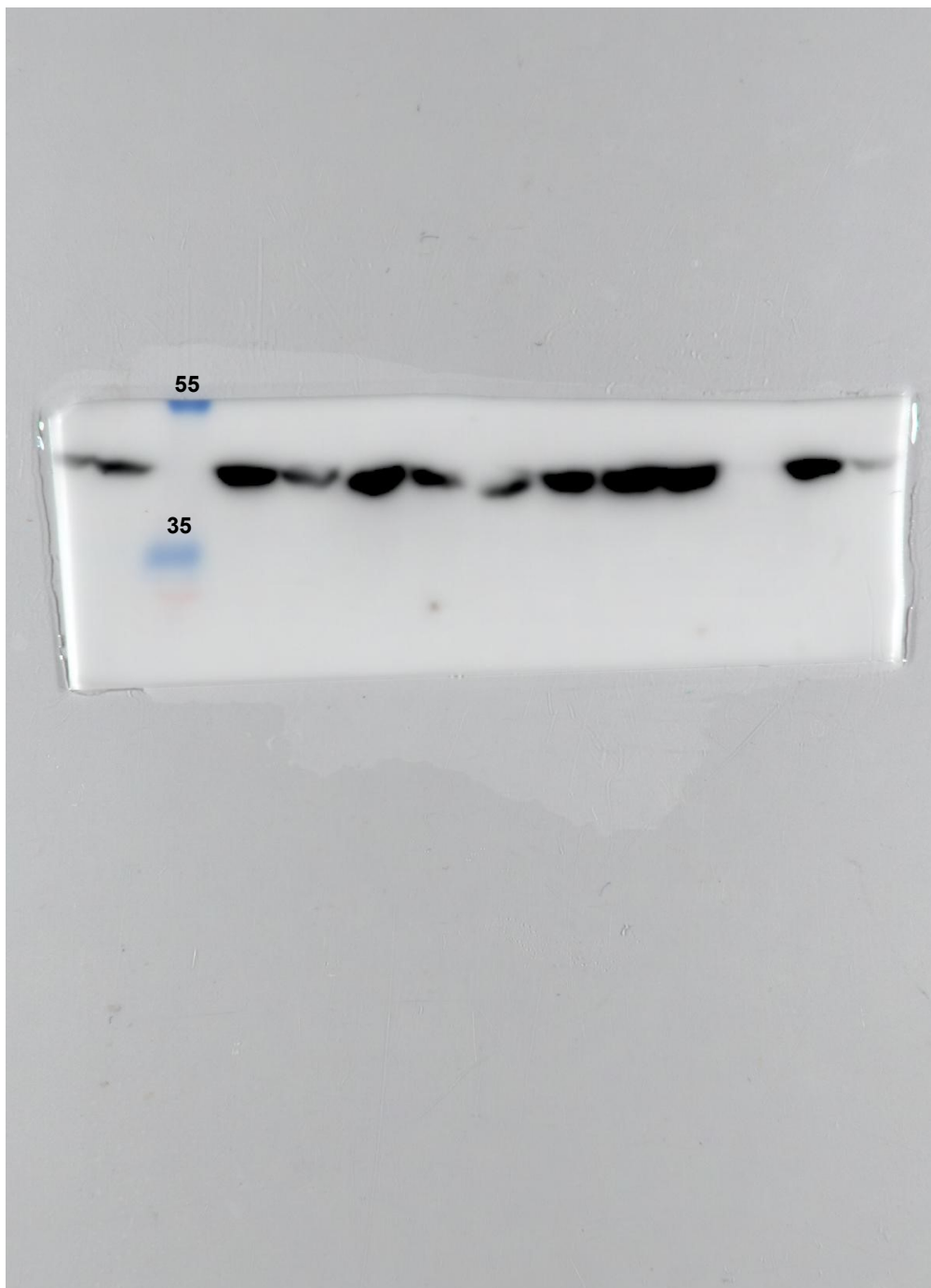

**Actin**  
**(~42 kDa)**

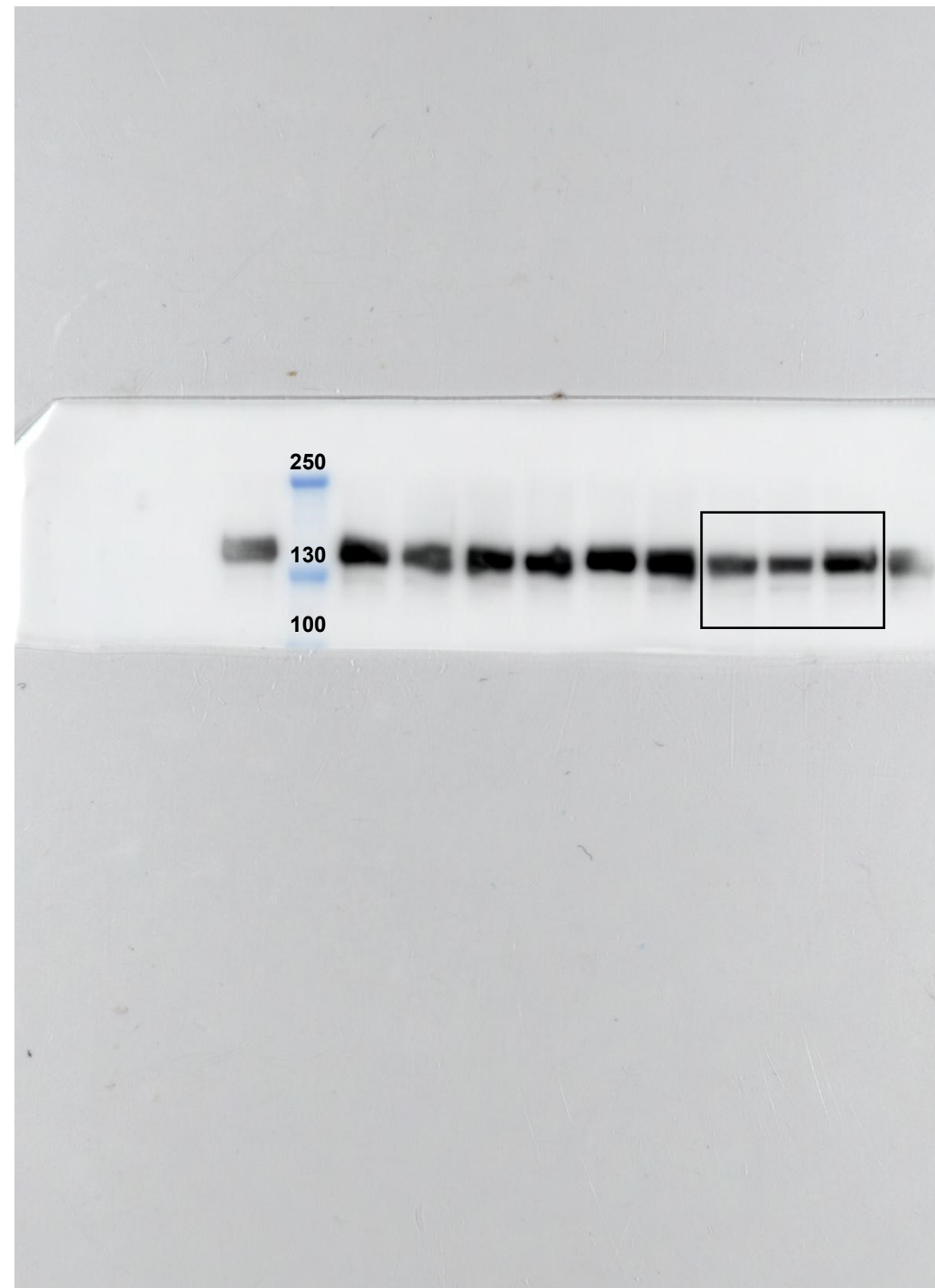

**His**  
**(GluK1 ~135 kDa)**

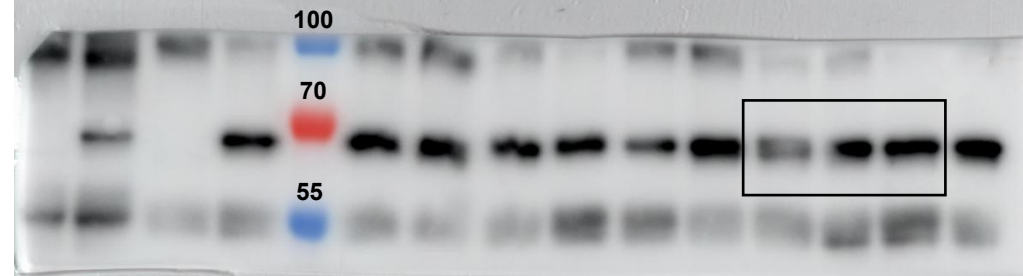

**Neto1**  
**(~58 kDa)**

Supplement: Figure 5—figure supplement 1—source data 1. [file elife-89755-fig5-figsupp1-data1.pdf]

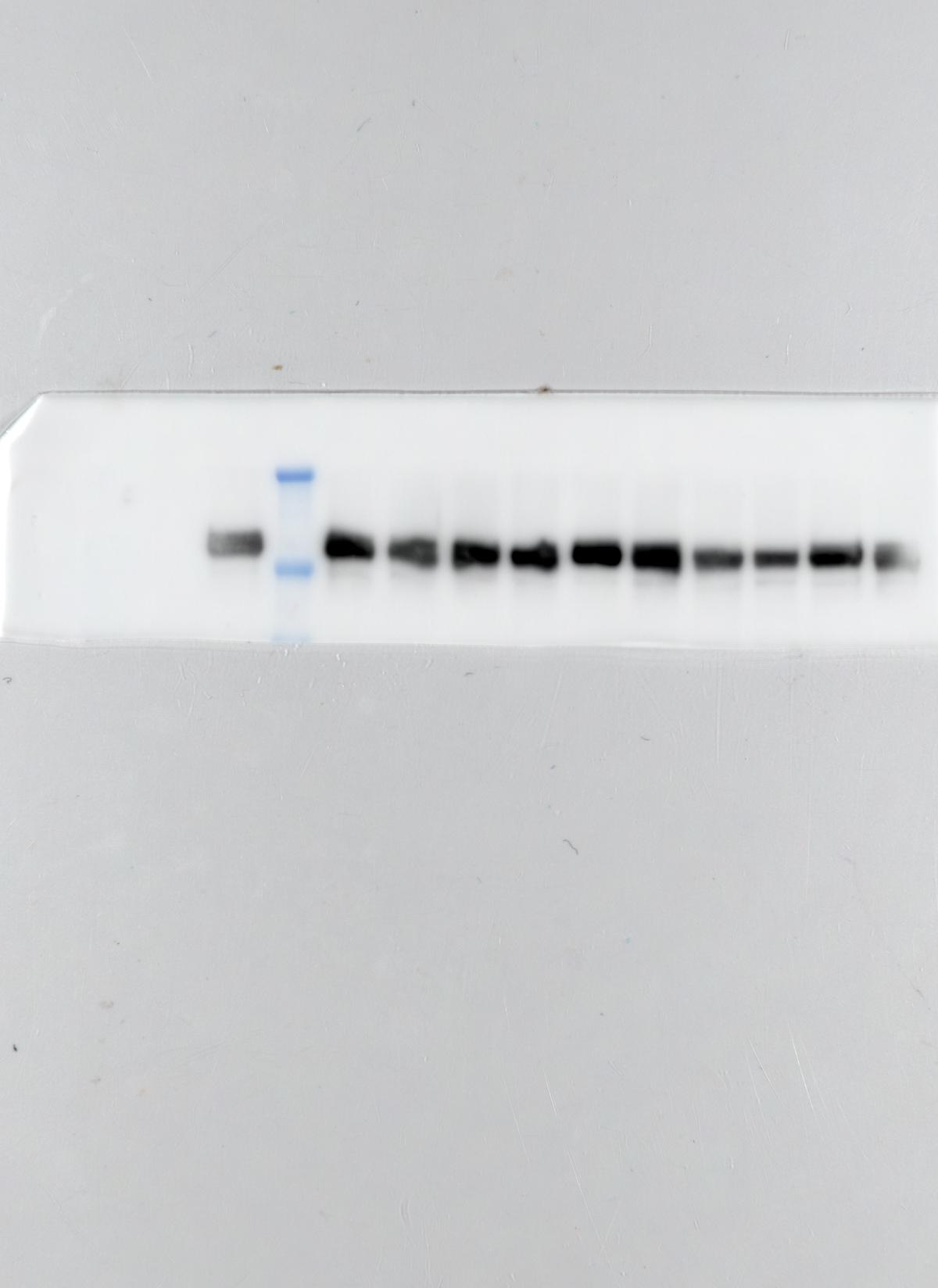

Supplement: Figure 5—figure supplement 1—source data 2. [file elife-89755-fig5-figsupp1-data2.zip › Figure 5-figure supplement 1A_jpeg/Fig5_FigSup1A_Eluate_1.jpg]

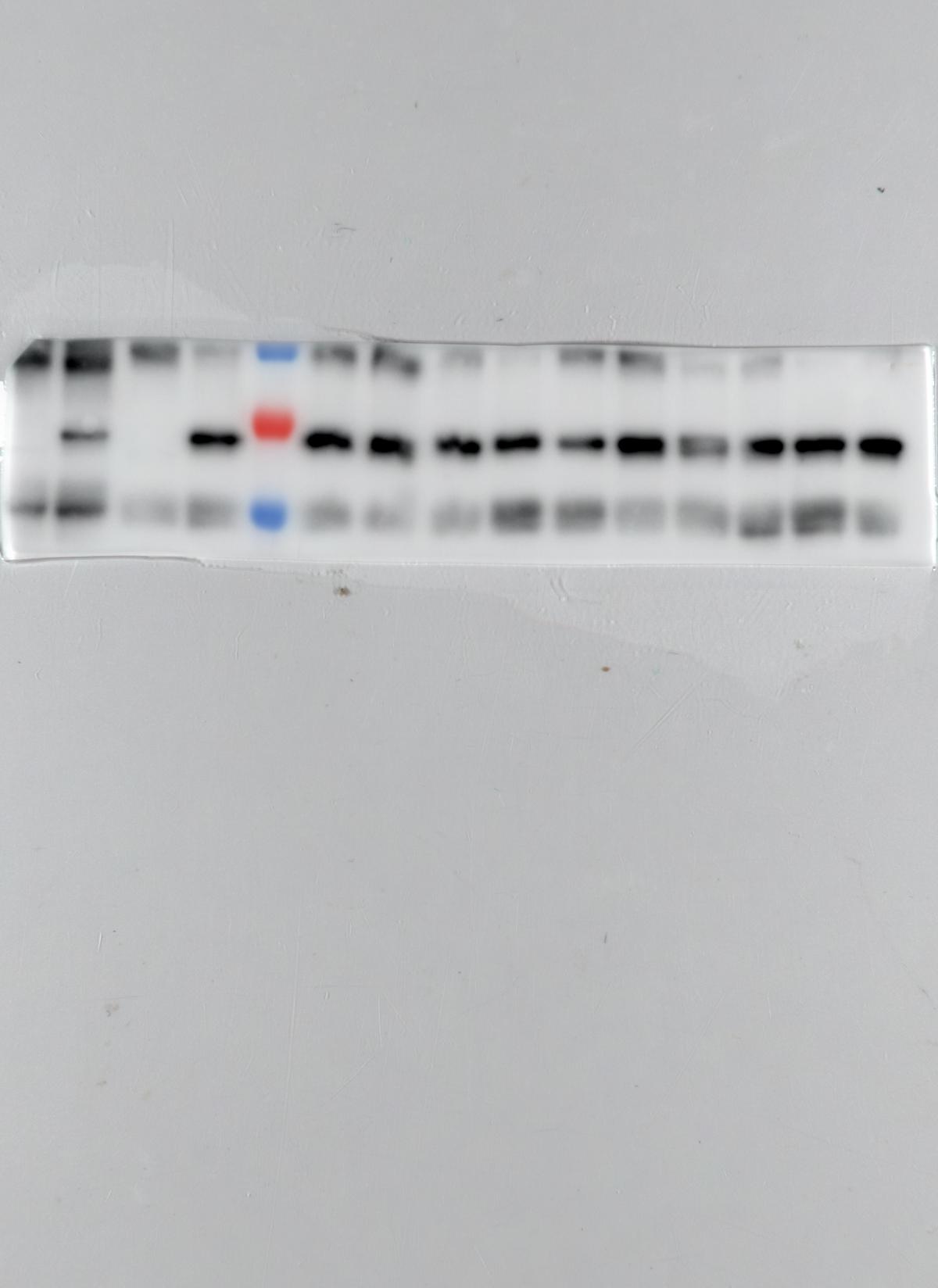

Supplement: Figure 5—figure supplement 1—source data 2. [file elife-89755-fig5-figsupp1-data2.zip › Figure 5-figure supplement 1A_jpeg/Fig5_FigSup1A_Eluate_2.jpg]

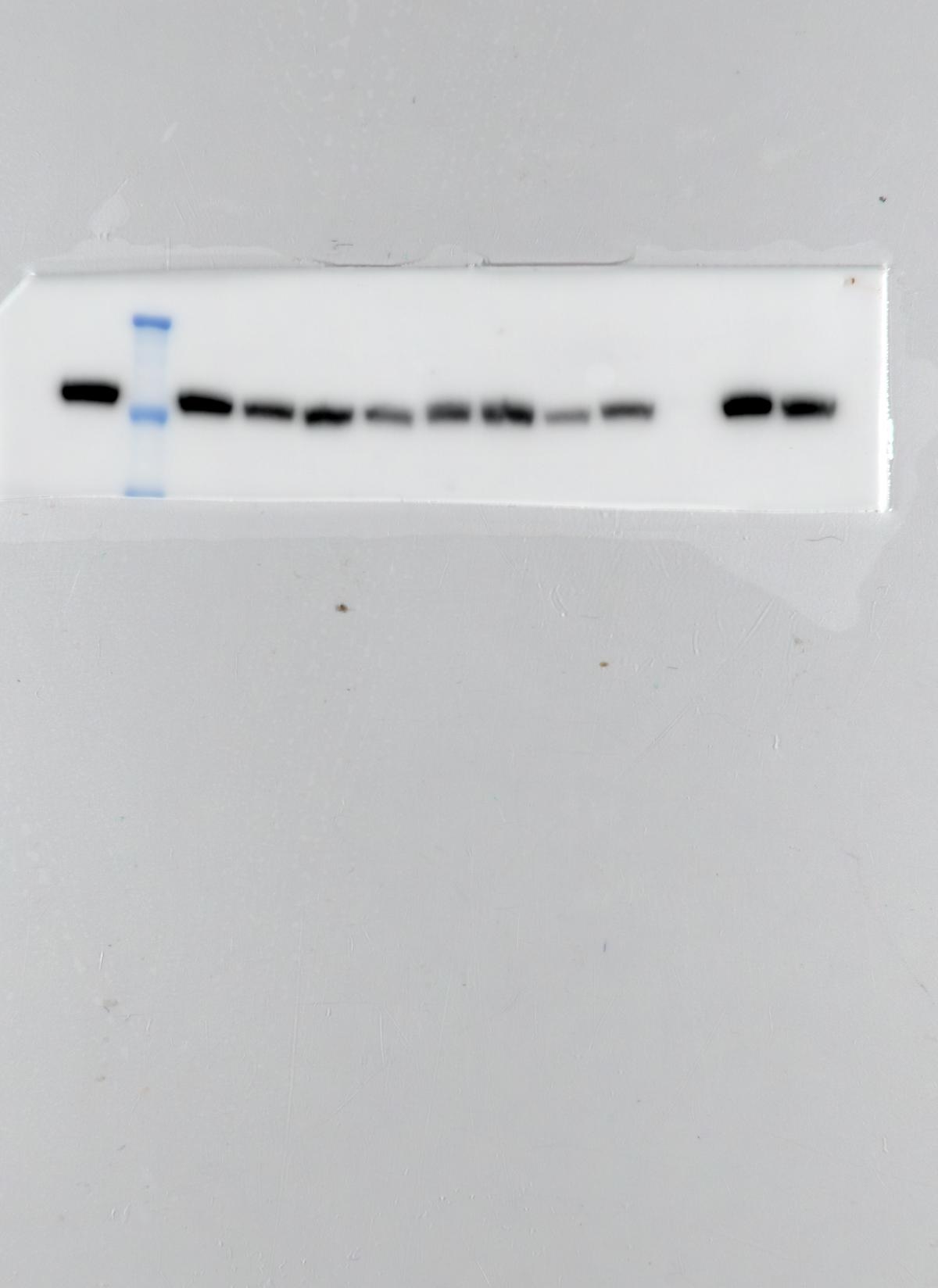

Supplement: Figure 5—figure supplement 1—source data 2. [file elife-89755-fig5-figsupp1-data2.zip › Figure 5-figure supplement 1A_jpeg/Fig5_FigSup1A_Input_1.jpg]

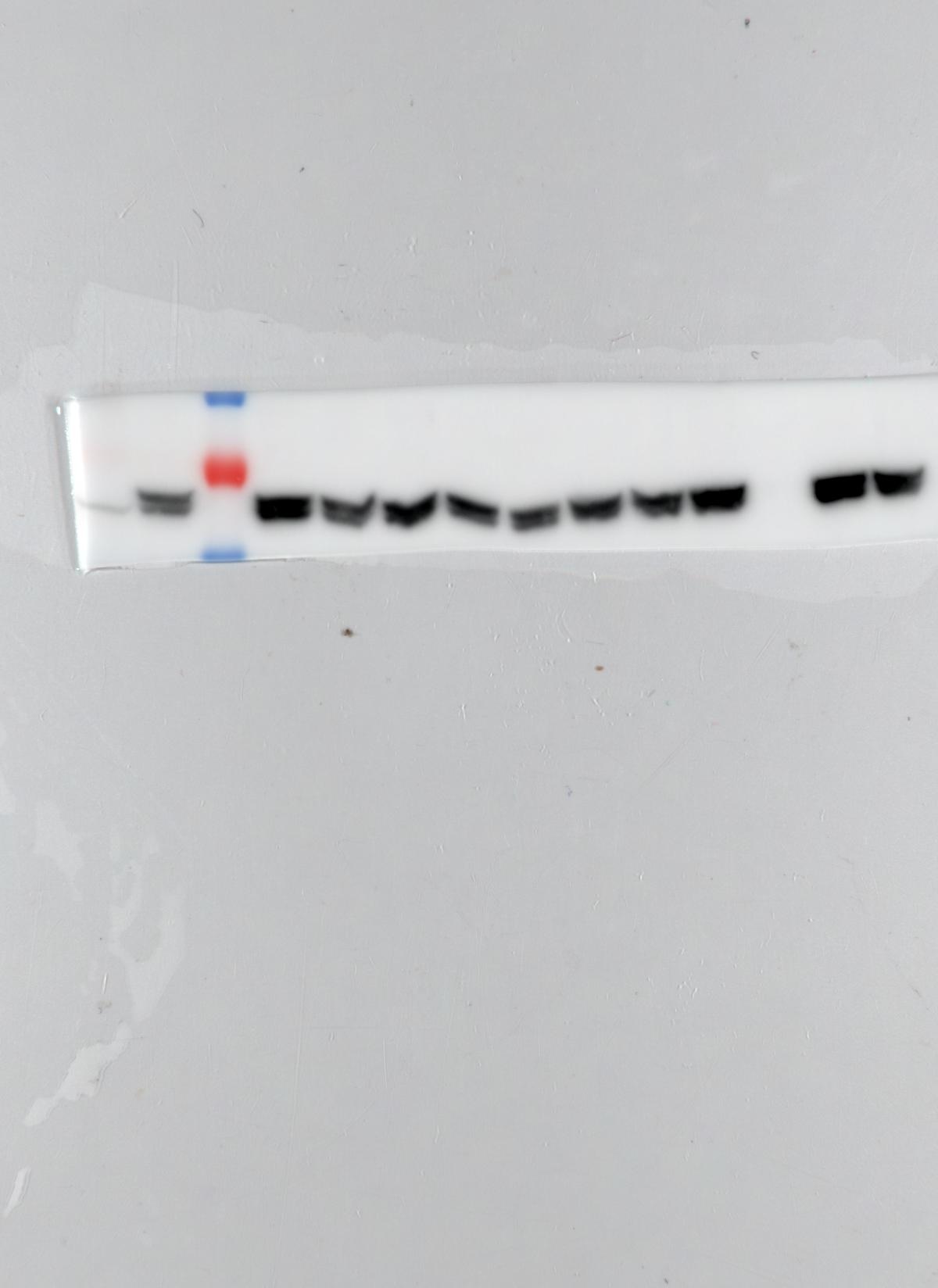

Supplement: Figure 5—figure supplement 1—source data 2. [file elife-89755-fig5-figsupp1-data2.zip › Figure 5-figure supplement 1A_jpeg/Fig5_FigSup1A_Input_2.jpg]

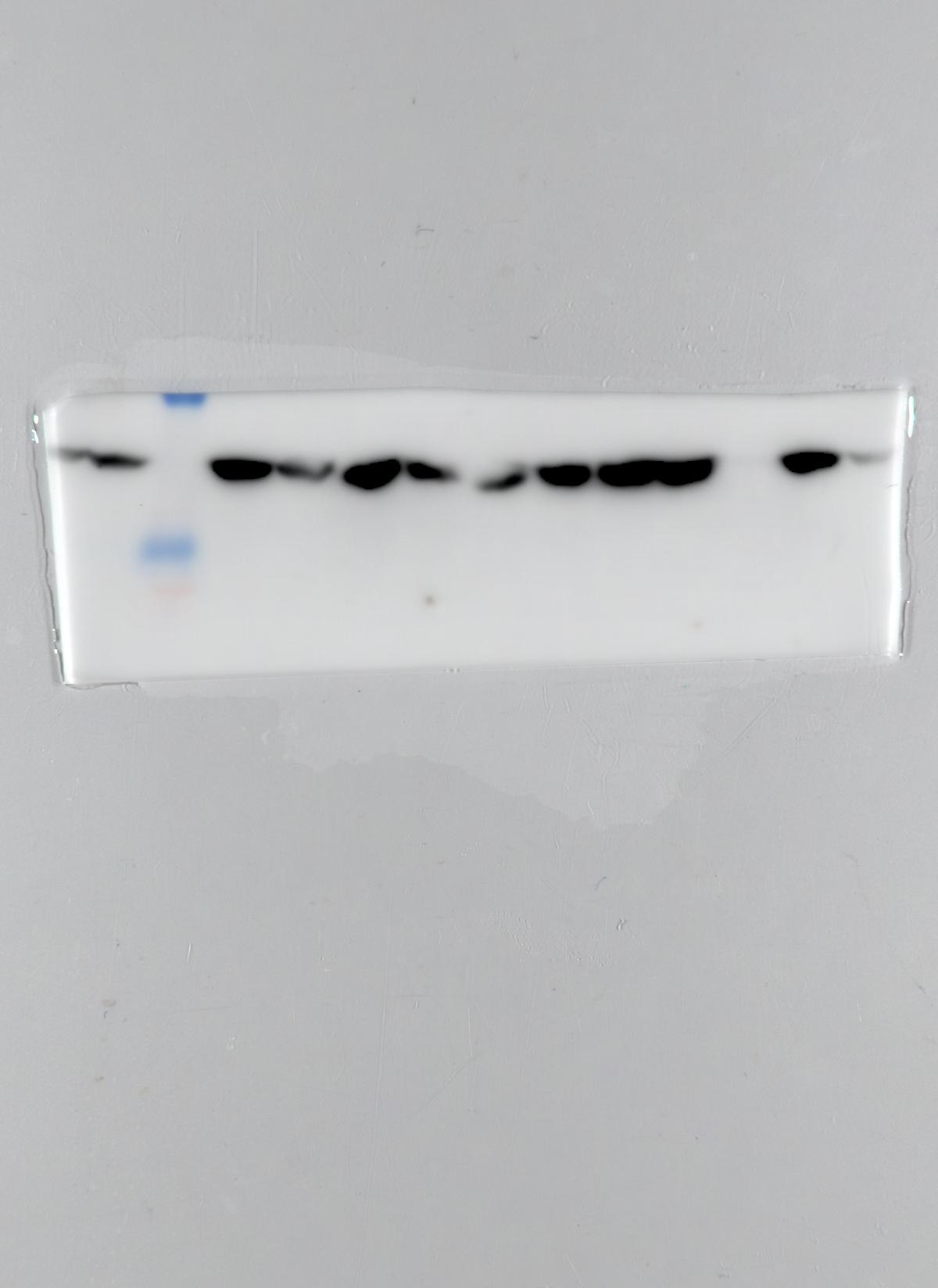

Supplement: Figure 5—figure supplement 1—source data 2. [file elife-89755-fig5-figsupp1-data2.zip › Figure 5-figure supplement 1A_jpeg/Fig5_FigSup1A_Input_3.jpg]

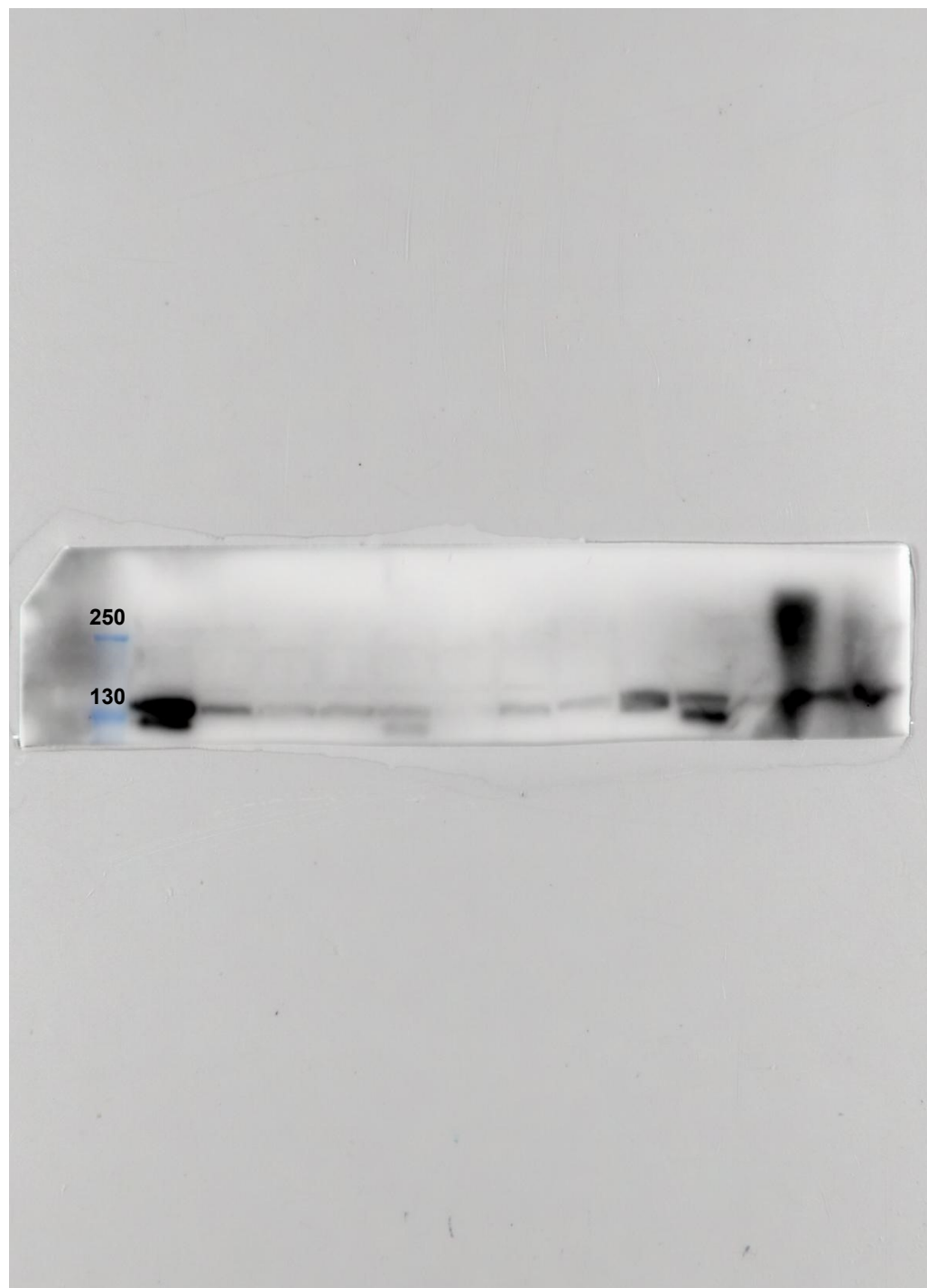

**GFP**  
**(GluK1 ~135 kDa)**

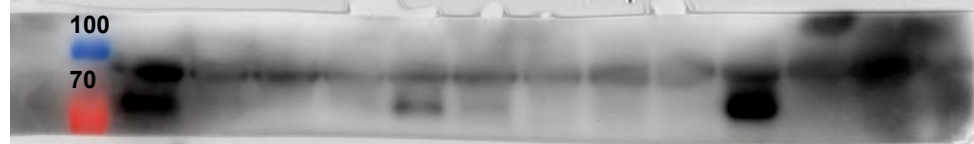

**GFP  
(Neto2  
~84 kDa)**

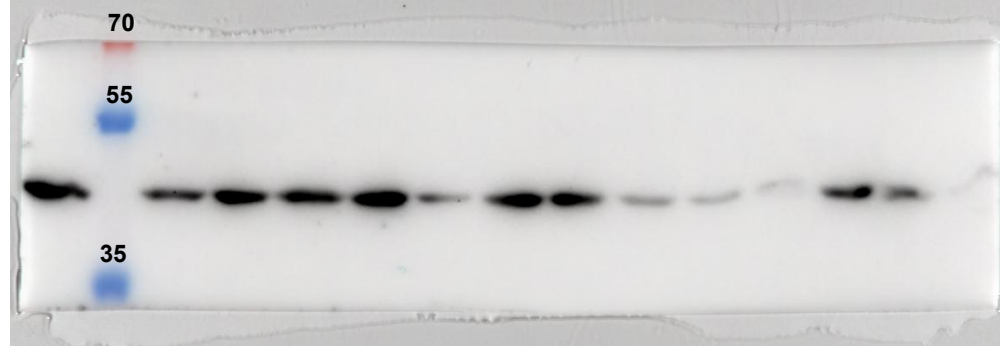

**Actin**  
**(~42 kDa)**

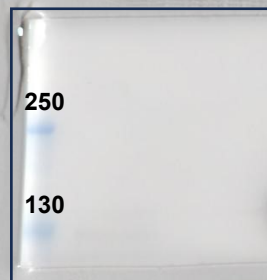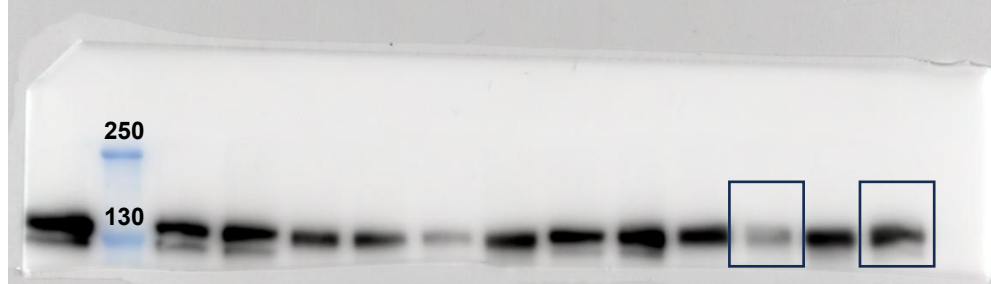

**GFP**  
(GluK1 ~135 kDa)

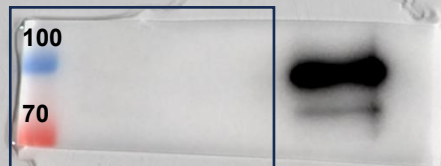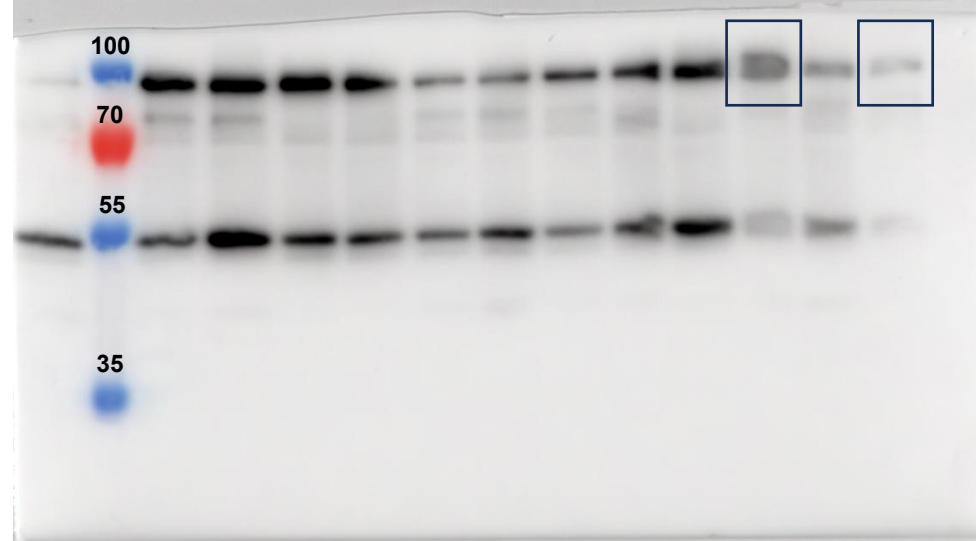

**GFP**  
(Neto2  
~84 kDa)

Supplement: Figure 5—figure supplement 1—source data 3. [file elife-89755-fig5-figsupp1-data3.pdf]

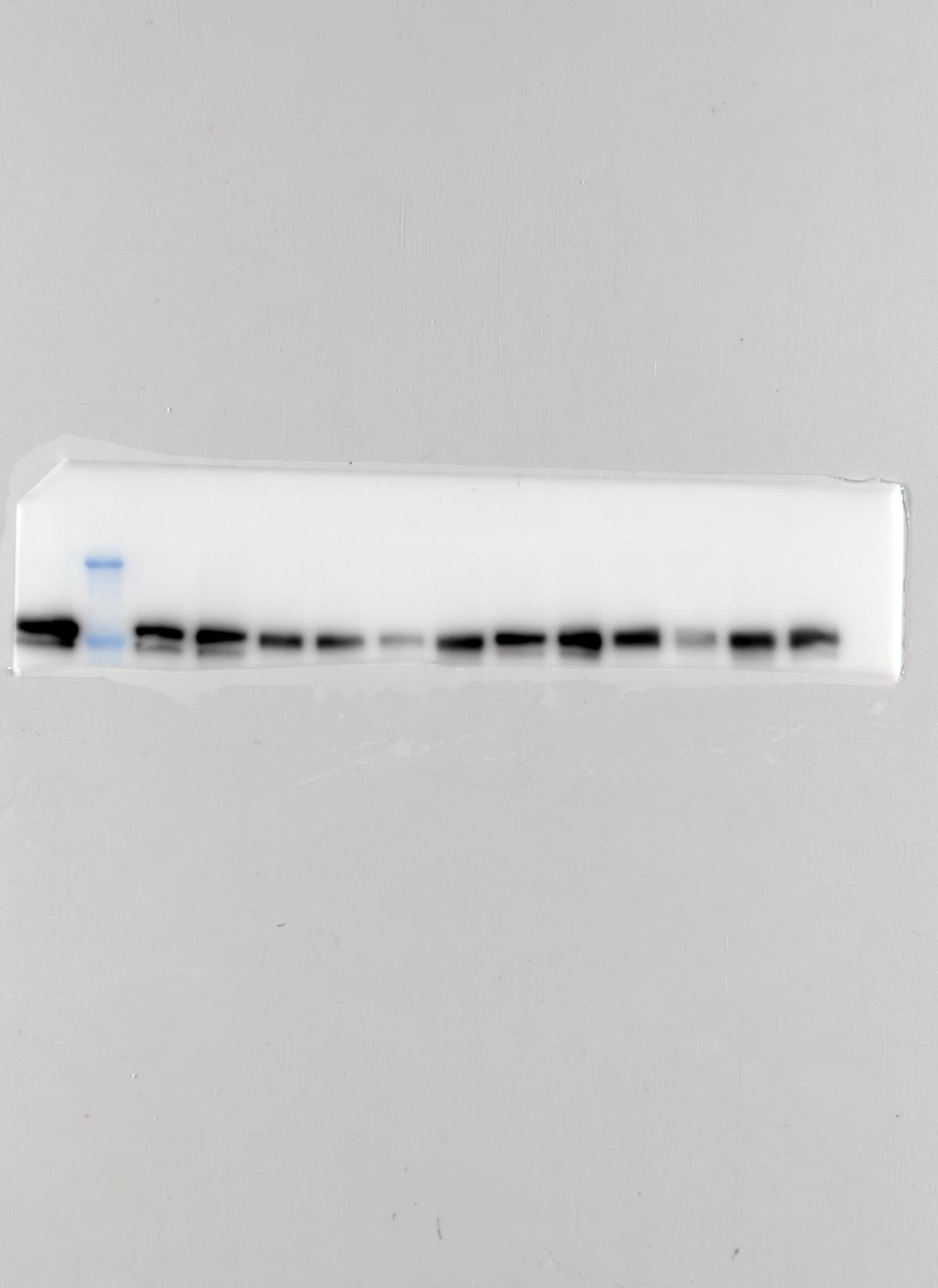

Supplement: Figure 5—figure supplement 1—source data 4. [file elife-89755-fig5-figsupp1-data4.zip › Figure 5-figure supplement 1B_jpeg/Fig5_FigSup1B_Eluate_1b.jpg]

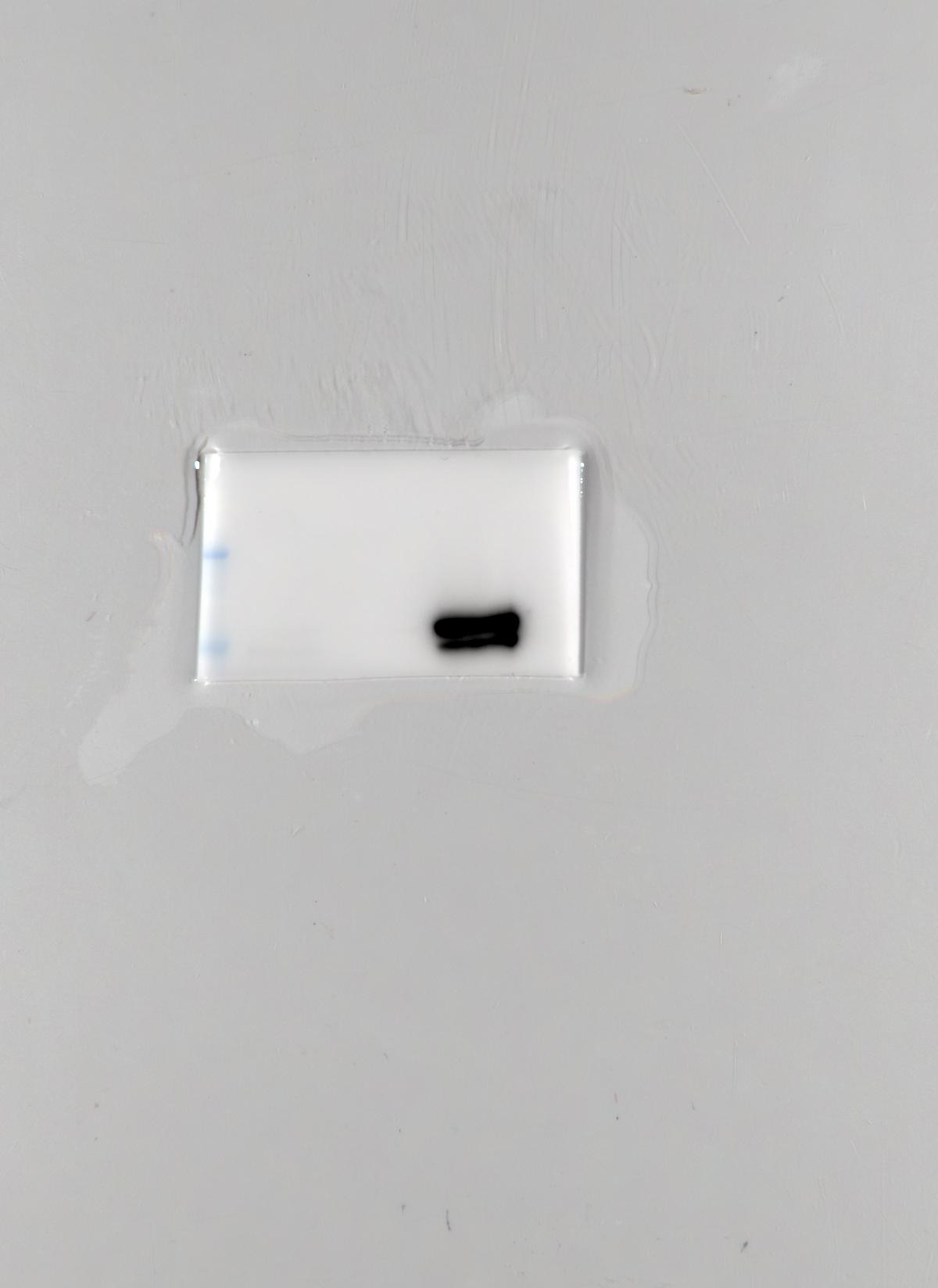

Supplement: Figure 5—figure supplement 1—source data 4. [file elife-89755-fig5-figsupp1-data4.zip › Figure 5-figure supplement 1B_jpeg/Fig5_FigSup1B_Eluate_1a.jpg]

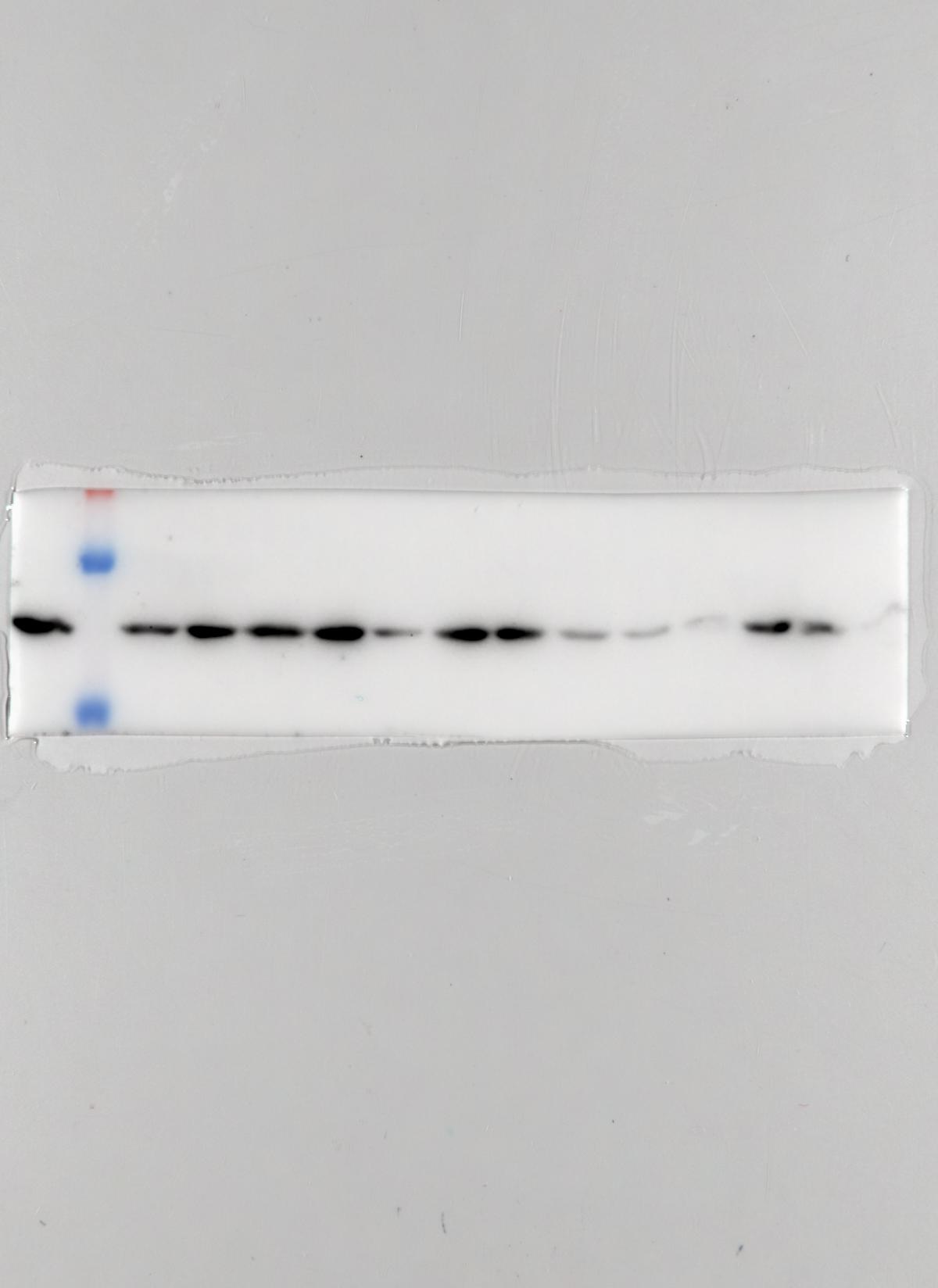

Supplement: Figure 5—figure supplement 1—source data 4. [file elife-89755-fig5-figsupp1-data4.zip › Figure 5-figure supplement 1B_jpeg/Fig5_FigSup1B_Input_3.jpg]

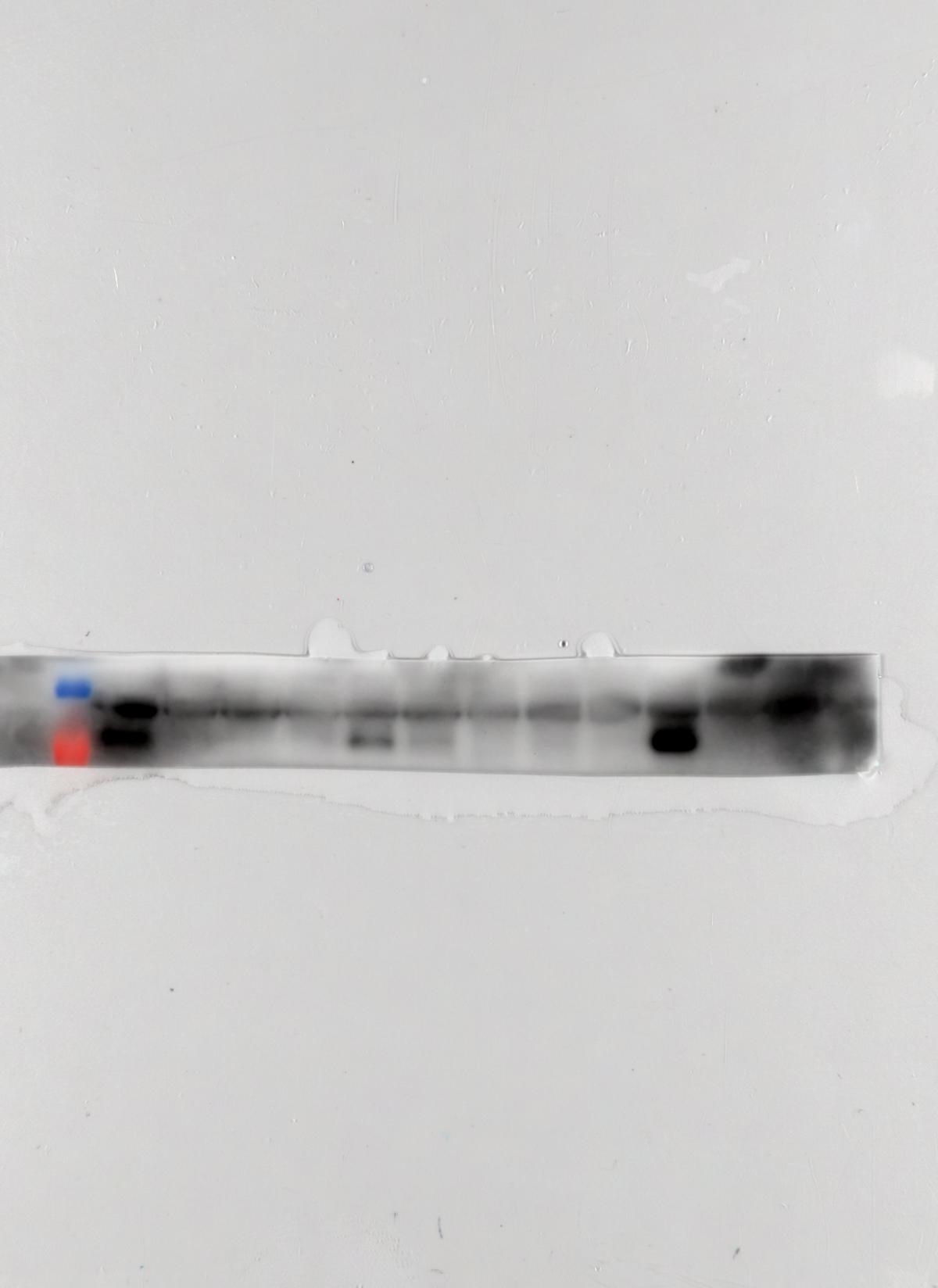

Supplement: Figure 5—figure supplement 1—source data 4. [file elife-89755-fig5-figsupp1-data4.zip › Figure 5-figure supplement 1B_jpeg/Fig5_FigSup1B_Input_2.jpg]

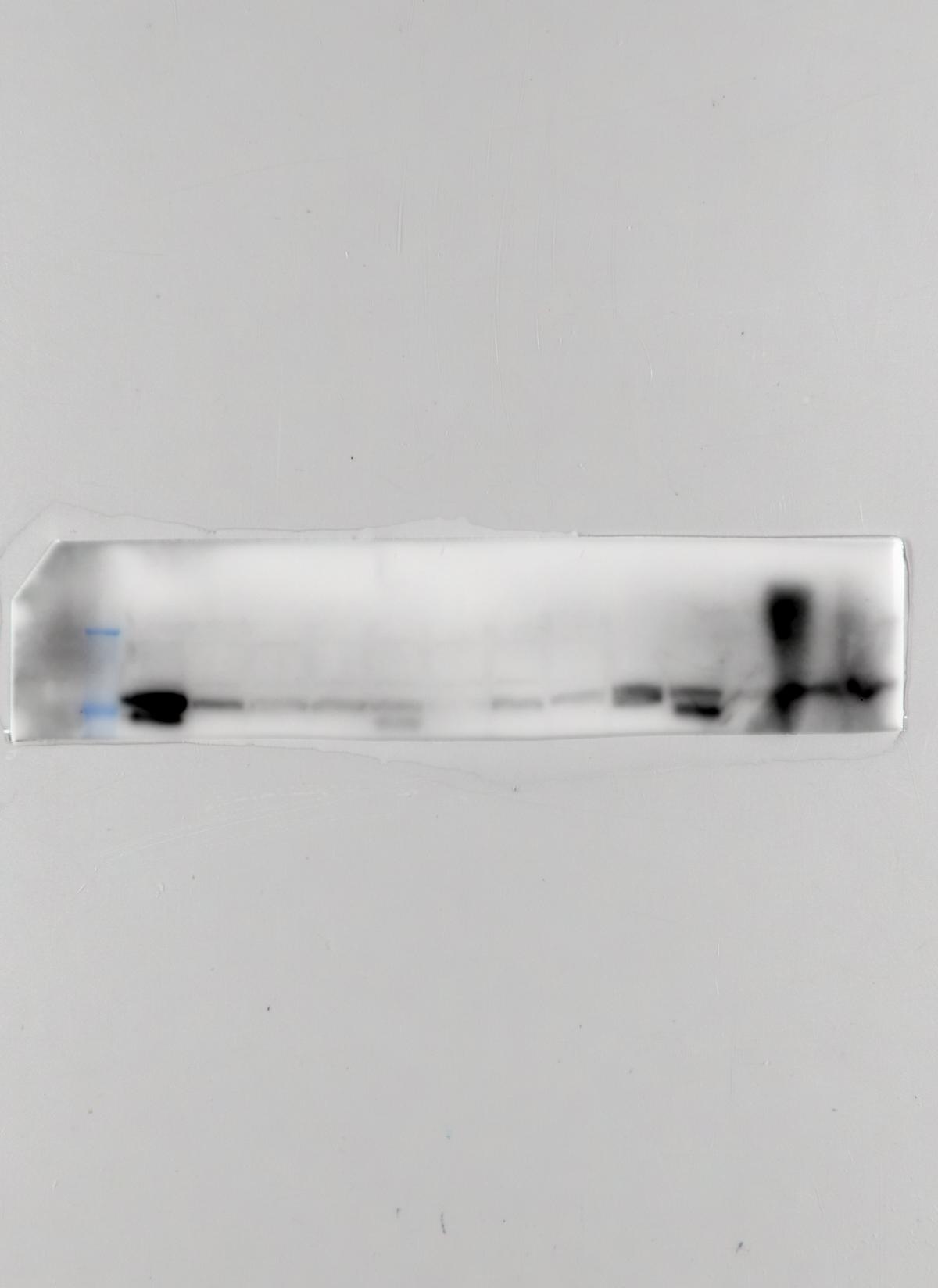

Supplement: Figure 5—figure supplement 1—source data 4. [file elife-89755-fig5-figsupp1-data4.zip › Figure 5-figure supplement 1B_jpeg/Fig5_FigSup1B_Input_1.jpg]

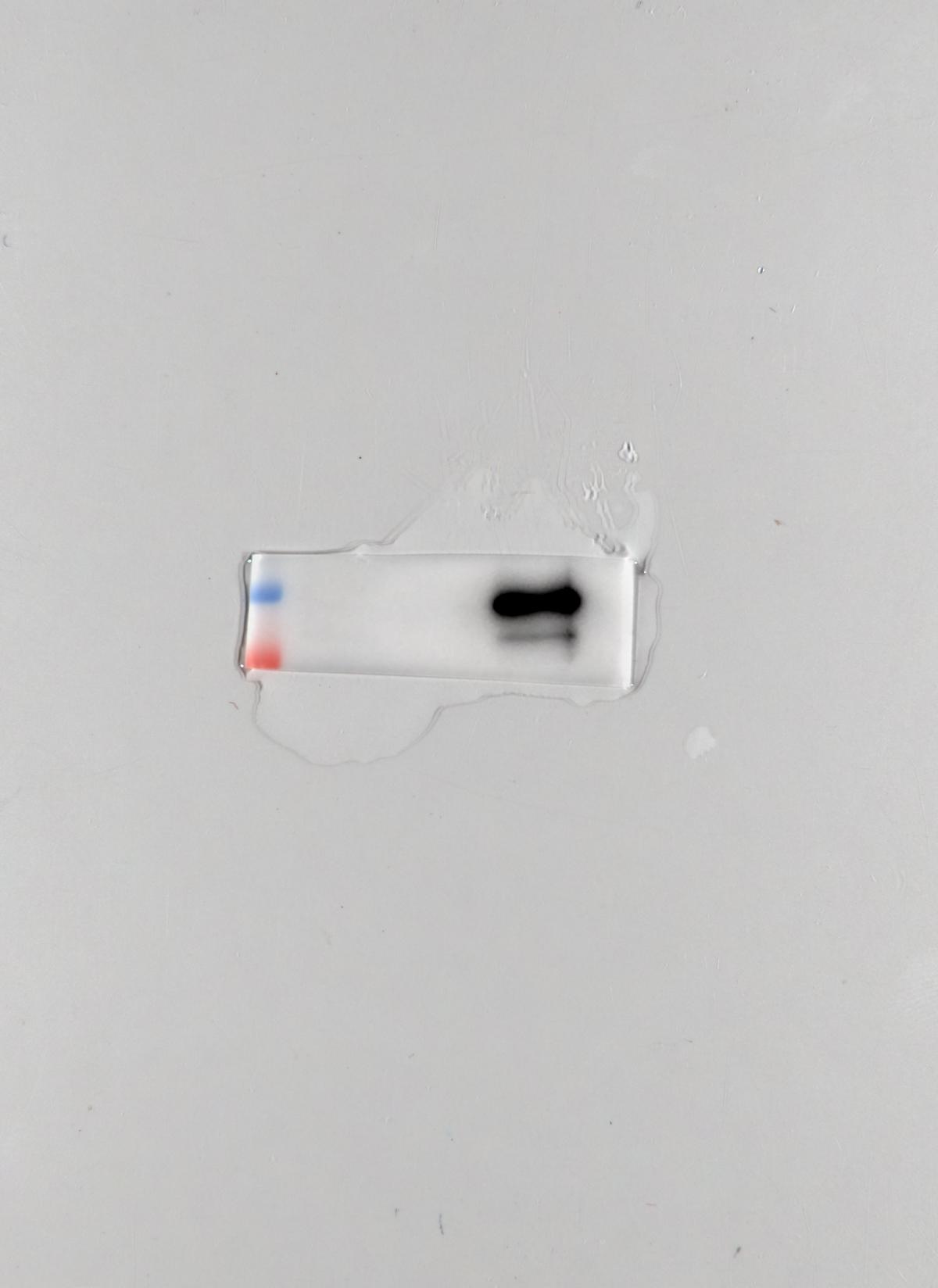

Supplement: Figure 5—figure supplement 1—source data 4. [file elife-89755-fig5-figsupp1-data4.zip › Figure 5-figure supplement 1B_jpeg/Fig5_FigSup1B_Eluate_2a.jpg]

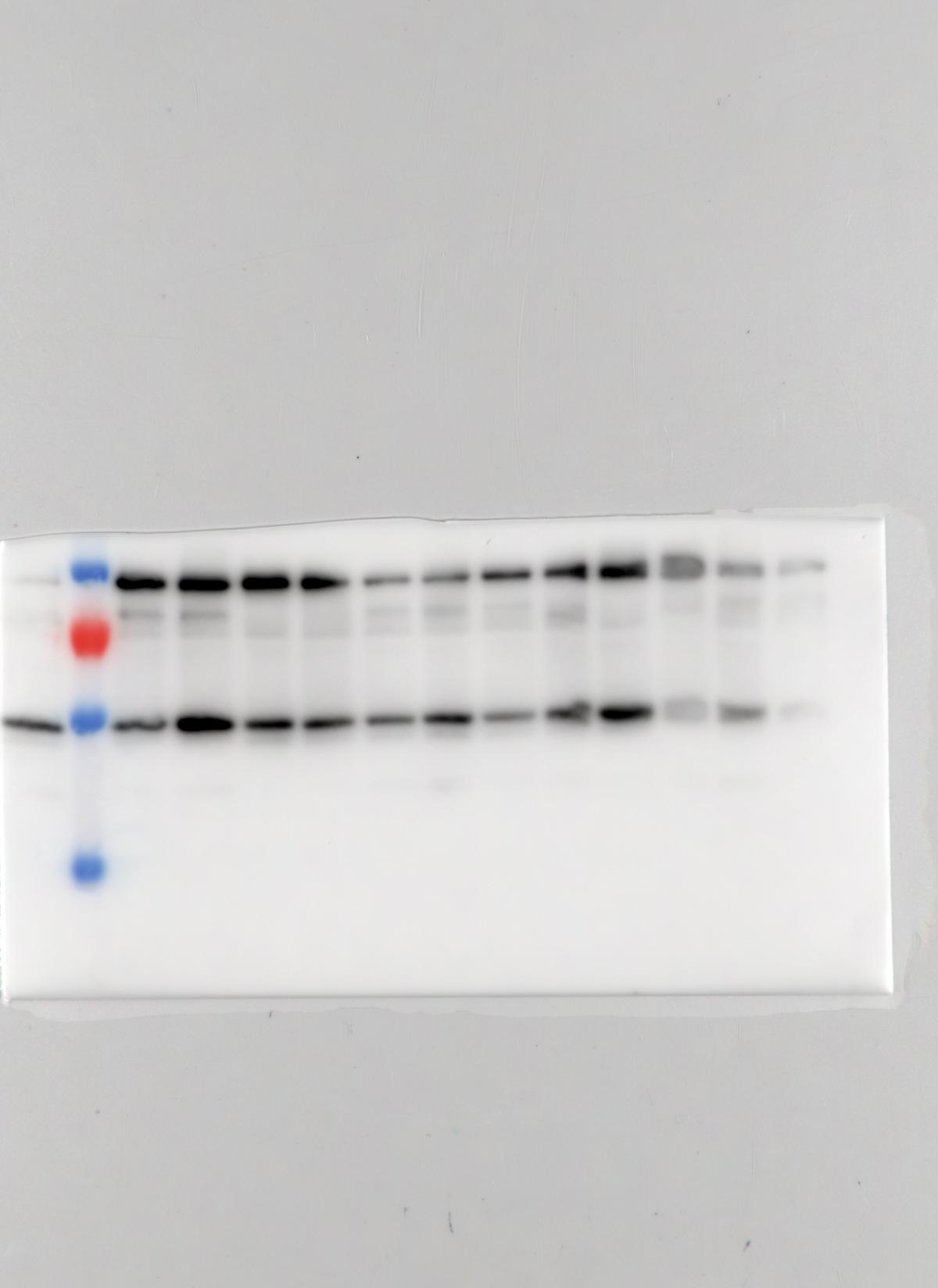

Supplement: Figure 5—figure supplement 1—source data 4. [file elife-89755-fig5-figsupp1-data4.zip › Figure 5-figure supplement 1B_jpeg/Fig5_FigSup1B_Eluate_2b.jpg]

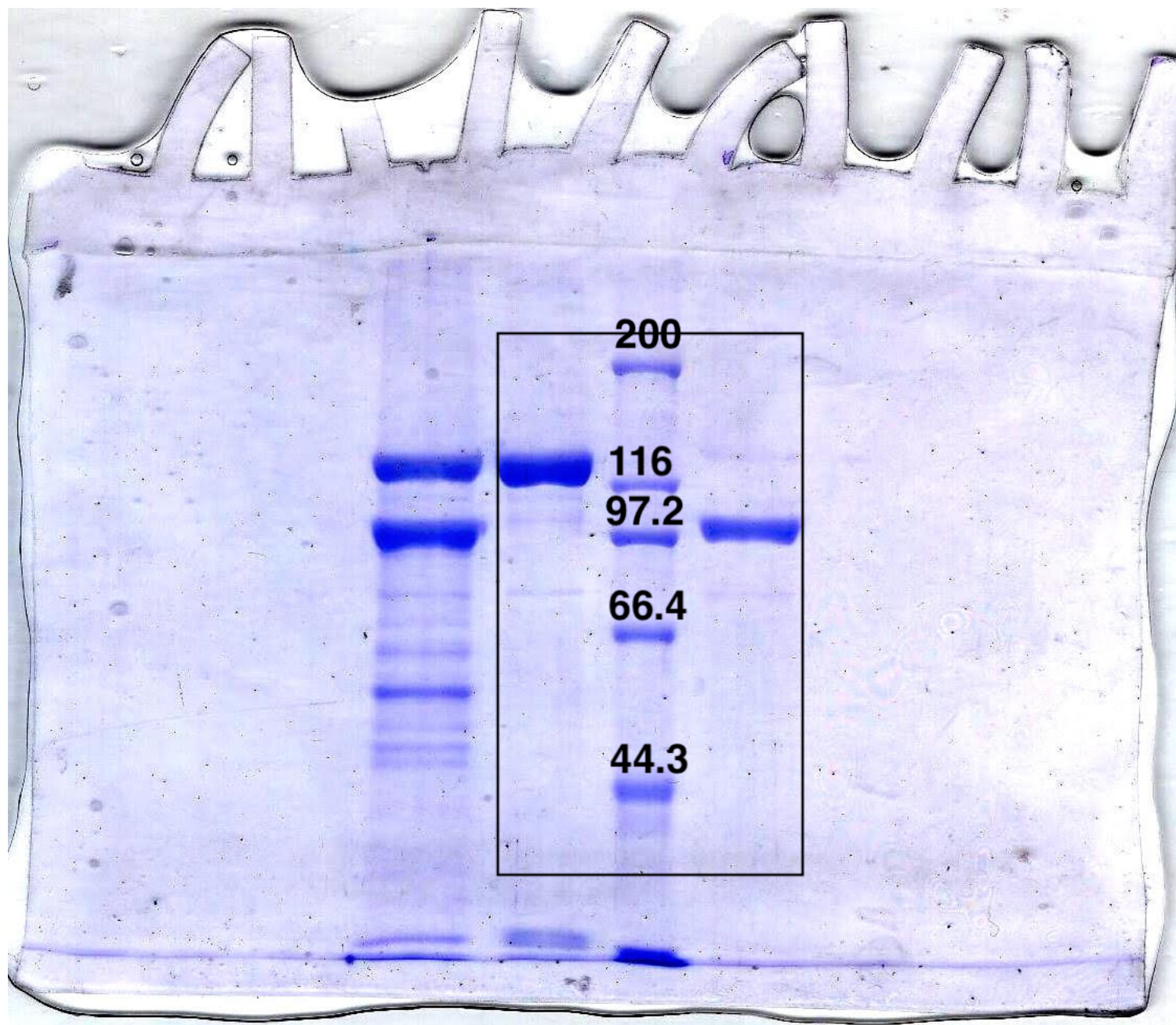

Supplement: Figure 6—figure supplement 2—source data 2. [file elife-89755-fig6-figsupp2-data2.zip › Figure 6-figure supplement 2B-source data 1/Figure 6-figure supplement 2Binset1-source data -1.pdf]

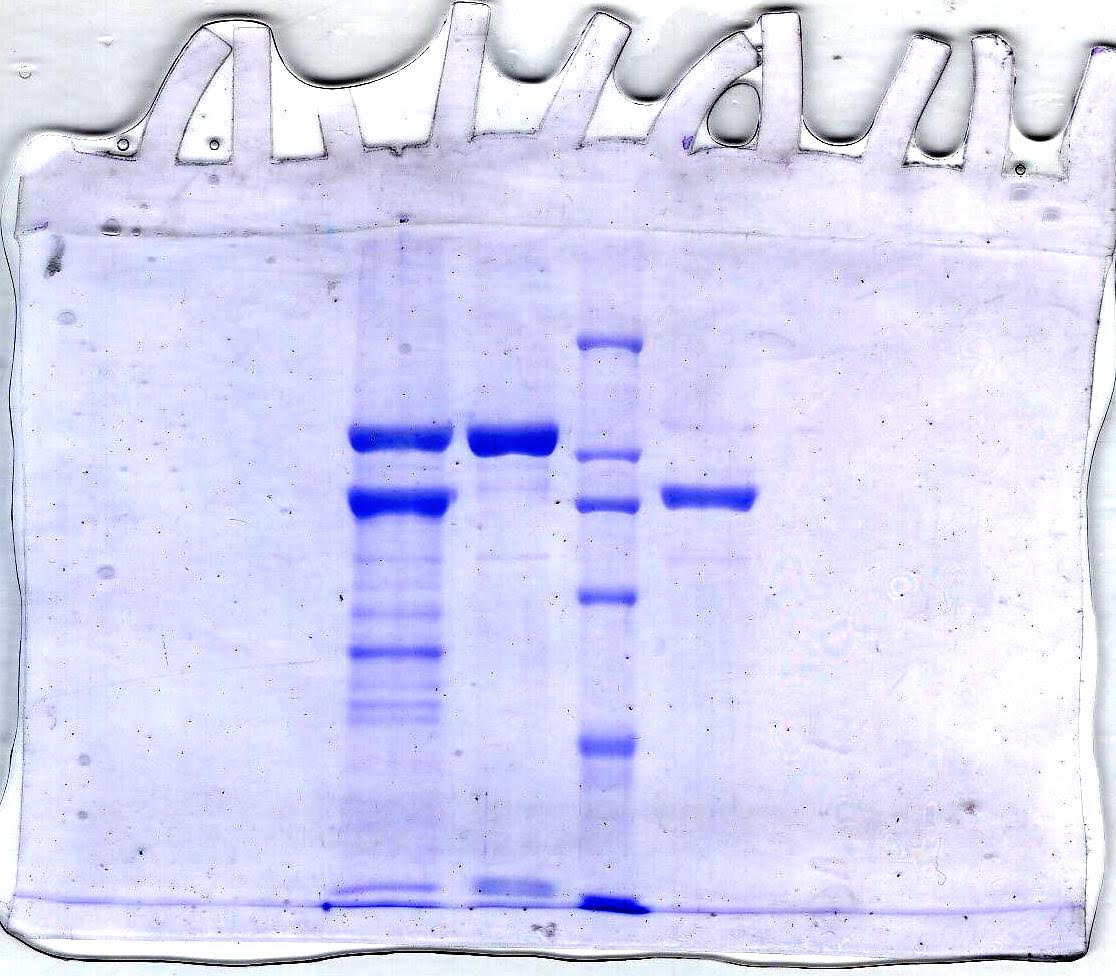

Supplement: Figure 6—figure supplement 2—source data 3. [file elife-89755-fig6-figsupp2-data3.zip › Figure 6-figure supplement 2B-source data 2/Figure 6-figure supplement 2Binset1-source data -2.jpg]

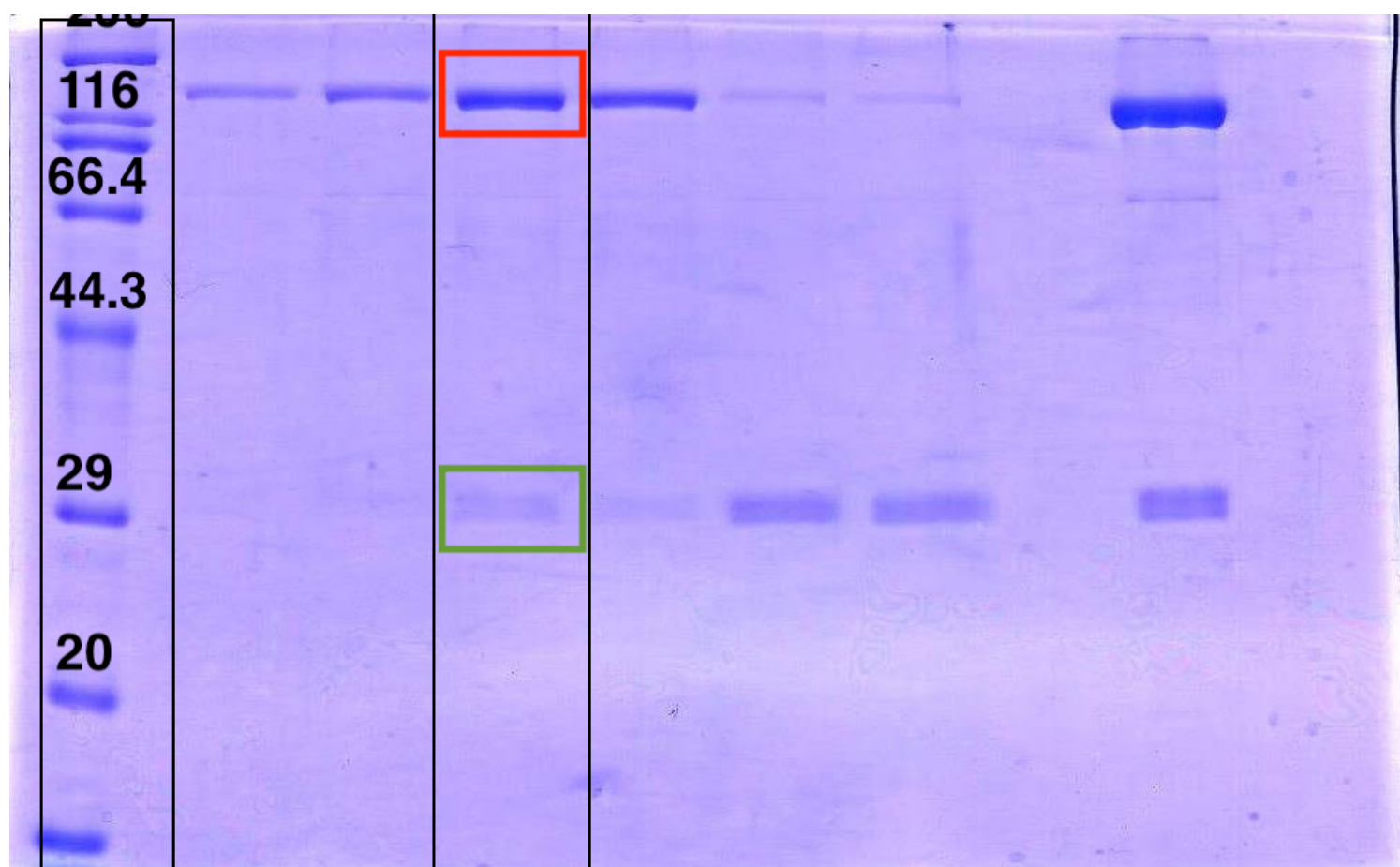

Supplement: Figure 6—figure supplement 2—source data 4. [file elife-89755-fig6-figsupp2-data4.zip › Figure 6-figure supplement 2B-source data 3/Figure 6-figure supplement 2Binset2-source data -1.pdf]

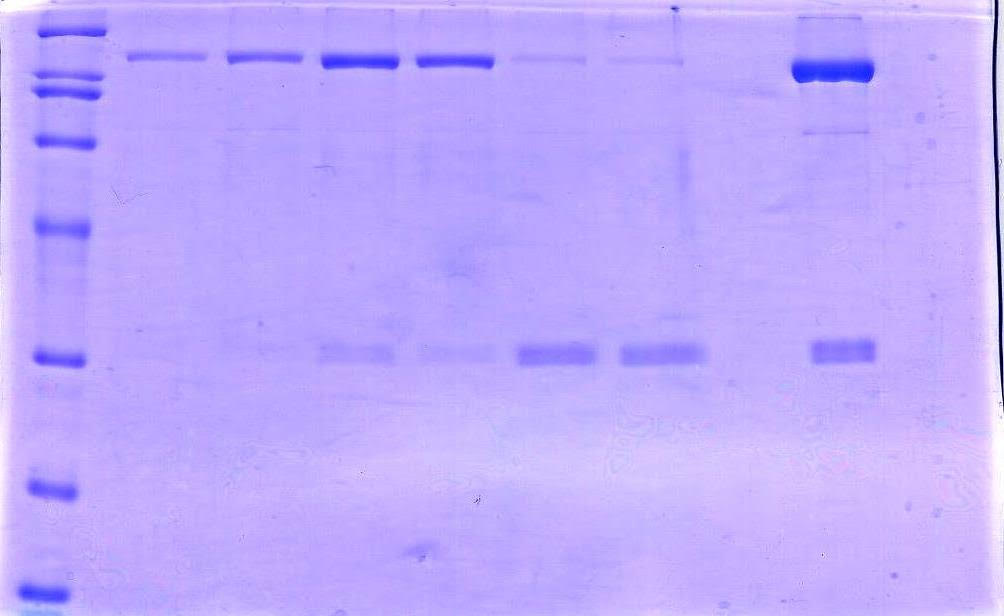

Supplement: Figure 6—figure supplement 2—source data 5. [file elife-89755-fig6-figsupp2-data5.zip › Figure 6-figure supplement 2B-source data 4/Figure 6-figure supplement 2Binset2-source data -2.jpg]

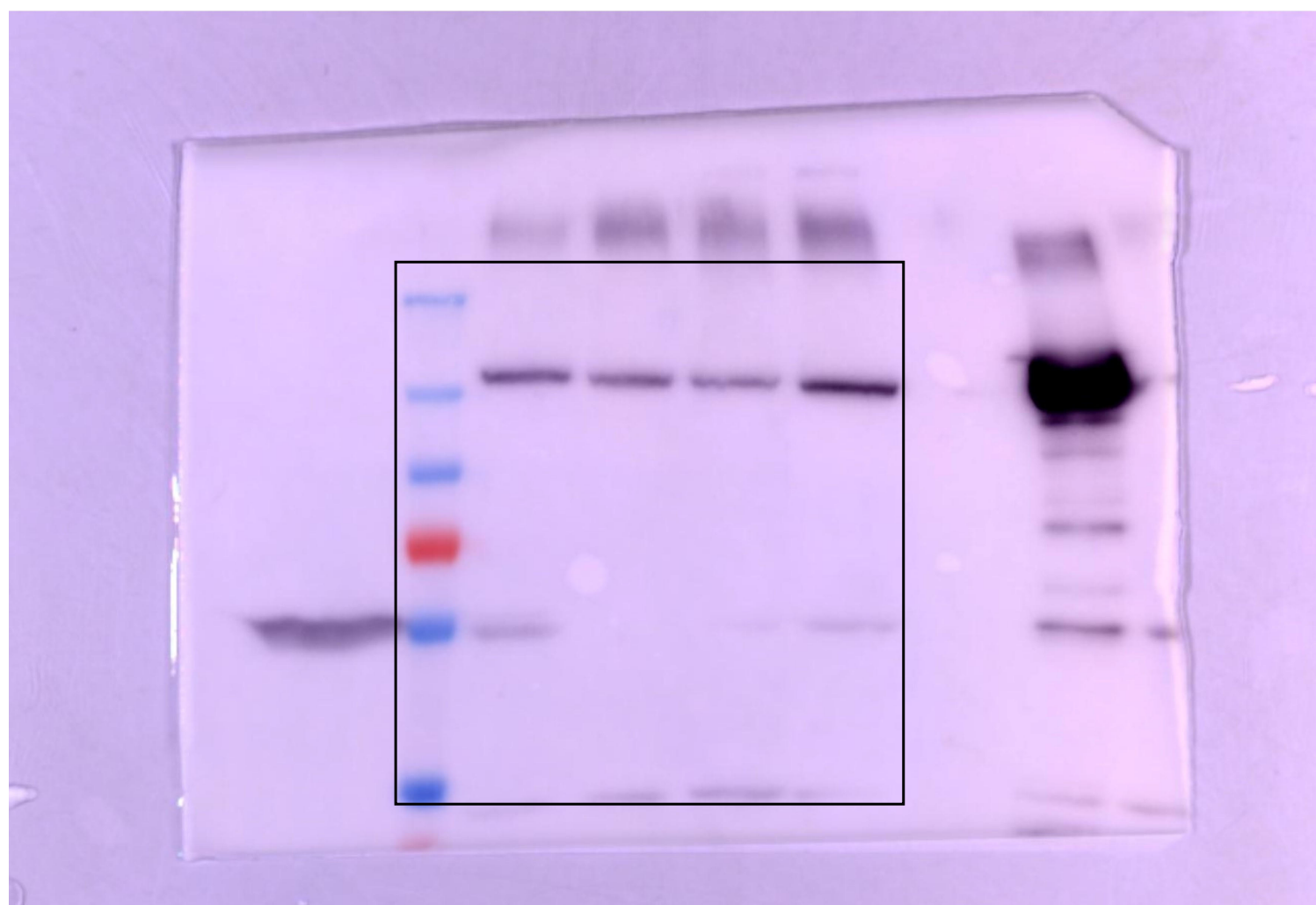

Supplement: Figure 6—figure supplement 3—source data 1. [file elife-89755-fig6-figsupp3-data1.zip › Figure 6-figure supplement 3A-source data 1/Figure 6-figure supplement 3A-source data 1.pdf]

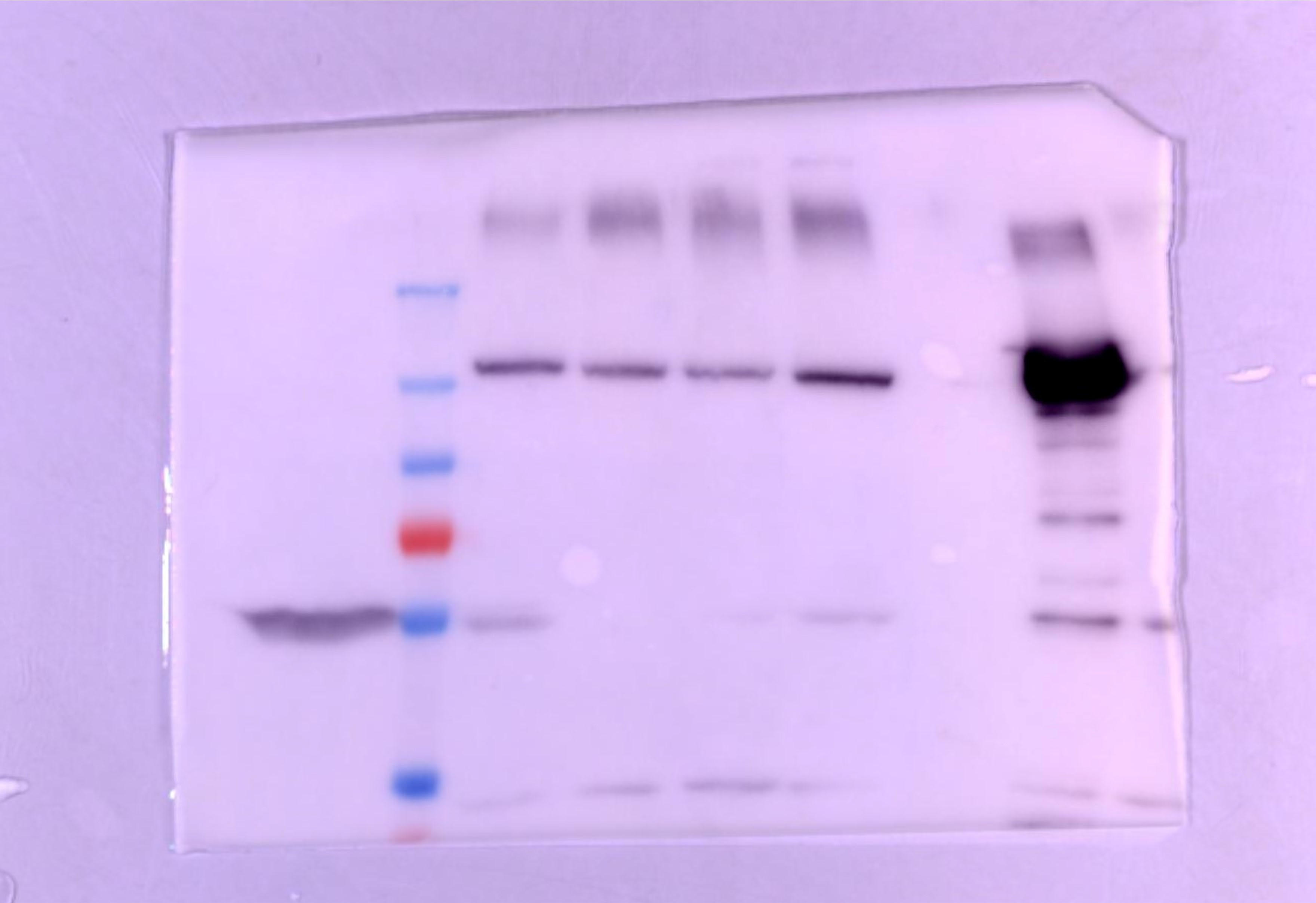

Supplement: Figure 6—figure supplement 3—source data 2. [file elife-89755-fig6-figsupp3-data2.zip › Figure 6-figure supplement 3A-source data 2/Figure 6-figure supplement 3A-source data 2.jpg]
